# Admixture-informed polygenic risk reporting using the ePRS framework

**DOI:** 10.1038/s41467-026-72457-x

**Published:** 2026-04-30

**Authors:** Yu-Jyun Huang, Nuzulul Kurniansyah, Matthew O. Goodman, Brian W. Spitzer, Jiongming Wang, Adrienne Stilp, Cecelia Laurie, Paul S. de Vries, Han Chen, Yuan-I Min, Mario Sims, Gina M. Peloso, Xiuqing Guo, Joshua C. Bis, Jennifer A. Brody, Laura M. Raffield, Jennifer A. Smith, Wei Zhao, Jerome I. Rotter, Stephen S. Rich, Susan Redline, Myriam Fornage, Robert Kaplan, Nora Franceschini, Daniel Levy, Alanna C. Morrison, Eric Boerwinkle, Nicholas L. Smith, Charles Kooperberg, Bruce M. Psaty, Sebastian Zöllner, the Trans-Omics for Precision Medicine Consortium, Jiongming Wang, Adrienne Stilp, Cecelia Laurie, Han Chen, Yuan-I Min, Mario Sims, Gina M. Peloso, Xiuqing Guo, Joshua C. Bis, Jennifer A. Brody, Laura M. Raffield, Jennifer A. Smith, Wei Zhao, Jerome I. Rotter, Stephen S. Rich, Susan Redline, Myriam Fornage, Robert Kaplan, Nora Franceschini, Daniel Levy, Alanna C. Morrison, Eric Boerwinkle, Nicholas L. Smith, Charles Kooperberg, Bruce M. Psaty, Sebastian Zöllner, Paul S. de Vries, Tamar Sofer, Tamar Sofer

**Affiliations:** 1 Department of Medicine, Harvard Medical School, Boston, MA USA; 2 CardioVascular Institute (CVI), Beth Israel Deaconess Medical Center, Boston, MA USA; 3 Department of Medicine, Brigham and Women’s Hospital, Boston, MA USA; 4 Department of Biostatistics, University of Michigan, Ann Arbor, MI USA; 5 Center for Statistical Genetics, University of Michigan, Ann Arbor, MI USA; 6 Department of Biostatistics, University of Washington, Seattle, WA USA; 7 Human Genetics Center, Department of Epidemiology, School of Public Health, The University of Texas Health Science Center at Houston, Houston, TX USA; 8 Department of Medicine, University of Mississippi Medical Center, Jackson, MS USA; 9 Department of Social Medicine, Population and Public Health, University of California at Riverside School of Medicine, Riverside, CA USA; 10 Department of Biostatistics, Boston University School of Public Health, Boston, MA USA; 11 The Institute for Translational Genomics and Population Sciences, Department of Pediatrics, The Lundquist Institute for Biomedical Innovation at Harbor-UCLA Medical Center, Torrance, CA USA; 12 Cardiovascular Health Research Unit, Department of Medicine, University of Washington, Seattle, WA USA; 13 Department of Genetics, University of North Carolina at Chapel Hill, Chapel Hill, NC USA; 14 Department of Epidemiology, School of Public Health, University of Michigan, Ann Arbor, MI USA; 15 Survey Research Center, Institute for Social Research, University of Michigan, Ann Arbor, MI USA; 16 Department of Genome Sciences, University of Virginia School of Medicine, Charlottesville, VA USA; 17 Brown Foundation Institute of Molecular Medicine, McGovern Medical School, University of Texas Health Science Center at Houston, Houston, TX USA; 18 Division of Public Health Sciences, Fred Hutchinson Cancer Research Center, Seattle, WA USA; 19 Department of Epidemiology & Population Health, Albert Einstein College of Medicine, Bronx, NY USA; 20 Department of Epidemiology, University of North Carolina, Chapel Hill, NC USA; 21 The Population Sciences Branch of the National Heart, Lung and Blood Institute, Bethesda, MD USA; 22 The Framingham Heart Study, Framingham, MA USA; 23 Kaiser Permanente Washington Health Research Institute, Seattle, WA USA; 24 Department of Epidemiology, University of Washington, Seattle, WA USA; 25 Seattle Epidemiologic Research and Information Center, Department of Veterans Affairs Office of Research and Development, Seattle, WA USA; 26 Division of Public Health Sciences, Fred Hutchinson Cancer Center, Seattle, WA USA; 27 Department of Psychiatry, University of Michigan, Ann Arbor, MI USA; 28 Department of Biostatistics, Harvard T.H. Chan School of Public Health, Boston, MA USA

**Keywords:** Genomics, Heritable quantitative trait, Genetic variation

## Abstract

Polygenic risk score values vary with genetic ancestry due to differences in population-specific allele frequencies and linkage disequilibrium patterns. We present a framework to calibrate polygenic risk scores based on ancestral makeup. We propose the “expected polygenic risk score” or ePRS, defined as the expected value of a polygenic risk score based on one’s global or local admixture patterns. We further define the “residual polygenic risk score” or rPRS as measuring the deviation of the polygenic risk score from the ePRS. The ePRS reflects the baseline ancestry-driven component of genetic risk, whereas the rPRS isolates an ancestry-agnostic measure of genetic liability. Simulation studies confirm that it suffices to adjust for ePRS to obtain nearly unbiased estimates of the polygenic risk score-outcome association without further adjusting for principal components. Using the TOPMed and the All of Us datasets, effect size estimates for the rPRS (adjusted for ePRS) are similar to those obtained from polygenic risk scores adjusting for genetic principal components. The ePRS framework can protect from population stratification in association analysis and provide an equitable strategy to interpret genetic risk across diverse populations.

## Abstract (web summary)

Polygenic risk scores vary with genetic ancestry. Here, the authors develop a framework to calibrate for genetic ancestry in these scores that leverages admixture analysis, separating out the ancestry-specific component of polygenic risk scores.

## Introduction

Polygenic risk scores (PRS) combine information from multiple genetic variants, summarizing disease risk due to genetics into a single score. Potential applications of PRS in healthcare settings include disease risk screening, risk prediction, and identification of target populations that may benefit from early interventions; all are the ultimate goals of precision and preventive medicine^1–3^. A critical challenge in applying a PRS is that the potential values of PRS in an individual depend on that individual’s genetic ancestry^4–6^. In other words, PRS distribution varies by genetic ancestry. This is true even for PRSs that are predictive across populations of diverse ancestries. Recent initiatives that explored the clinical use of PRSs addressed this issue by reporting an “ancestry-adjusted” PRS: the residual of a PRS regression over genetic principal components (PCs)^7–9^. This approach has been developed and applied also in population-based studies without clinical reporting^2,10,11^, and has been implemented in tools such as the PGS Catalog calculator.^12^

Genetic PCs constructed using genome-wide data have been long used to summarize population structure in an unsupervised manner^13,14^, and later to correct for population stratification bias in genetic association studies^15,16^, i.e., by adjusting association models for PCs. Using PCs as covariates is now a standard procedure in genome-wide association studies (GWAS)^16^, as well as when estimating PRS associations with an outcome^17^. Ancestry-adjusted PRS obtained by regression of PRS over PCs is another use of genetic PCs that relies on population structure captured by PCs. There are several criticisms of using genetic PCs. The number of PCs included in association analyses varies across studies, and PCs are not directly comparable between datasets. Moreover, PCs capture only global genetic structure and may miss local ancestry patterns, raising uncertainty about whether this adjustment sufficiently addresses ancestry-related confounding in admixed populations^18–20^. Also, the PRS adjustment method, which requires regressing PRS on PCs is less suitable for analyses with small sample sizes, where PCs are less accurate^14,21,22^.

This work is motivated by the challenges of applying PCs in translating PRSs for clinical use in diverse populations, and by the opportunity to use admixture analysis—inference of proportions of the genome (global ancestry) as well as of specific genomic intervals (local ancestry) inherited from pre-specified ancestral population. Concepts of global and local ancestry have been recently popularized with increasing studies of admixed populations^23–25^, and communication of ancestral decomposition may be simpler, in comparison to PCs, due to increasing use of ancestry testing in direct to consumer genetic services such as 23andMe^26^. Here, we propose a method to separate the ancestry-specific from the individual-level, ancestry-agnostic component of the PRS while using admixture inference. Just like the inference of ancestry admixture relies on population differences in allele frequencies and linkage disequilibrium (LD) patterns, so does our proposed approach. Specific ancestry decomposition of an individual implies their ancestry-specific potential PRS value. The difference between the actual PRS value and this ancestry background-specific PRS is the component that is unique to the individual, beyond what is expected from someone with the ancestry composition that the individual has.

Based on this rationale, we introduce a framework that incorporates an individual’s genetic ancestry composition into PRS analysis pipeline. It is worth noting that the goal of this framework is not to select variants or update effect size weights for PRS calculation; rather, it can be integrated with existing PRS development strategies, as it addresses the application, rather than the development, of PRSs in ancestry-heterogeneous populations. We propose a metric, the expected PRS (ePRS), in which the expected value of a PRS, i.e., the ancestry-specific component, is derived from either global ancestry proportions or local ancestry patterns. We also derive the analytical form for both global ancestry and local ancestry versions of the ePRS. By definition, the ePRS can be interpreted as the baseline trait-specific genetic parameter based on an individual’s ancestral composition. Coupled with ePRS, we introduce another two metrics: the residual PRS (rPRS), which measures the person-specific deviation of the PRS from its expected mean according to admixture patterns (i.e., from the ePRS), and the quantile PRS (qPRS), defining the distribution-based percentile of the PRS accounting for both the ePRS and its variability. Specifically, the rPRS serves as an ancestry-adjusted PRS measurement, which is an ancestry-agnostic quantification of genetic risk, whereas the qPRS reflects the relative position of an individual’s genetic liability within the context of their ancestral background. Therefore, the rPRS and qPRS can distinguish genetic risk between individuals regardless of their genetic ancestry patterns.

We performed simulation studies to examine whether our proposed method can provide a nearly unbiased estimation of the PRS-outcome effect by adjusting for ePRS in the association model. The PC-based adjustment procedures are considered as comparators. We applied our method to analyze data from the Trans-Omics for Precision Medicine (TOPMed) study to compare the PRS-outcome effect estimation with the standard PC-adjusted approach, and to evaluate risk stratification using the qPRS. We also implemented the ePRS framework in the All of Us (AoU) research program to analyze six cardiovascular disease (CVD)-related traits. In the AoU analysis, we demonstrate how the ePRS itself captures the existence of ancestry-related confounders, in line with simulation results. In all, our simulations and analyses demonstrate that the ePRS can (1) replace genetic PCs adjustment in PRS association analyses; (2) facilitate the decomposition of ancestry- from individual (ancestry-agnostic)-baseline genetic effects; and (3) capture the existence of unknown, ancestry-enriched, genetic determinants of the relevant trait.

## Results

### PRS-outcome association model

The underlying PRS-outcome association model is provided by:

1 $$g\left(E(Y)\right)={\beta }_{0}+{{{{\boldsymbol{X}}}}}^{T}{{{{\boldsymbol{\delta }}}}}_{x}+{\beta }_{1}\times {PRS}+\gamma \times {U}_{G}$$

In Eq. (1), $Y$ is the trait of interest. The PRS of individual $i,{{PRS}}_{i}={\sum }_{j=1}^{p}{\omega }_{j}\times {g}_{{ij}}$, is calculated by the aggregation of SNP allele effect sizes ${\omega }_{j},j={{\mathrm{1,2}}},\ldots,p$ and the individual allele counts ${g}_{{ij}},j={{\mathrm{1,2}}},..,p$. To focus on the phenomenon of interest (population stratification), in this work, we assume that the SNP effect size ${\omega }_{j}$, are fixed values, and this work does not discuss uncertainty in their estimation nor potential differences in effects across genetic ancestries. The parameter of interest is ${\beta }_{1}$, representing the effect size of the PRS-outcome association; ${{{\boldsymbol{X}}}}$ is a vector of covariates and ${{{\boldsymbol{\delta }}}}$ is the vector of corresponding effect sizes; and ${U}_{G}$ is an unknown ancestry-related genetic factor, which may confound the PRS-outcome relationship (population stratification bias), and $\gamma$ is its effect. The function $g\left(\cdot \right)$ is known as the link function, relating the outcome distribution to the covariates. Note that the PRS in Eq. (1) may not be the “perfect PRS”, i.e., it may not represent the entire additive genetic effects on trait $Y$. It is merely the modeled PRS, developed based on known genetic associations. Our assumption is that there are additional unknown genetic effects that operate on the trait via the unmeasured ancestry-related factor ${U}_{G}$. The rationale behind ${U}_{G}$ is that GWASs have been primarily performed in population of European ancestries. Therefore, trait-associated variants that are rare in European ancestries but are common or have large effects in other genetic ancestries may still be unknown and not incorporated into a PRS. Even though the PRS in Eq. (1) is not the ideal PRS, we still want to be able to estimate its association with the trait in all populations.

### Overview of the ePRS framework

The ePRS framework is grounded in the statistical concept of mixture distribution, where the underlying assumption is that an individual genome is inherited as a mixture of genomic intervals that each could be traced to a defined ancestral population. In each ancestral population, each variant has a frequency, and, correspondingly, a distribution. Because a PRS is a weighted sum of alleles, and each allele has a potentially different frequency across different ancestral populations, the ancestry background-specific value of a PRS in an individual depends on their ancestry composition. Accordingly, we define the ePRS statistically as the expectation of individual’s polygenic risk score ${{ePRS}}_{i}=E({{PRS}}_{i})$. Practically, it is a weighted sum of the allele frequencies in the mixture population of each individual, with the weights being the variant weights of a given PRS. We introduce two versions of the ePRS: one version relies on global genetic ancestry, where the mixture of ancestry is defined over the entire genome, and one version relies on local ancestry, allowing for different mixture of ancestries in each variant. The statistical model is provided as follows.

To model the allele count of each variant and each individual, we assume that ${g}_{{ij}}$ follows a mixture distribution based on individual $i$’s ancestry makeup:

2 $${g}_{{ij}} \sim {\sum }_{k=1}^{K}{\pi }_{{ik}}\times {P}_{k}({g}_{j};{f}_{j}^{{a}_{k}}),$$

with $K$ being the number of genetic ancestries ${a}_{i}\in \left\{{a}_{1},{a}_{2},\ldots,{a}_{K}\right\}$; ${f}_{j}^{{a}_{k}}$ is the ancestry-specific allele frequencies of allele $j$ in ancestry $k$, $j={{\mathrm{1,2}}},\ldots,p$ and $k={{\mathrm{1,2}}},\ldots,K$;${P}_{k}({g}_{j};{f}_{j}^{{a}_{k}})$ is the Binomial distribution ${Bin}\left(2,{f}_{j}^{{a}_{k}}\right)$; ${\pi }_{{ik}}$ represents the proportion of the entire genome of individual $i$ inherited from ancestral population $k,$ i.e., the global ancestry proportion. We assume that ${\pi }_{{ik}}$ are known. Note that if ${\pi }_{{ik}}=1$ for a specific $k\in \{1,\ldots,K\}$, Eq. (2) reduces to a standard allele count model without admixture. We can use global ancestry proportions to calculate the “global” ePRS (gePRS) as:

3 $$\begin{array}{c}{{gePRS}}_{i}=\mathop{\sum }_{j=1}^{p}{\omega }_{j}\times \left\{{\sum }_{k=1}^{K}2\times {f}_{j}^{{a}_{k}}\times {\pi }_{{ik}}\right\}\\={\sum }_{k=1}^{K}{\pi }_{{ik}}\times \left\{{\sum }_{j=1}^{p}{2\times \omega }_{j}\times {f}_{j}^{{a}_{k}}\right\}\end{array}$$

Thus, for individual $i$, the global ePRS is a weighted combination of ancestry-specific ePRSs, with weights being their global ancestry proportions.

If the ancestry makeup is available in each locus (local ancestry), we can also derive local ePRS (lePRS). Assuming that each chromosomal copy’s local ancestry is known and fixed, we now use the binomial distribution with count of 1 for chromosomal copy *m* = 1,2, i.e., ${g}_{{ijm}}|{a}_{{ijm}}={a}_{k} \sim {Ber}(1,{f}_{j}^{{a}_{k}})$. Exploiting the same strategy of deriving global ePRS, the lePRS can be written as

4 $${{lePRS}}_{i}={\sum }_{j=1}^{p}{\omega }_{j}\times ({f}_{j}^{{a}_{{ij}1}}+{f}_{j}^{{a}_{{ij}2}})$$

After computing the ePRS, we can derive the ancestry-adjusted PRS measurement, which we name the rPRS. We define rPRS as the difference between PRS and ePRS, i.e., ${rPR}{S}_{i}={PR}{S}_{i}-{ePR}{S}_{i}$. Conceptually, the rPRS provides an ancestry-adjusted measure that enables direct comparison of genetic risk across individuals with different ancestry compositions.

### PRS-outcome working association model under the ePRS framework

We now introduce another statistical concept: the “working association model”. The working association model acknowledges that, with the word “working”, it may not be the real underlying model. Notably, in any data analysis, the association model is a working association model. Here, we make this explicit and account for it in studying the methodology (see simulation studies). Specifically, we assume that the PRS that we have may not accurately capture all additive genetic effects on a trait in all individuals due to the unknown ancestry-related genetic effects encapsulated in ${U}_{G}$ in Eq. (1). The working association model in the ePRS framework is illustrated in Eq. (5)

5 $$g\left(E(Y)\right)={\widetilde{\beta }}_{0}+{{{{\boldsymbol{X}}}}}^{T}{\widetilde{{{{\boldsymbol{\delta }}}}}}_{x}+{\widetilde{\beta }}_{1}\times {rPRS}+\varphi \times {ePRS}$$

**Fig. 1:**
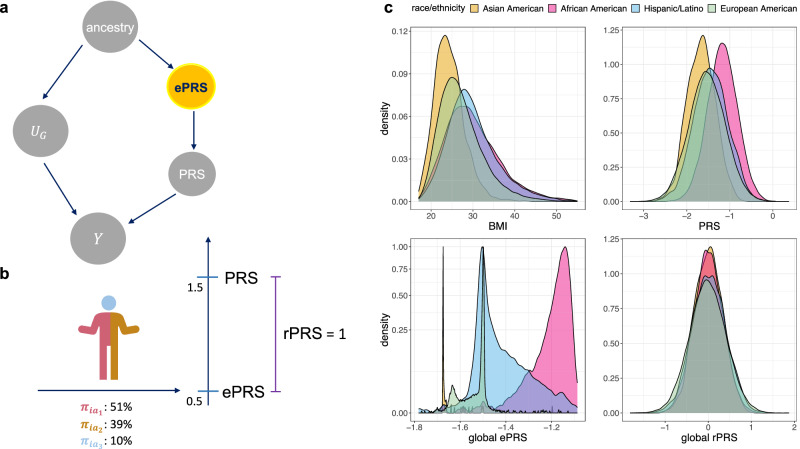
Schematic overview of the ePRS framework. **a** Directed acyclic graph demonstrating that the ePRS blocks a pathway between the PRS and unknown confounding genetic measures U_G. **b** The rPRS is computed by subtracting the value of the ePRS from that of the PRS. The ePRS is determined according to an individual’s ancestral makeup. **c** The distribution of BMI and its PRS, ePRS, and rPRS in the TOPMed dataset. The distributions stratified based on harmonized self-reported race/ethnicity. BMI body mass index, PRS polygenic risk scores, ePRS expected polygenic risk scores, rPRS residual polygenic risk scores, TOPMed Trans-Omics for Precision Medicine.

One can see that both the rPRS and the ePRS are used as covariates in the model. The major difference in this model compared to commonly used working association models is that we use the ePRS, whereas existing models typically adjust for multiple genetic PCs. As we show later, the estimated effect of the rPRS is equivalent to the estimated effect of the PRS in a standard association model that adjusts for genetic PCs. It is important to emphasize that the underlying PRS-outcome association model (Eq. (1)) differs from the working association model presented in Eq. (5). In particular, the working model does not have the unknown ancestry-related genetic factor ${U}_{G}$. Importantly, the ePRS is used to control for stratification bias induced by ${U}_{G}$, instead of the standard adjustment for genetic PCs, allowing for estimation of ${\widetilde{\beta }}_{1}$ as an appropriate estimate of ${\beta }_{1}$ from the underlying model (1). As illustrated in Fig. 1a, one can see that the ePRS controls for confounding by ${U}_{G}$ (population stratification bias) by, using causal inference terminology, “blocking the backdoor path” from the ${U}_{G}$ from Eq. (1).

### Quantification of population-level genetic risk under the ePRS framework

The ePRS and rPRS rely solely on allele frequencies as average values. In practice, distribution of a PRS also has a variance, which relates to allele frequencies, as well as to LD between variants used in the PRS. If the $p$ variants are independent, the variance of the PRS conditional on the global ancestry proportions is:

6 $${Var}\left({{PRS}}_{i}\right)={\sum }_{j=1}^{p}{\left({\omega }_{j}\right)}^{2}\times \left\{{\sum }_{k=1}^{K}2\times {f}_{j}^{{a}_{k}}\times (1-{f}_{j}^{{a}_{k}})\times {\pi }_{{ik}}\right\}$$

The variance of PRS based on local ancestry is provided in Eq. (7):

7 $${Var}\left({{PRS}}_{i}\right)={\sum }_{j=1}^{p}{\left({\omega }_{j}\right)}^{2}\times \left\{{f}_{j}^{{a}_{{ij}1}}\times \left(1-{f}_{j}^{{a}_{{ij}1}}\right)+{f}_{j}^{{a}_{{ij}2}}\times \left(1-{f}_{j}^{{a}_{{ij}2}}\right)\right\}$$

Alternatively, the variance of the PRS can be estimated via a regression model, foregoing the requirement for independent variants, as explained in Eqs. (18) and (19) of the “Methods” section.

The mean and variance of a PRS (based on either global or local ancestry patterns) can next be used to construct a third metric: the qPRS, which provides the population-level genetic risk “position” of an individual in the form of a percentile which is independent of genetic ancestry. Statistically, we assume that given the mean and variance, the distribution of the PRS for individual $i$ follows the Normal distribution with mean $E({{PRS}}_{i})$ and variance Var$({{PRS}}_{i})$, i.e.,

8 $${{PRS}}_{i}|E\left({{PRS}}_{i}\right),{Var}\left({{PRS}}_{i}\right) \sim N(\mu=E\left({{PRS}}_{i}\right),{\sigma }^{2}={Var}\left({{PRS}}_{i}\right))$$

The qPRS then can be computed as

9 $${{qPRS}}_{i}=\Phi ({{PRS}}_{i};\mu=E\left({{PRS}}_{i}\right),{\sigma }^{2}={Var}\left({{PRS}}_{i}\right))$$

where the notation $\Phi$ is the cumulative Normal distribution function. Thus, the qPRS is a person-specific percentile of PRS value conditional on an individual’s ancestral makeup. The schematic illustration of the ePRS framework as well as example distributions of PRS, ePRS, and rPRS for body mass index (BMI) in the TOPMed dataset are shown in Fig. 1. A high-level summary and comparison of the proposed ePRS framework with the PC-adjusted PRS approach are presented in Table 1. Further details on the ePRS framework and the formula derivations are provided in the “Methods” section.

**Table 1:** Summary and comparison of the proposed ePRS framework with the PC-adjusted PRS approach

Concept/Metric	ePRS framework	PC-adjusted PRS framework	Interpretation
PRS	${{PRS}}_{i}={\sum }_{j=1}^{p}{\omega }_{j}\times {g}_{{ij}},i={{\mathrm{1,2}}},\ldots,n$		Quantitative measure of an individual’s genetic predisposition to a trait. Note: In both frameworks, the PRS can be computed using any existing method, which may differ in variant selection strategies and effect size weighting (${\omega }_{j}$) procedures, such as PRS-CS or LDprd2.
Ancestry background-specific PRS	Expected PRS (ePRS): baseline genetic liability computed conditioned on individual’s ancestry makeup from admixture analysis Global ePRS (gePRS): ${{gePRS}}_{i}={\sum }_{k=1}^{K}{\pi }_{{ik}}\times \left\{{\sum }_{j=1}^{p}{2\times \omega }_{j}\times {f}_{j}^{{a}_{k}}\right\}$ Local ePRS (lePRS): ${{lePRS}}_{i}={\sum }_{j=1}^{p}{\omega }_{j}\times ({f}_{j}^{{a}_{{ij}1}}+{f}_{j}^{{a}_{{ij}2}})$	PC-predicted PRS: compute predicted PRS value from regression of PRS on top genetic PCs: Step1: $E\left({PRS}|{PCs}\right)={\sum }_{j=1}^{S}{\Theta }_{j}\times {{PC}}_{j}$ Step2: ${{PRS}}_{i}^{{pred}}={\sum }_{j=1}^{S}{\hat{\Theta }}_{j}\times {{PC}}_{{ij}}$ Note: Not currently used by existing procedures, beyond for the purpose of obtaining the ancestry-agnostic PRS.	Ancestry-dependent trait-specific genetic value for an individual, predicted from ancestry measures. Ancestry is conceptualized based on pre-specified reference populations in the ePRS framework, and unsupervised in PC-based analysis.
Ancestry-agnostic PRS	Residual PRS (rPRS): An ancestry-adjusted PRS obtained by subtracting an individual’s ancestry background-specific PRS (ePRS) from their observed PRS ${rPR}{S}_{i}={PR}{S}_{i}-{ePR}{S}_{i}$	PC-adjusted PRS: Obtained by subtracting the ancestry-predicted PRS from the observed PRS. PC-adjusted ${{PRS}}_{i}={{PRS}}_{i}-{{PRS}}_{i}^{{pred}}$	Trait-specific adjusted PRS value that is ancestry-agnostic (in terms of baseline genetic liability).
Population-level genetic risk position	Quantile PRS (qPRS): An individual’s relative percentile of genetic liability, given their ancestry background-specific value (ePRS) and estimated variance ${{qPRS}}_{i}=\Phi ({{PRS}}_{i};\mu=E\left({{PRS}}_{i}\right),{\sigma }^{2}={Var}\left({{PRS}}_{i}\right))$	Step 1. The variance of the PRS can be estimated by regressing the squared of PC-adjusted PRS on genetic principal components. Step 2. The corresponding quantile-specific PRS can then be derived using the predicted PRS values together with the predicted variance obtained from Step 1. Note: This procedure has not yet been directly implemented in existing methods.	Relative genetic liability position of an individual’s PRS within an ancestry-specific distribution.
Adjustment in PRS-outcome association model	Working association model: $g\left\{E\left(Y\right)\right\}={\widetilde{\beta }}_{0}+{{{{\boldsymbol{X}}}}}^{T}{\widetilde{{{{\boldsymbol{\delta }}}}}}_{x}+{\widetilde{\beta }}_{1}\times {rPRS}+\varphi \times {ePRS}$	Working association models: (i) $g\left\{E\left(Y\right)\right\}={\widetilde{\beta }}_{0}+{{{{\boldsymbol{X}}}}}^{T}{\widetilde{{{{\boldsymbol{\delta }}}}}}_{x}+{\widetilde{\beta }}_{1}\times {PC}-{adjusted\; PRS}+{\sum }_{j=1}^{S}{\lambda }_{j}\times {{PC}}_{j}$ (ii) $g\left\{E\left(Y\right)\right\}={\widetilde{\beta }}_{0}+{{{{\boldsymbol{X}}}}}^{T}{\widetilde{{{{\boldsymbol{\delta }}}}}}_{x}+{\widetilde{\beta }}_{1}\times {PC}-{adjusted\; PRS}$	Both models aim to remove ancestry-related confounding and adjust for population stratification bias in PRS-outcome association analysis.
Key features	Computed using discrete ancestry categories definition and ancestry-specific allele frequencies; may rely on either global or local ancestry; interpretable as a population-level genetic liability.	Uses genetic PCs, unsupervised, continuous, measures of ancestry; simpler to compute; requires computation of PCs based on pre-defined selection of variant and loadings, or a large dataset to compute PCs.	ePRS: does not require regression analysis; available in analytical form.PC-adjusted: Computationally simpler but less interpretable.
Potential approach for clinical use	Provide ePRS, rPRS, and qPRS as ancestry-specific and individual genetic risk measures, and as population-level risk position (percentile).	Used in current trials: provide PC-adjusted PRS value, provide population-level risk position (percentile) in each of several discrete population categories.	Individuals receiving their PRS measures may also recieve both their ancesty-specific (ePRS) and -agnostic (rPRS/qPRS) measures in the ePRS framework.

### Results from simulation studies

#### Overview of the simulation settings

We conducted extensive simulation studies to examine the estimation performance of PRS-outcome association (${\beta }_{1}$ in Eq. (1)) when using the ePRS framework in comparison with other possible association models. The simulation studies mostly focused on a three-way admixed population, but we also implemented additional simulations on a more complex scenario involving a six-way admixed population. We simulated a few ancestry makeup patterns and used them to generate a PRS and an unobserved confounder ${U}_{G}$. Supplementary Fig. 1 provides an overview of the procedure for generating global and local ancestries as well as the PRS, the unknown genetic confounder ${U}_{G}$, and the outcome. The population structure pattern captured by the first 2 PCs in the simulated genetic data is visualized in Supplementary Fig. 2. To guide the definition of the simulated PRS, we used summary statistics from a GWAS of systolic blood pressure (SBP) of the UK Biobank (UKBB) and ICBP consortium^27^. We used the top 100 SNPs after clumping using PLINK and extracted their estimated effect sizes from the GWAS as the true variant weights. The ancestry-specific frequencies ${f}_{j}^{{\alpha }_{k}}$ of these SNPs were taken to be the estimated ancestry-specific frequencies from the global ancestry-specific allele frequency estimation in admixed populations (GAFA) procedure applied over the TOPMed dataset^28^. Two types of PRSs were considered in our simulations: homogenous weighting PRS, where the variant weights are set to be the same across ancestries, and heterogeneous weighting PRS, where some of the weights are ancestry-specific (see “Methods”). We simulated the unknown confounder ${U}_{G}$ in a few forms (see Table 2 for more details), and it was generally sampled to be ancestry-related using a similar strategy to the PRS. Further, a few forms of ${U}_{G}$ were a weighted sum of alleles, just like a PRS. Thus, similar to the simulation of the main PRS, we used summary statistics from GWAS to guide the selection of SNPs to inform of ancestry-specific allele frequencies and weights. SNPs were sampled from either the UKBB-ICBP SBP GWAS (conf1 (local ancestry)) or from the SBP GWAS of the Million Veteran Program (MVP), in which case, top SNPs that were highly common (MAF > 0.4) in African ancestry and relatively rare (MAF < 0.1) in European ancestry were used (conf2 (afr enriched)). We also used a single variant or a combination of two variants as genetic confounders, again enriching for African frequency. The purpose of simulating alleles that are common in one particular ancestry was to create a strong genetic ancestry confounding effect. For comparison, we also set the “unobserved” confounder to be a known (i.e., observed) genetic principal component (conf-pc*), essentially another weighted sum of SNPs. Notably, in all simulations other than when using a conf-pc* as a confounder (for benchmarking purposes), the association model is always different than the data-generating model. The PRS value considered in each association model (compare with data-generating model) is the homogeneous weighting PRS, which forms a model misspecification scenario when the underlying model is generated by the heterogeneous weighting PRS. We summarized the settings and the rationale for creating different simulation scenarios in Table 2 and Supplementary Note 1. Comprehensive details about the simulation studies are provided in the “Methods” section.

**Table 2:** Simulating the unknown genetic confounding factors

Confounding variable	Sampling of genetic variants	Weighting	Goal
Conf1 (shared local ancestry)	We used ancestry-specific allele frequencies of 100 randomly-selected variants from the UKBB-ICBP SBP GWAS.	Allele weights sampled from a $N({{\mathrm{0,1}}})$ distribution.	Assess and compare the estimation performance of PRS-outcome association between local ePRS and global ePRS.
Conf2 (afr enriched)	We used ancestry-specific allele frequencies of the top 100 SNPs (after pruning) identified in the MVP SBP GWAS, further requiring that the variant have African-specific MAF > 0.4 and European-specific MAF < 0.1.	Allele weights taken from the MVP GWAS (corresponding to the SNPs selected to guide the sampling of alleles).	Generate stronger confounding compared to conf1 (local ancestry); a reasonable scenario whether under-represented genetic ancestry (e.g., African ancestry) has unknown genetic effects from variants that are rare in other ancestries.
Single variant	We used ancestry-specific allele frequencies based on a randomly-selected variant from the top 100 SNPs described in Conf2 (afr enriched).	No weighting.	Assess the effect of strong unknown genetic effect that is easily described.
Two variants	We used ancestry-specific allele frequencies based on a two randomly-selected variants from the top 100 SNPs described in Conf2 (afr enriched).	No weighting (two separate variables).	Assess the effect of strong unknown genetic effect that is easily described.
Conf-pc*	We used ancestry-specific allele frequencies of 100 randomly-selected variants from the MVP SBP GWAS.	Allele weights sampled from a $N({{\mathrm{0,1}}})$ distribution.	Benchmarking.
None	–	–	Compare model misspecification when using homogeneous PRS when the underlying PRS is heterogeneous.

The goal (hypothesis to examine) of each simulation settings are listed in the last column of this table

#### Simulation results: estimation performance of the PRS-outcome association in the ePRS framework

**Fig. 2:**
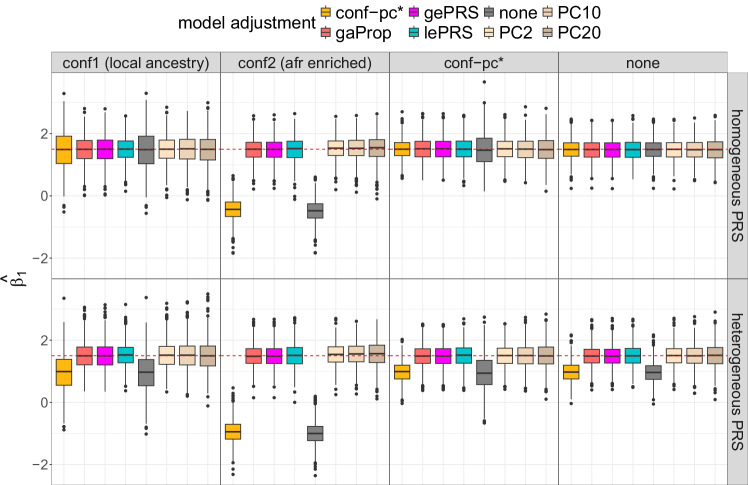
PRS effect size estimates in simulation studies. The figure provides box plots of the distribution of estimated effect sizes of the PRS (${\hat{\beta }}_{1}$) across simulation settings. Each point in the box plot represents an estimated effect size from one of the 1000 simulation replications. The center line of each box represents the median (50th percentile). The lower and upper bounds of the box correspond to the first (25th percentile, Q1) and third (75th percentile, Q3) quartiles, respectively. The whiskers extend to the minimum and maximum values within 1.5× the interquartile range (IQR = Q3$-$Q1) from Q1 and Q3. Observations beyond the whiskers are plotted as individual points and represent outliers. Results on the top row correspond to data-generating model with homogeneous weighting PRS, and results in the bottom row correspond to heterogeneous weighting PRS. Columns correspond to four forms of confounding factors defined in the data-generating model: conf1 (local ancestry), conf2 (afr enriched), conf-pc*, and no confounding (from the left to the right). Each panel provides boxplots visualizing distributions of ${\hat{\beta }}_{1}$ obtained in 8 association analysis models, with the *y*-axis representing ${\hat{\beta }}_{1}$ values. The true value, ${\beta }_{1}=1.5$, is highlighted as a red dotted line. Association analyses that estimate (standard) PRS effect while adjusting to covariates include PC2, PC10, PC20 (adjusting for top 2, 10, and 20 genetic PCs, respectively), none (no covariate adjustment), conf-pc* (adjustment for a known confounder, for benchmarking), and gaProp (adjustment for global proportions of ancestry). Association analyses applying the ePRS framework include gePRS and lePRS (estimation of rRPS effect adjusting for ePRS, based on global and local models, respectively). Distributions are provided from 1000 simulation repetitions. conf confounding, afr African ancestry, ePRS expected PRS, gePRS global ancestry expected PRS, lePRS local ancestry expected PRS, PC principal component, PRS polygenic risk score, gaProp global ancestry proportions.

**Fig. 3:**
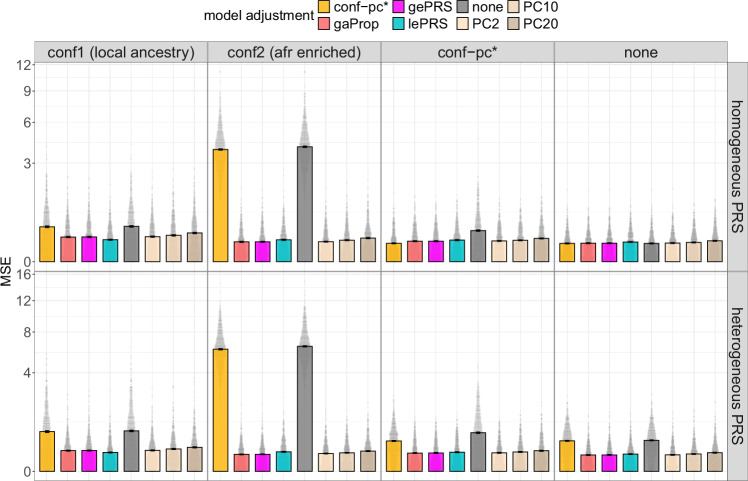
Mean squared error of PRS effect size estimates in simulation studies. The figure provides bar plots visualizing the MSE of estimated effect sizes of the PRS (${\hat{\beta }}_{1}$) across simulation settings. Bar heights represent the MSE, computed as the average squared estimation error over 1000 simulation replicates. Individual points denote squared errors from each replicate. Intervals around the estimated MSE correspond to the MSE +/− one estimated standard error. Results on the top row correspond to data-generating model with homogeneous weighting PRS, and results in the bottom row correspond to heterogeneous weighting PRS. Columns correspond to four forms of confounding factors defined in the data-generating model: conf1 (local ancestry), conf2 (afr enriched), conf-pc*, and no confounding (from the left to the right). Each panel provides bars with heights corresponding to the MSE of ${\hat{\beta }}_{1}$ estimated in 8 association analysis models. Association analyses that estimate (standard) PRS effect while adjusting to covariates include PC2, PC10, PC20 (adjusting for top 2, 10, and 20 genetic PCs, respectively), none (no covariate adjustment), conf-pc* (adjustment for a known confounder, for benchmarking), and gaProp (adjustment for global proportions of ancestry). Association analyses applying the ePRS framework include gePRS and lePRS (estimation of rPRS effect adjusting for ePRS, based on global and local models, respectively). MSE mean square error, conf confounding, afr African ancestry, ePRS expected PRS, gePRS global ancestry expected PRS, lePRS local ancestry expected PRS, PC principal component, PRS polygenic risk score, gaProp global ancestry proportions.

**Fig. 4:**
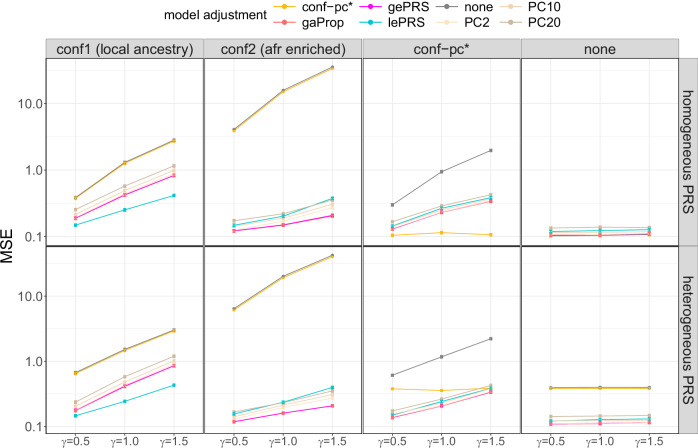
Estimation performance of the PRS effect size across increasing strength of the unknown genetic ancestry-related confounding factor. The figure provides the estimated MSE of the estimated PRS effect size (${\hat{\beta }}_{1}$) across simulation settings and analysis methods. Results on the top row correspond to data-generating model with homogeneous weighting PRS, and results in the bottom row correspond to heterogeneous weighting PRS. Columns correspond to four forms of genetic ancestry-related confounding factors defined in the data-generating model: conf1 (local ancestry), conf2 (afr enriched), conf-pc*, and no confounding (from the left to the right). Each panel provides MSE (y-axis) obtained across 1000 simulation repetitions with association analyses using 8 combinations of PRS and adjustment approaches, and across 3 simulated effect sizes ($\gamma$) of the genetic ancestry-related confounder. In this simulation, we fixed ${\beta }_{1}=1.5$ in the data-generating model across all settings. Association analyses that estimate (standard) PRS effect while adjusting to covariates include PC2, PC10, PC20 (adjusting for top 2, 10, and 20 genetic PCs, respectively), none (no covariate adjustment), pc* (adjustment for a known confounder, for benchmarking), and gaProp (adjustment for global proportions of ancestry). Association analyses applying the ePRS framework include gePRS and lePRS (estimation of rRPS effect adjusting for ePRS, based on global and local models, respectively). MSEs were computed over 1000 simulation repetitions. Intervals around the estimated MSE correspond to the MSE +/− one estimated standard error. MSE mean square error, conf confounding, afr African ancestry, ePRS expected PRS, gePRS global ancestry expected PRS, lePRS local ancestry expected PRS, MSA mean squared error, PC principal component, PRS polygenic risk score, gaProp global ancestry proportions.

Selected results from simulation studies are shown in Figs. 2–4, demonstrating that: (a) the estimated effect size of rPRS when adjusting for either global or lePRS in the model is unbiased for ${\beta }_{1}$ (Fig. 2, all close to true ${\beta }_{1}=0.15$ on average); (b) Under the conf1 (local ancestry) setting, where unobserved genetic confounders share local ancestry intervals with PRS variants, adjustment using lePRS is more efficient than gePRS, with MSE of 0.15 for lePRS, compared to 0.18 for gePRS; (c) setting strong genetic ancestry confounding, conf2 (afr enriched), causes biased estimates and dramatically increases the MSE of the estimated ${\beta }_{1}$ in models that do not adjust for ePRSs (MSE = 4.08 and 6.43 for none; 3.89 and 6.13 for conf-pc*) but has nearly no increase in bias when applying the ePRS framework (0.12 and 0.15 for global and lePRS, respectively); (d) in the setting where the underlying simulated model is homogenous weighting PRS with conf-pc* confounding, the ePRS framework (MSE = 0.13) performed comparably to the benchmark model (conf-pc*, MSE = 0.11) and outperformed other competing approaches (Figs. 2 and 3); (e) compared with models adjusting for PCs derived from the same genetic data used to construct the PRS, the ePRS framework produces estimates with smaller variance (Fig. 2) and lower MSE (Fig. 3), with the best-performing PC model (PC2) having MSEs of 0.12–0.19 across settings, while the ePRS framework achieves MSEs of 0.11–0.15; (f) estimation using the ePRS framework is more robust to PRS model misspecification (heterogeneous weighting PRS setting, Figs. 2 and 3); (g) while increasing the effect size $\gamma$ of the genetic confounder leads to reduced estimation efficiency (i.e., increased MSE), the magnitude of this increase is less severe under the ePRS framework (e.g., MSE increase from 0.15 to 0.83 versus 0.39 to 2.82 from none in conf1 (local ancestry) setting; 0.15 to 0.38 versus 4.08 to 35.45 in conf2 (afr enriched) setting); (h) estimation of the PRS effect while adjusting for proportions of global ancestries as covariates in the model results in similar performance to the ePRS framework with the global gePRS. Full results from these simulations are provided in Supplementary Note 1 and Supplementary Figs. 3–5. A comparison between the parallel ePRS and PC-adjusted PRS measures, as defined in Eqs. (25)–(27) of the “Methods” section, is shown in Supplementary Figs. 6 and 7. While the correlation between the rPRS and PC-adjusted PRS is high, this consistency does not extend to the comparison between ePRS and ancestry-predicted PRS, where the correlation declines as more PCs are included in the regression model.

Supplementary Note 2 provides comprehensive results from secondary simulations. Specifically, we conducted additional simulations: (a) sensitivity analyses to examine the performance of the ePRS approach under the assumption that random error exits in the global or local ancestry inference (Supplementary Fig. 8); (b) simulation studies demonstrating that the estimated effect of the ePRS is related to the strength of the unknown genetic confounding effect (Supplementary Figs. 9 and 10); and finally (c) simulations demonstrating the use of qPRS in binary trait risk classification analyses (Supplementary Fig. 11). Each of the global and local qPRS approaches may outperform the other in some settings. The population structure of the genetic data generated under the six-way admixture pattern is shown in Supplementary Fig. 12. The corresponding simulation results are presented in Supplementary Fig. 13 (PRS-outcome effect estimates), Supplementary Fig. 14 (MSE of PRS-outcome effect estimates), and Supplementary Fig. 15 (performance trends across varying strengths of unknown ancestry-related confounders). Overall, the results are consistent with the primary simulation studies. Sensitivity analyses varying the sample size in the data-generating model, along with comparisons between the ePRS framework and PC-adjustment methods, are presented in Supplementary Fig. 16. Across all sample size settings, the model adjusted for the global ePRS consistently outperforms the PC-adjustment method and demonstrates greater stability than the lePRS.

Supplementary Note 3 describes additional simulations in which we generated admixed genetic data using real haplotypes from the 1000 Genomes Project Phase 3 reference panel. Haplotypes from the three largest superpopulations—European (EUR, *n* = 503), African (AFR, *n* = 661), and East Asian (EAS, *n* = 504)—were used to construct three-way admixed genomes. Individual global ancestry proportions were sampled from a Dirichlet distribution (mean: 48% EUR, 24% AFR, 28% EAS), and local ancestry tract lengths were simulated using a geometric distribution. Genotypes were drawn from reference haplotypes according to the local ancestry state for each individual and variant. To improve computational efficiency, simulations were restricted to a single chromosome, and 1000 variants were randomly sampled for analysis in each simulation replicate. For PRS construction, we randomly selected 1% of variants as causal and assigned per-allele effect sizes from a standard Normal distribution with variance equal to the predefined SNP heritability divided by the number of causal variants, following Márquez-Luna et al.^29^ and Tubbs et al.^30^.

We simulated continuous traits under varying heritability and confounding strength to compare PRS-outcome associations obtained using the ePRS framework with alternative methods, mirroring the main simulation design. We also simulated binary outcomes to assess the risk classification performance of qPRS relative to PC-adjusted PRS standardization approaches. Results are shown in Supplementary Figs. 17 and 18. Across continuous-trait simulations, estimation error decreased with increasing heritability and increased with stronger confounding across all methods, with global ePRS performing comparably to model adjusted for global ancestry proportions and showing greater stability than PC-adjusted approaches, particularly across varying simulation settings. In binary trait analyses, qPRS consistently achieved equal or higher classification accuracy than PC-adjusted qPRS methods, with PC adjustment using larger numbers of PCs performing worst. Notably, when baseline disease prevalence was correlated with individual ancestry proportions, qPRS provided more stable and accurate risk classification than PC-adjusted approaches.

### PRS association analyses in the Trans-Omics for Precision Medicine (TOPMed) dataset

The TOPMed consortium was established by the National Heart, Lung, and Blood Institute to identify genetic risk factors for heart, lung, and sleep disorders and elucidate biological mechanisms behind them^31,32^. This consortium provides aggregated whole-genome sequencing (WGS) data from individuals from multiple parent studies representing genetically (and environmentally) diverse populations. During the first few years of TOPMed, several phenotypes have been harmonized by the then-existing Data Coordinating Center of TOPMed, where the harmonization was across several cohorts with comprehensive phenotyping^32^. We applied the ePRS framework to estimate the associations of a few PRSs with their outcomes in the TOPMed dataset. As described in the “Methods” section, local ancestry was inferred using RFMix^33^, based on the Human Genome Diversity Project (HGDP) reference panel^34^, where HGDP populations were merged into 7 super-populations: Sub-Saharan Africa, Central and South Asia, East Asia, Europe, Native America, Oceania, Middle East, serving as reference ancestries. Global ancestry proportions were computed from the local ancestries. TOPMed individuals were not grouped by genetic ancestry patterns, and we report association analysis results by self-reported race/ethnicity groups and in the combined population. We considered five continuous phenotypes: BMI, diastolic blood pressure (DBP), SBP, high-density lipoprotein (HDL), low-density lipoprotein (LDL), and two binary outcomes: venous thromboembolism (VTE) and obstructive sleep apnea (OSA). Supplementary Table 1 provides information about the GWAS used for PRS derivation. Across traits, up to 49,626 individuals were included in a given analysis, with sample sizes and parent studies of participants varying across traits. Supplementary Table 2 characterizes TOPMed participants in the continuous trait analyses, Supplementary Table 3 provides summary statistics of these continuous traits in the sample. Supplementary Tables 4 and 5 characterize TOPMed participants in the VTE and OSA analyses, respectively. Supplementary Table 6 provides the number of individuals participating in each of the analyses (combined and broken down by harmonized self-reported race/ethnicity).

### Characteristics of PRS, ePRS, and rPRS across TOPMed individuals and traits

**Fig. 5:**
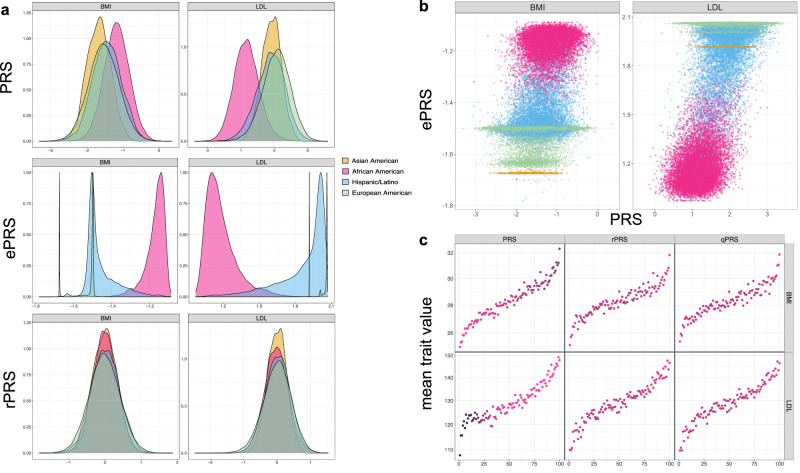
Patterns of BMI and LDL ePRS-related measures in TOPMed participants. **a** BMI and LDL PRS, global ePRS, and global rPRS distributions in TOPMed participants. **b** The relationship between BMI and LDL PRSs and global ePRSs. Each point represents an individual’s global ePRS (*y*-axis) and PRS (*x*-axis) values. **c** Scatterplots visualizing, on the *x*-axis, percentiles of PRS, global rPRS, and global qPRS, against mean values of the corresponding phenotypes (*y*-axis), averaged across individuals with the corresponding PRS, rPRS, or qPRS percentile. For instance, for each phenotype and PRS measure, individuals were binned into 100 strata defined by PRS measure percentiles. The color of a given point corresponds to the proportion of African American individuals among individuals within the relevant percentile stratum. Darker color reflects a higher proportion of African American individuals. Global ePRSs were constructed based on each individual’s global ancestry proportion and the ancestry-specific allele frequency estimated using GAFA. TOPMed Trans-Omics for Precision Medicine, BMI body mass index, LDL low-density lipoprotein, PRS polygenic risk score, ePRS expected PRS, rPRS residual PRS, qPRS quantile PRS.

Figure 5 illustrates the impact of genetic ancestry on BMI and LDL PRSs. Panel a shows that the distribution of conventional PRS differ across self-reported race/ethnicity groups, where African American individuals have higher BMI PRS values and lower LDL PRS values, compared to other groups. This is driven by their ancestral makeup, as demonstrated by the ePRS distributions. In contrast, the rPRS distributions are similar across self-reported race/ethnicity groups and are centered around zero. Panel b provides another view of the ePRS and PRS relationship. For example, it demonstrates that the highly admixed groups, such as African American and Hispanic/Latino individuals, have large variation in ePRS values, while genomes of most Asian and European individuals have little admixture. Panel c visualizes PRS values against trait values (averaged within bins defined by PRS percentiles), with the proportions of African American individuals highlighted in each bin. The figure shows that, under the conventional PRS, African American individuals are disproportionately represented in bins corresponding to high BMI and low LDL values, a distribution that could systematically misclassify them as high or low risk relative to other populations. In contrast, the rPRS and the qPRS appropriately alleviate this issue. Supplementary Figs. 19–25 provide similar visualizations for other traits (SBP, DBP, HDL, OSA, and VTE).

#### Estimation of PRS-outcome associations

We apply the ePRS framework to estimate PRS-outcome associations and compare the results to those from association models using conventional PRS and adjusting for genetic PCs or the estimated global ancestry proportions. We adjusted for 11 genetic PCs in the standard PRS model (except the VTE analysis, in which we adjusted 7 genetic PCs according to the previously published paper^35^). When using the ePRS framework, we estimated the association of the rPRS while adjusting for the corresponding ePRS (global or local), computed with ancestry-specific allele frequencies calculated by GAFA.

**Fig. 6:**
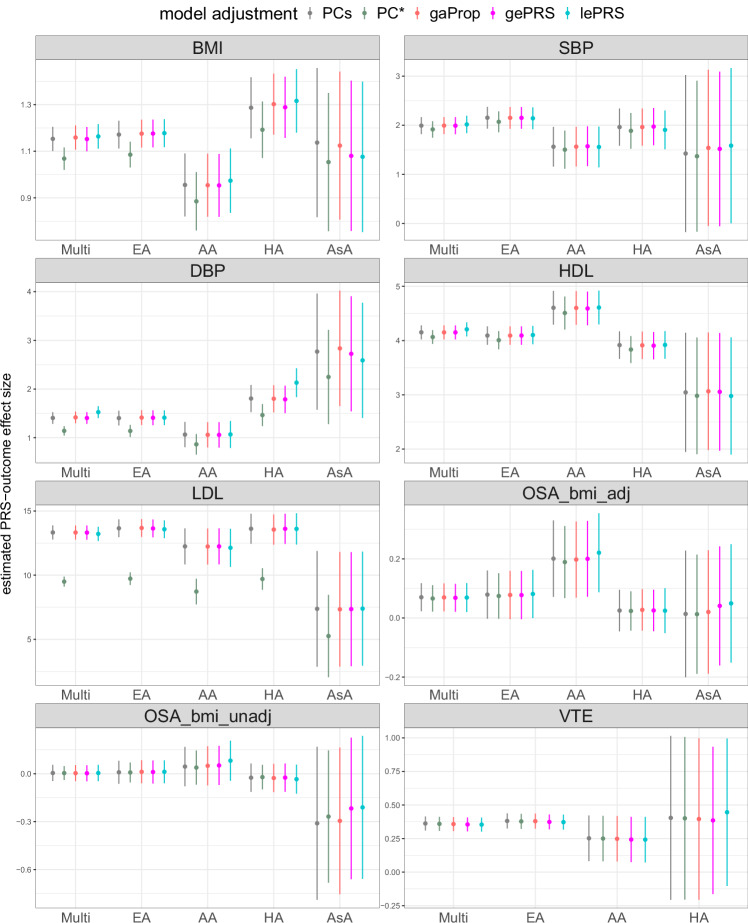
Estimated PRS-outcome association effect sizes for TOPMed studies traits. The figure presents the estimated PRS-outcome effect sizes (*y*-axis; center of the error bars) along with their corresponding 95% confidence intervals in the TOPMed dataset. Conventional PRS model estimated the PRS effect size by either adjusting for genetic PCs or for global ancestry proportions. We additionally include results from PC-adjusted PRS (denoted as PC*), in which the PRS was first adjusted for PCs and then further adjusted PCs as covariates in the association model, for comparison. For the ePRS model, we show the estimated rPRS effect size either adjusting for global or local ePRS. Estimated association are provided, for each trait, for the combined dataset (Multi), and stratified by self-reported race/ethnicity. For continuous traits (BMI, SBP, DBP, HDL, and LDL), effect sizes are in the original trait scale (kg/m2, mmHg, mmol/L). OSA and VTE are binary traits, and their estimated effect sizes are in the log odds ratio scale. For OSA, we provide results for two OSAs: OSA_bmi_adj and OSA_bmi_unadj, based on GWAS that did and did not adjust for BMI, respectively. Due to the limited sample size, we did not perform an Asian-specific analysis of VTE. More details of the analytic approaches for all the analyses can be found in the “Methods” section. The sample sizes used to conduct the analysis and generate the figure are summarized in Supplementary Data 1. BMI body mass index, SBP systolic blood pressure, DBP diastolic blood pressure, HDL high-density lipoprotein, LDL low-density lipoprotein, OSA obstructive sleep apnea, VTE venous thromboembolism, Multi Multi-ethnic, EA European American, AA African American, HA Hispanic/Latino, AsA Asian American, PRS polygenic risk score, PC principal component, gaProp global ancestry proportions, gePRS global ancestry expected PRS, lePRS local ancestry expected PRS.

Figure 6 shows the estimated effect sizes of the PRSs and of the rPRSs with their 95% confidence intervals. Results are provided from multi-population analysis and stratified by self-reported race/ethnicity groups. The results from the multi-population analyses show that all four models had similar estimates, matching what we observed in simulation studies. In some instances, the lePRS approach has slightly larger effect size estimates, most prominently for DBP, where the point estimate is 2.13 (95% CI: 1.85, 2.43), compared to estimates from other methods, which were centered around 1.80 (95% CI: 1.51, 2.07). The estimates obtained via multi-population analyses are close to those from the European American population, as it usually dominates other populations in sample size contribution. The estimates corresponding to the Asian American group have large standard errors due to small sample sizes. Generally, effect estimates often differ across race/ethnic groups. For example, among Asian American, the estimated effects for LDL and HDL PRSs on their traits are substantially lower (point estimates of approximately 7.35 for LDL and 3.05 for HDL), relative to other groups where corresponding estimates were around 12.0 and 4.5, respectively. The estimated effect size for the African American group is higher than others for OSA (BMI adjusted) and HDL; however, the estimated effect sizes are lower than other three populations for most of the other traits. All association analysis results, including the estimated effect sizes, standard error, sample size used for each trait, are summarized in Supplementary Data 1.

For comparison, we also considered a PC-adjusted (residualized) PRS, where the PRS was first regressed on all genetic PCs, and the PRS-outcome association was then estimated using the PC-adjusted PRS, with additional adjustment for all genetic PCs as covariates. For continuous (but not binary) traits, the PRS-outcome effect estimates are theoretically identical whether using the unadjusted PRS or the PC-adjusted PRS, provided that all PCs are included as covariates in the association model, as implied by the Frisch–Waugh–Lovell theorem^36,37^. The differences in effect estimates observed in Fig. 6 are because the PC-adjusted PRS was standardized after regression over PCs (standardization was applied to facilitate consistent interpretation of effect sizes per one standard deviation increase in PRS). This standardization leads to differences in the estimated point estimates and standard errors; however, the corresponding p-values remain identical between the two models.

In the secondary analysis, we developed genome-wide PRSs using LDpred2^38^ and constructed PRS, ePRS, rPRS, and qPRS for each trait. The ancestry-specific distributions of LDpred2 PRS, ePRS, and rPRS are shown in Supplementary Figs. 26–28. Here, the qPRS was computed using PRS variance estimated in a regression over ancestry proportions, because the variants used in the PRS are not independent of each other. The estimated PRS-outcome associations across all traits are similar between the ePRS framework and conventional association models (adjusting for genetic PCs or global ancestry proportions), with the same conclusion as the primary analyses. The PRS-outcome association results are shown in Supplementary Fig. 29. The association between percentiles of PRS, rPRS, and qPRS and the corresponding traits are shown in Supplementary Figs. 30 and 31. The qPRS distribution is well-calibrating, demonstrating that ancestry proportions are not correlated with the qPRS values. Details of secondary data analysis are summarized in Supplementary Note 5 and Supplementary Data 2.

### Analysis of CVD-related traits in All of Us (AoU) research program

We applied the proposed ePRS framework to the AoU dataset using publicly-available resources. This analysis focused on six CVD-related phenotypes: atrial fibrillation (AF), coronary artery disease (CAD), chronic kidney disease (CKD), heart failure (HF), hypertension (HTN), and type 2 diabetes mellitus (T2DM), which are all binary outcomes. We used short-read whole-genome sequencing (srWGS) genetic data (version 7), restricting the analysis to variants with a population-specific allele frequency (AF) $\ge$ 1% or a population-specific allele count (AC) > 100. Variants and weights for PRS construction were obtained from the PGS Catalog. For computing ePRS, we used ancestry-specific allele frequencies from gnomAD (version 3.1.2), which align with the ancestry definitions in AoU. This analysis focused on computing global ePRS, which was derived using the global ancestry proportions estimated for each individual in the AoU Research Program. The characteristics of AoU participants and the selected phenotypes are summarized in Supplementary Tables 7 and 8, while details of the PGS (PGS catalog IDs, number of variants) used for PRS computation are provided in Supplementary Table 1. Additional details on the AoU analysis are available in the “Methods” section and Supplementary Note 6. For models adjusting for genetic PCs, we adjusted for the full set of 16 genetic PCs provided by the AoU genomic data release to control for population structure.

**Fig. 7:**
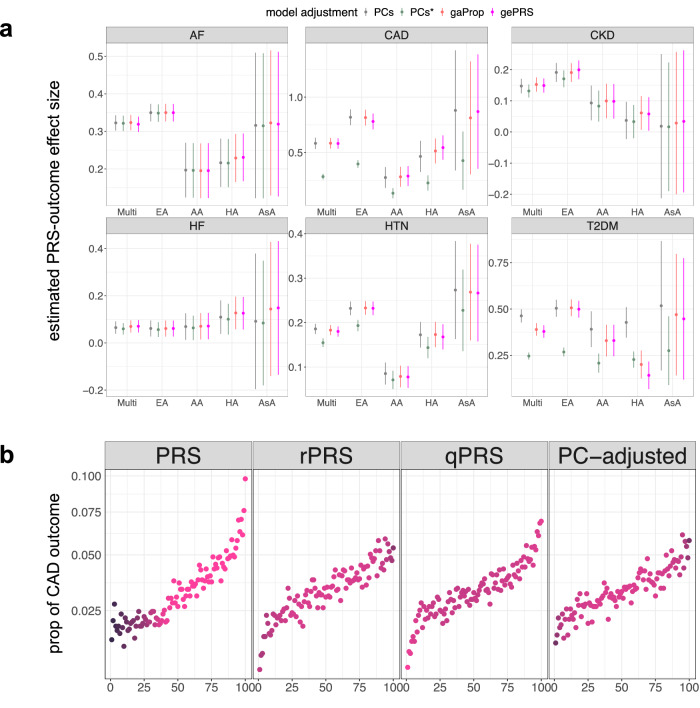
Estimated PRS-outcome effect sizes in AoU analysis. Results from association analysis in AoU. **a** Estimated PRS-outcome effect sizes (*y*-axis) and their corresponding 95% confidence intervals in the AoU dataset. The center of each error bar represents the point estimate of the PRS-outcome effect size. The conventional PRS model estimated the PRS effect size by adjusting either for genetic PCs or for global ancestry proportions. For the ePRS model, we report the estimated rPRS effect size, adjusted for global ePRS. We also include the effect size of PC-adjusted PRS (denoted as PC*), further adjusted for PCs in the association analysis, for comparison. Estimates are provided for each trait in the combined dataset (labeled “Multi”) and stratified by self-reported race/ethnicity. Effect sizes are reported on the log odds ratio scale. **b** Proportions of AoU individuals with CAD for groups defined by percentiles of each PRS measure. PRS: raw PRS. rPRS and qPRS were based on global ePRS. PC-adjusted is the residual from regression PCs over genetic PCs. Further details on the analytical approaches used in all analyses can be found in the “Methods” section. The sample sizes used to conduct the analysis and generate the figure (**a**) is summarized in Supplementary Table 8. AF atrial fibrillation, CAD coronary artery disease, CKD chronic kidney disease, HF heart failure, HTN hypertension, T2DM type 2 diabetes mellitus, Multi Multi-ethnic, EA European American, AA African American, HA Hispanic/Latino, AsA Asian American, PRS polygenic risk score, PC principal component gaProp global ancestry proportions, gePRS global ancestry expected PRS.

Figure 7 summarizes the results. As visualized in panel a, we compared the estimated PRS-outcome associations obtained using the proposed approach, which adjusts for global ePRS, with the estimates derived from models either adjusting for genetic PCs or global ancestry proportions. We also included a method that uses PC-adjusted PRS (denoted as PC* in the figure) as covariate instead of PRS, following the procedure described in Khera et al.^2^, for comparison. Overall, the PRS-outcome association estimates were consistent between the models adjusted for global ePRS and global ancestry proportions and, in most cases, were similar to those from models adjusted for PCs. However, using PC-adjusted PRS as the PRS measure led to underestimation of the PRS effect size estimates, particularly in the analyses of CAD and T2DM. For instance, among the European American (White) individuals, the odds ratio (OR) per 1 standard deviation (SD) increase in CAD PRS is 2.18 (95% CI: 2.03, 2.34) using the ePRS model, compared to 1.48 (95% CI: 1.43, 1.53) using PC-adjusted PRS model. Similarly, for T2DM analysis, the estimated OR is 1.65 (95% CI: 1.57, 1.72) using ePRS model, versus 1.30 (95% CI: 1.27, 1.33) from PC-adjusted PRS model. This underestimation is because the SD of the PC-adjusted PRS was sometimes very different than the SD of the PRS (prior to its regression on PCs), leading to these differences in effect sizes measured per 1 SD of the PRS measure. Panel b of Fig. 7 visualizes the proportion of individuals with CAD within each PRS percentile, comparing the PRS itself, the rPRS, qPRS, and the PC-adjusted PRS. The color of each point corresponds to the proportion of individuals from the Black race/ethnicity group, demonstrating that the PRS is unbalanced in that low PRS percentiles are enriched in Black individuals. All other polygenic risk measures are well balanced in this respect. The qPRS demonstrates an appropriate pattern of CAD proportions across groups defined by its percentiles. All association analysis results, including the estimated effect sizes, standard error, sample size used for each trait, are summarized in Supplementary Data 3.

**Fig. 8:**
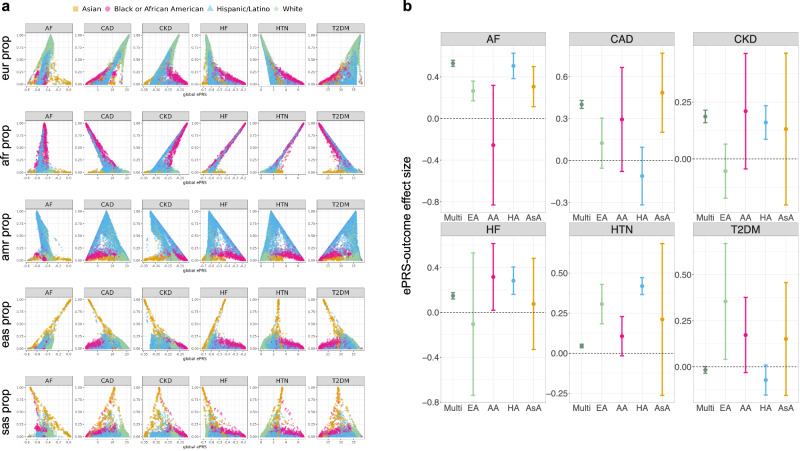
Associations of ePRS with global ancestry and with outcomes in AoU. **a** visualizes the relationship between global ePRS (*x*-axis) and ancestry proportions (*y*-axis), stratified by self-reported race/ethnicity. Global ancestry proportions are shown across five reference populations reported by AoU. Each point represents an individual, colored by self-reported race/ethnicity. **b** shows the estimated effects of global ePRS-outcomes association (*y*-axis) with corresponding 95% confidence intervals. The center of each error bar represents the point estimate of the global ePRS-outcome effect size. Estimates are adjusted for rPRS and reported for each trait in the combined dataset (“Multi”) and stratified by self-reported race/ethnicity. Effect sizes are on the log odds ratio scale. The sample sizes used to conduct the analysis and generate the figure (**b**) is summarized in Supplementary Table 8. AoU All of Us, ePRS expected PRS, rPRS residual PRS, PRS polygenic risk score, AF atrial fibrillation, CAD coronary artery disease, CKD chronic kidney disease, HF heart failure, HTN hypertension, T2DM type 2 diabetes mellitus, eur European, afr African American, amr American Admixed/Latino, eas East Asian, sas South Asian, prop ancestry proportion, Multi Multi-ethnic, EA European American, AA African American, HA Hispanic/Latino, AsA Asian American.

To further evaluate the ePRS, we examined ePRS associations with global ancestry proportions and estimate ePRS-outcome associations, as summarized in Fig. 8. The scatterplots (Fig. 8a) show strong associations between the ePRS and African ancestry proportions in Hispanics/Latinos and African American individuals. For example, the ePRSs of both HF and HTN had Spearman correlation >0.99 with proportion of African ancestry in both groups. In contrast, ePRSs of CAD and T2DM had strong negative correlations with African ancestry proportions. The ePRS effect size estimates in the association models (with rPRS included) were statistically significant across all outcomes when considering all individuals together (“multi” results in Fig. 8b). In stratified analyses, the associations were strongest in Hispanics/Latinos, with statistically significant effects observed for AF, CKD, HF, and HTN, likely reflecting the strong admixture in this population. Among African Americans, a statistically significant association of the ePRS was detected for HF. Taken together, the strong ePRS-HF association and the high correlation between the HF ePRS and African ancestry proportion parallels the findings from our simulation studies (Supplementary Figs. 9 and 10), where ePRS effect estimates are increased with stronger unknown trait-related ancestry-correlated components. These results suggest the presence of an uncharacterized genetic component of HF enriched in African ancestry, underscoring the additional insights provided by ePRS beyond the conventional PC-adjusted framework.

The characteristic of the PRSs, ePRSs, rPRSs, and qPRSs across AoU individuals and the considered traits are summarized in Supplementary Figs. 32–36. Correlations between the ePRS and parallel measures obtained from the PC-adjusted framework are presented in Supplementary Tables 9 and 10. The correlation between rPRS and PC-adjusted PRS is consistently high across all traits, though correlations are lower in the highly admixed population of Hispanic/Latino individuals. In contrast, correlations are lower between the ePRS and ancestry-predicted PRS. Sensitivity analyses examining different numbers of genetic PCs in the PC-adjusted PRS framework are summarized in Supplementary Fig. 37.

## Discussion

We proposed an individual-level metric, the ePRS, calculated according to one’s ancestral makeup, to provide an equitable way to quantify genetic risk across diverse populations. We show that the ePRS framework addresses three issues: first, it is used to generate the rPRS, representing the individual variation from the expected mean PRS value according to the individual’s ancestral makeup, similar to the PC-adjusted PRS; second, the ePRS can be effectively used as an adjusting covariate in association model, without further including PCs, to control for stratification bias; three, the qPRS, a measure that further accounts for an individual’s PRS variance, is used to describe the quantile of the individual’s PRS value given their distribution of potential PRS values, where this distribution is based on their ancestral makeup. While the ePRS framework does not make up for missing information about trait-associated variants and their effect sizes in various populations due to underrepresentation in GWAS, it improves PRS implementation in diverse populations. The ePRS has a clear interpretation as the baseline genetic characteristic of an individual for a given trait, according to their specific ancestry composition. PCs, in comparison, are more general and do not have trait-specific meaning. The interpretation of the ePRS is a useful feature of an approach that only relies on ePRS, rPRS, and perhaps qPRS.

The ePRS is proposed to calibrate the PRS to obtain the rPRS and qPRS, which do not depend on ancestry. Another approach that has been used to “remove” the effect of ancestry on PRS is the “ancestry-adjusted PRS” method, where PCs are regressed out of the PRS via a linear model, and then the PRS-outcome association is estimated with the residuals from this regression used in lieu of the PRS^2,10,11^. This approach is computationally simple and is already implemented in widely used open-source software^12^. Khera et al.^2^ showed that the ancestry-adjusted PRS had similar distributions across diverse populations, similarly to what we see in the distributions of rPRS in data analysis. Indeed, there are some similarities between the two frameworks of PC-adjusted PRS and the ePRS. The ePRS estimates the expected value of a PRS based on admixture patterns. The admixture patterns are estimated based on discrete populations and allele frequencies for these populations are extracted from reference panels. In contrast, as we show in Eqs. (25) and (26), the PC-adjusted framework can also be viewed as estimating an expected value of a PRS. In the existing framework, this is a “nuisance” parameter, which has not been of interest. And yet, the predicted value of the PRS obtained from regression on PCs, i.e., the “ancestry-predicted PRS”, resembles the ePRS. In this context, the PC-adjusted PRS is parallelized to the rPRS: both are residuals obtained from subtracting an estimated expected value of a PRS from its observed value. Nevertheless, the distinction between how these expected values is obtained is important. The ePRS in the current framework relies on a discrete definition of ancestry populations: ancestral populations are pre-defined, and their proportions—continuous measures—are estimated for each individual in the data. The ancestry/PC-predicted PRS (that currently isn’t being used) relies on unsupervised measures of ancestry—PCs. The ePRS requires estimation of proportions of ancestry and ancestry-specific allele frequencies, which are easily obtained. The ePRS can then be constructed regardless of the PRS variants and weights. In contrast, the ancestry-predicted PRS requires a regression model where a given PRS is regressed of PCs to estimate the PC coefficients, and having similar PCs (based on the same variants and weights) across datasets used. In practice, we compared the rPRS to the PC-adjusted PRS, as well as the ePRS to the ancestry-predicted PRSs, and, while correlated, they are not the same. Another difference is in potential communication of the estimated PRS. Within the ePRS framework, it can be explained as relating to an individual’s pedigree, in contrast, the ancestry-predicted PRS does not have such an interpretation. Furthermore, standard PCs do not capture local ancestry patterns that may be specific to the PRS. Finally, because PC derivation is not model-based and is unsupervised, the PC adjustment approach does not require definitions of ancestral populations, and it offers the potential for improved modeling of PRS variance, as implemented the PGS catalog via regression of squared residuals (i.e., the squared values of the PC-adjusted PRS) over PCs. Because the ePRS framework also uses continuous ancestry proportions, we also implemented a data-driven approach to compute PRS variance, mimicking the PRS variance approach used in the PGS catalog with PCs. This approach can be used in lieu of our model-based approach, for instance, when the variants used in the PRS are not independent. Our data analysis demonstrated that this approach results in a well-calibrated qPRS measure.

When referring to the ePRS framework as “equitable”, we mean that an individual of a specific genetic ancestry will not be automatically annotated has having “high” or “low” risk when their genetic ancestry implies that their PRS values are expected to be higher or lower than that of other individuals. The ePRS framework conceptualizes personalized PRS distributions based on one’s genetic ancestry makeup. Observed PRS values are thus contextualized based on an individual’s ancestry composition, which determines the potential attained PRS values. Consequently, “high” or “low” PRS values are determined in relation to this potential, allowing the same PRS could be useful independently of population descriptors such as genetic ancestry, or race and ethnicity (which are noisy correlates of genetic ancestry and have no biological meaning). By its definition, the ePRS can be considered as the baseline genetic characteristic of an individual according to their specific ancestry composition. We applied the ePRS framework to study PRS associations with 7 phenotypes in the TOPMed dataset and 6 outcomes in the AoU dataset. Across phenotypes, the estimated effect sizes of the rPRS adjusting for the ePRS are similar to the estimated effects of the PRS adjusting for genetic PCs or global ancestry proportion. Further, in comparison with the PRS, qPRS values are not biased by genetic ancestry (where extreme values of the PRS are dominated by a specific genetic ancestry). Ideally, our recommendation is to use the rPRS for association or clinical prediction models, and to use the qPRS when communicating genetic risk based on PRS only, i.e., as percentile ranks^39^. Recently, the GenoVA study reported PRS risk to participants based on values of the PRS after regression on genetic PCs^8^. Use of the qPRS would mimic this strategy. That said, the qPRS may not always be appropriate due to limitations in computing the variance of its distribution within an individual, depending on the variants used.

Our proposed method has a few notable strengths. First, ePRS accounts for population stratification in a similar fashion to genetic PCs; however, because the ePRS is constructed such that it is specific to the PRS of interest, it has more intuitive interpretation. Further, the ePRS represents the same quantity across datasets, in contrast to PCs, which are typically constructed independently in each dataset (though some approaches have been proposed for unifying PCs across datasets, e.g., Naret et al.^40^, or projecting to the PC space using reference panel^12^). While these authors also showed that PCs are used for prediction models when aligned across datasets, this alignment procedure requires joint quality control across multiple datasets, which is difficult, and critically, the ePRS framework is not inconsistent with this approach, because PCs can be incorporated to prediction models that include the ePRS. Second, although approaches such as PC-adjusted PRS have been proposed for clinical application to address ancestry-related heterogeneity in PRS distributions, the ePRS framework finally integrates all these concepts, namely, rPRS and qPRS, while also providing an additional and interpretable measure of baseline genetic risk, as captured by ePRS, within a single unified model. Lastly, the ePRS framework may result in reduced use of race and ethnicity, which are merely poor proxies of relevant genetic information, in clinical use of PRS. While race/ethnic classifications correlate with genetic ancestry (e.g., because self-reported White individuals usually have large proportions of European genetic ancestries, etc.), two individuals self-identifying with the same race/ethnicity group may have different mixes of genetic ancestry proportions^41,42^. Therefore, the distributions of PRS may arbitrarily differ across groups, potentially leading to wrong interpretations when stratifying individuals into risk groups. In contrast, genetic ancestry is defined or estimated by admixture pattern, which is a more accurate characteristic than the ambiguous definition of self-reported race ethnicity^43^. How to explicitly use race/ethnicity self-reported information in biomedical or genetics research still debated, given that it is often unclear what such variables measure^42^. For that reason, we expect our ePRS framework to be widely adopted and refined in order to limit potential use of self-reported race/ethnicity in clinical use of PRS while still providing accurate, well-defined measures of genetic risk.

A major consideration and limitation of the proposed ePRS framework is the computational burden and estimation accuracy of the global/local ancestry as well as the ancestry-specific allele frequency. We conducted simulation studies to examine the sensitivity of the ePRS to accuracy of both global and local ancestry inference, and showed the robustness of both global and lePRS. Still, the performance of the ePRS framework must be influenced by the accuracy of estimated global/local ancestry pattern. In our simulation studies, although lePRS utilizes more information than global ePRS, and, if accurate, should theoretically perform better than global ePRS, there were settings in which global ePRS outperformed the lePRSs. Many algorithms have been proposed to conduct local ancestry inference in the past few years, including LAMP^44^ and RFMix^33,45^. Among these approaches, RFMix, the approach used to estimate global/local ancestry patterns in the TOPMed dataset, showed high accuracy in estimating ancestry^46,47^. We expect a more accurate and efficient algorithm to be proposed to conduct local ancestry inference and, ultimately, incorporate it into the ePRS framework to increase the estimation performance. Global ancestry estimation is less computationally intensive, making the global ePRS more attractive. The gold standard software for estimating global ancestry proportions is ADMIXTURE^48^. Many additional software have been developed to enable estimation of ancestry proportions in large scale datasets, and to utilize existing large datasets for supervised inference^49,50^.

A question raised by both the PC adjustment approach to the PRS and by the ePRS approach, is whether important information is lost when calibrating a PRS according to genetic ancestry. For example, there could be trait-associated variants that are very common in one ancestry and very rare in other ancestries. Will ancestry adjustment methods inappropriately dismiss the impact of such variants? This issue could be addressed by inclusion of specific variants of strong effect separately in the association model (not within a PRS), akin to use of *APOE* (Alzheimer’s disease^51^) and *APOL1* (kidney disease^52^) alleles. In addition, it may be worth studying and understanding the association of the ePRS itself with the outcome. Compared to the PCs-adjustment method, the ePRS is currently limited in that it is harder to use given the computational complexity and the choices that have to be made regarding ancestry inference. Global ePRS is very easy to compute, it only requires the computation of ancestry-specific ePRS, followed by weighting according to individual’s ancestry proportion. Still, this requires choosing the level of ancestry to use (e.g., continental ancestry? more refined ancestry levels?), and inference of that ancestry, while PC-adjustment does not. While the rPRS does not account for the variance of the PRS distribution according to genetic makeup, the qPRS does. However, currently, we did not account for linkage disequilibrium between variants in the computation of the variance, and therefore, in primary analysis, we only used PRSs that rely on a limited number of highly significant, independent, trait loci. It is an important extension of the ePRS framework to account for LD when computing individual PRS distribution. This will allow for computing qPRS for a PRS that is constructed based on hundreds of thousands or millions of variants. The challenge is mostly computational, and solutions may be adapted from the PC adjustment framework. Finally, an advantage of the ePRS approach is that it requires less individual-level data. For example, and ePRS can be computed for an individual without first developing a regression model of a PRS on PCs. Consequently, it can be computed without having PCs, and without having genome-wide genotyping—as long as ancestry proportions can be computed, e.g., based on a limited set of ancestry-informative markers.

A limitation of our framework is that it uses reference populations to defined ancestry. While this is very much a standard, an important direction of current research encourages the expansion of methodology to the realm of continuous ancestry^53^. When using reference populations to define ancestry, allele frequencies need to be available for the same reference populations as the ones used to quantify ancestry in the data in which ePRS is to be computed. In TOPMed, we used continental ancestries as reference populations, for local ancestry inferences, which was then averaged to provide global ancestry fractions. We computed ancestry-specific allele frequencies using the TOPMed WGS data. AoU used a different method to compute genetic ancestry fractions, and the reference populations are a bit different as Admixed Latinos are included in one category with Native American individuals^54^. The AoU categories match those of gnomAD, which we used for ancestry-specific allele frequencies in ePRS computation. In all, this demonstrates that our method can be applied with existing PRS “instructions” (defined by variants and weights, independent of method used to develop them), and using available frequencies and global ancestry proportions provided by AoU. Importantly, the results in AoU are consistent with those from TOPMed, where the ePRS framework prevents population stratification bias in effect estimates in association analyses, with no need for further adjustment with genetic PCs. Another computational limitation of this study is the use of 100 independent variants in simulations. While this design has been useful in order to directly simulate admixture and local ancestry patterns, we acknowledge that it does not fully capture the LD structure of genome-wide PRS analyses in practice. Still, we think that our simulation studies, coupled with data analysis including using genome-wide PRSs developed with LDpred2, are appropriate to demonstrate the goal and utility of the proposed method in decomposing ancestry-specific and ancestry-agnostic components to improve estimation, risk stratification, and interpretation.

In this work, we use an individual-specific PRS distribution, assuming the variant effect sizes are fixed, admixture patterns are known, and the source of randomness is the variant frequencies. Other publications studied uncertainty in PRS quantifications by incorporating uncertainty in estimating variant weights. For example, Ding et al.^55^ used a Bayesian framework to account for the potential distribution of variant weights. An exciting potential extension of this work is incorporating genetic ancestry information to simultaneously update the ancestry-specific effect size and the admixed pattern of genetics composition. It is a promising future direction for providing comprehensive PRS analysis results.

For the additional simulations using real genotype data to simulate admixed genomes, we focused on a single chromosome to reduce computational burden. We acknowledge that the simulation settings (e.g., number of causal variants, heritability, and effect sizes) may not fully reflect real-world whole-genome architectures. Nevertheless, the comparison between the ePRS framework and the PC-adjusted approach remains fair, as both methods were evaluated on the same simulated datasets. Any potential differences are primarily driven by the underlying assumptions regarding causal variant selection and effect size estimation, which are not the main focus of this study. Extending these evaluations to full genome-wide simulations will be an important direction for future work to further validate the framework under more realistic genetic architectures. Furthermore, it will be important to study the application of the ePRS and the PC-based ancestry adjustment approaches using more comprehensive simulation studies of admixed populations, e.g., by applying models for heritability in admixed populations^56–58^ and simulating potentially complex genetic effects including multiple variants located in a single association region with different LD patterns in ancestral populations.

In summary, the proposed ePRS approach provides a strategy to differentiate individual genetic risk from differences in PRS distributions due to ancestral makeup, computed based on reference populations. The ePRS improves over PCs in its interpretation and transferability across datasets while protecting from population stratification bias in association analysis.

## Methods

### Ethics

All human participants provided written informed consent. The ethics statements and acknowledgements for the AoU Research Program and its participants are detailed in Supplementary Note 6, and those for the contributing studies in TOPMed are provided in Supplementary Note 7. The study design and conduct complied with all relevant regulations regarding the use of human study participants and was conducted in accordance with the criteria set by the Declaration of Helsinki. This study was approved by the Beth Israel Deaconess Medical Center Committee on Clinical Investigations, protocol #2023P000279, and by the Mass General Brigham IRB, protocol #2021P001928.

### Computing expected PRS (ePRS)

The polygenic risk score (PRS) of an individual $i,i={{\mathrm{1,2}}},\ldots,n$, is calculated by the weighted sum of alleles, with weights being the alleles’ estimated effect sizes. This can be written as ${{PRS}}_{i}={\sum }_{j=1}^{p}{\omega }_{j}\times {g}_{{ij}}$. We use the clumping and thresholding method, e.g., as implemented in the PRSice software^59^, in order to have a set of independent genetic variants, to enable efficient computation of the PRS variance, conditional on ancestry, as later described. Here, we treat the weighting parameter ${\omega }_{j}$ as a fixed and known value. We define the expected PRS (ePRS) for an individual $i$ as

10 $${{ePRS}}_{i}=E({{PRS}}_{i})$$

where the $E(.)$ is the expectation, over the random sample of alleles. By the definition of PRS and the calculation of expectation, we can write the ePRS in the following form,

11 $$\begin{array}{c}{{ePRS}}_{i}=E\left({{PRS}}_{i}\right)\\=E\left(\mathop{\sum }_{j=1}^{p}{\omega }_{j}\times {g}_{{ij}}\right)\\={\sum }_{j=1}^{p}{\omega }_{j}\times E({g}_{{ij}})\end{array}$$

In Eq. (11), we assume that ${g}_{{ij}}$ is a random variable and the estimated effect size ${\omega }_{j}$ is fixed value. When focusing on a homogenous population, one can assume the genetic variant follows a binomial distribution ${g}_{{ij}} \sim {Bin}(2,{f}_{j})$, where ${f}_{j}$ is the allele frequency of the allele ${g}_{j}$. Therefore, the expected value of the variant $j$ can be computed as $E({g}_{{ij}})=2\times {f}_{j}$ and the resulting ePRS is $2\times {\sum }_{j=1}^{p}({\omega }_{j}\times {f}_{j})$ for individual $i$. In addition, by assuming that the $p$ variants are independent, we can apply the Binomial model to compute the variance of the PRS of individuals $i$ as

12 $${Var}\left({{PRS}}_{i}\right) 	={Var}\left({\sum }_{j=1}^{p}{\omega }_{j}\times {g}_{{ij}}\right)\\ 	=\mathop{\sum }_{j=1}^{p}{\left({\omega }_{j}\right)}^{2}\times {Var}\left({g}_{{ij}}\right)\\ 	={\sum }_{j=1}^{p}\left[{\omega }_{j}^{2}\times 2\times {f}_{j}\times (1-{f}_{j})\right]$$

We define the rPRS for individual *i* as the difference between ${{PRS}}_{i}$ and the corresponding ${{ePRS}}_{i}$, which is

13 $${{rPRS}}_{i}={{PRS}}_{i}-{{ePRS}}_{i}$$

The rPRS is an index indicating the additive deviation of the PRS value from the underlying ePRS value. In a homogenous population, each individual has the same ePRS value, while they likely have different rPRS values.

Consider the “mixed population” case, in which we assume the genome for each individual is inherited from one ancestry. Assume that in the studied population there are $K$ ancestries ${a}_{i}\in \{{a}_{1},{a}_{2},\ldots,{a}_{K}\}$, and the allele frequency for each genetic variant $j$ in ancestry $k$ is ${f}_{j}^{{a}_{k}}$, $j={{\mathrm{1,2}}},\ldots,{p;k}={{\mathrm{1,2}}},\ldots,K$. The ePRS for each individual conditioned on their ancestry can be computed as

14 $$\begin{array}{c}E\left({{PRS}}_{i} | {a}_{i}={a}_{k}\right)={\sum }_{j=1}^{p}{\omega }_{j}\times E\left({g}_{{ij}} | {a}_{i}={a}_{k}\right)\\=2\times {\sum }_{j=1}^{p}({\omega }_{j}\times {f}_{j}^{{a}_{k}}).\end{array}$$

By Eq. (14), the ePRS for two individuals from the same ancestry is identical. Extending the idea of modeling mixed populations to admixed populations, in which the genome consists of mosaic segments inherited from different genetic ancestries, the Binomial distribution ${g}_{{ij}} \sim {Bin}(2,{f}_{j}^{{a}_{k}})$ needs to be revised. In the next section, we illustrate how to calculate ePRS for admixed populations according to global and local ancestry patterns.

### Construction of ePRS using global ancestry proportions and local ancestries

#### Global ancestry ePRS

We use global ancestry proportion to calculate global ancestry ePRS (gePRS) and later extend the idea to local ancestries ePRS (lePRS) using local ancestry inference. First, we assume that for each person, the proportions of their entire genomes inherited from each ancestry is known, which are the global ancestry proportion (example is shown in Fig. 1b). We further assume that the various ancestries are uniformly distributed across the genome. Let ${\pi }_{{ik}}$ represent the proportion of the entire genome inherited from ancestry $k$ for the participant $i$, where $k={{\mathrm{1,2}}},\ldots .,K$. To model the genetic pattern of an admixed population, we assume that a genetic variant $j$ follows a mixture model

15 $${g}_{{ij}} \sim {\sum }_{k=1}^{K}{\pi }_{{ik}}\times {P}_{k}({g}_{j};{f}_{j}^{{a}_{k}})$$

where ${P}_{k}({g}_{j};{f}_{j}^{{a}_{k}})$ is the Binomial distribution ${Bin}(2,{f}_{j}^{{a}_{k}})$ with an ancestry-specific allele frequency ${f}_{j}^{{a}_{k}}.$ Using the mixture model (15), we can now compute the gePRS for each individual as

16 $$\begin{array}{c}\begin{array}{c}E\left({{PRS}}_{i}\right)=E\left({\sum }_{j=1}^{p}{\omega }_{j}\times {g}_{{ij}}\right)\\={\sum }_{j=1}^{p}{\omega }_{j}\times E\left({g}_{{ij}}\right)\end{array}\\={\sum }_{j=1}^{p}{\omega }_{j}\times \left\{{\sum }_{k=1}^{K}E\left({g}_{{ij}}|{a}_{i}={a}_{k}\right)\times {\pi }_{{ik}}\right\}\\={\sum }_{j=1}^{p}{\omega }_{j}\times \left\{{\sum }_{k=1}^{K}2\times {f}_{j}^{{a}_{k}}\times {\pi }_{{ik}}\right\}\end{array}$$

From Eq. (16), the gePRS can be interpreted as a weighted combination of ancestry-specific ePRSs, with weights being the global ancestry proportion. We can extend Eq. (12) to calculate the variance of a PRS based on Eq. (15). Assuming that the $p$ variants are independent, the variance of the PRS conditional on global ancestry proportions is:

17 $${Var}\left({{PRS}}_{i}\right)=	 {\sum }_{j=1}^{p}{\left({\omega }_{j}\right)}^{2}\times {Var}\left({g}_{{ij}}\right)=	 {\sum }_{j=1}^{p}{\left({\omega }_{j}\right)}^{2}\\ 	 \times \left\{{\sum }_{k=1}^{K}{Var}({g}_{{ij}}|{a}_{i}={a}_{k})\times {\pi }_{{ik}}\right\}={\sum }_{j=1}^{p}{\left({\omega }_{j}\right)}^{2} \\ 	 \times \left\{{\sum }_{k=1}^{K}2 \times {f}_{j}^{{a}_{k}}\times (1-{f}_{j}^{{a}_{k}})\times {\pi }_{{ik}}\right\}.$$

When the variants used in the PRS are not independent, an alternative way to compute the PRS variance is a data-driven regression model over ancestry proportions. The predicted variance can be computed by first fitting the following regression using unrelated individuals:

18 $$g\left\{E\left({{rPRS}}^{2}\right)\right\}={\sum }_{k=1}^{K-1}{{{{\mathcal{l}}}}}_{k}\times {\pi }_{k}$$

In Eq. (18), we regress the square value of rPRS on global ancestry proportions. Here, we consider the gamma regression since we are predicting the non-negative quantity, the variance of PRS. The motivation for applying Eq. (18) to estimate the variance is drawn from Lambert et al.^12^, who regressed the squared residuals (the differences between the PRS and the predicted PRS) on a set of PCs. After obtaining the estimated coefficient ${\hat{{{{\mathcal{l}}}}}}_{k}$, the predicted variance for PRS for each individual can then be computed by

19 $${{Var}({{PRS}}_{i})}^{{pred}}={\sum }_{k=1}^{K-1}{\hat{{{{\mathcal{l}}}}}}_{k}\times {\pi }_{{ik}}$$

#### Local ePRS

Next, we show how to use local ancestries to construct ePRS, which we call lePRS. Suppose we know the ancestry of each locus and each chromosomal copy. Alleles for each variant are counted by the sum of two chromosomal copies, ${g}_{{ij}}={g}_{{ij}1}+{g}_{{ij}2}$. Assume ${g}_{{ijm}}|{a}_{{ijm}}={a}_{k} \sim {Ber}(1,{f}_{j}^{{a}_{k}})$, where $m={{\mathrm{1,2}}}$ denotes the two copies. The lePRS can be computed as:

20 $$\begin{array}{c}\begin{array}{c}E\left({{PRS}}_{i}\right)=E\left({\sum }_{j=1}^{p}{\omega }_{j}\times {g}_{{ij}}\right)\\={\sum }_{j=1}^{p}{\omega }_{j}\times E\left({g}_{{ij}1}+{g}_{{ij}2}\right)\end{array}\\={\sum }_{j=1}^{p}{\omega }_{j}\times \left(E\left({g}_{{ij}1}\right)+E\left({g}_{{ij}2}\right)\right)\\={\sum }_{j=1}^{p}{\omega }_{j}\times \left\{{\sum }_{m=1}^{2}{\sum }_{k=1}^{K}E({g}_{{ijm}}|{a}_{{ijm}}={a}_{k})\times \Pr ({a}_{{ijm}}={a}_{k})\right\}\end{array}$$

Distinct from gePRS, we assume that each copy’s local ancestry is known and fixed. Hence, by conditioning on ${a}_{{ijm}}={a}_{k}$, the last term of Eq. (20) can be simplified as $\Pr \left({a}_{{ijm}}={a}_{k}\right)=1$ and $\Pr \left({a}_{{ijm}}={a}_{t}{;t}\ne k\right)=0$. Therefore, the lePRS can be expressed as followed

21 $$E\left({{PRS}}_{i}\right) 	= {\sum }_{j=1}^{p}{\omega }_{j}\times \left\{{\sum }_{m=1}^{2}{\sum }_{k=1}^{K}E\left({g}_{{ijm}}|{a}_{{ijm}}={a}_{k}\right)\times \Pr \left({a}_{{ijm}}={a}_{k}\right)\right\}\\ 	={\sum }_{j=1}^{p}{\omega }_{j}\times ({f}_{j}^{{a}_{{ij}1}}+{f}_{j}^{{a}_{{ij}2}})$$

We can also compute the variance of the PRS based on local ancestry information, but add additional assumptions. Here, assuming that the $p$ variants are independent, and that the two chromosomal copies are also independent, the variance of the PRS can be derived as:

22 $${Var}\left({{PRS}}_{i}\right) 	={\sum }_{j=1}^{p}{\left({\omega }_{j}\right)}^{2}\times \left\{{Var}\left({g}_{{ij}1}\right)+{Var}\left({g}_{{ij}2}\right)\right\} \\ 	={\sum }_{j=1}^{p}{\left({\omega }_{j}\right)}^{2}\times \left\{{f}_{j}^{{a}_{{ij}1}}\times \left(1-{f}_{j}^{{a}_{{ij}1}}\right)+{f}_{j}^{{a}_{{ij}2}}\times \left(1-{f}_{j}^{{a}_{{ij}2}}\right)\right\}$$

The technical considerations of computing ePRS and the related metrics are summarized in Supplementary Note 4.

#### Quantile PRS

Based on the ePRS and the PRS variance, we can construct a third metric: the qPRS (qPRS). We assume that given the gePRS or lePRS and the corresponding variance of PRS, the distribution of the PRS for individual $i$ follows the Normal distribution with mean $E({{PRS}}_{i})$ and variance Var$({{PRS}}_{i})$, i.e.,

23 $${{PRS}}_{i}|E\left({{PRS}}_{i}\right),{Var}\left({{PRS}}_{i}\right) \sim N(\mu=E\left({{PRS}}_{i}\right),{\sigma }^{2}={Var}\left({{PRS}}_{i}\right))$$

The qPRS is computed as the percentile of the PRS value conditional on an individual’s ancestral makeup, which can be written as

24 $${{qPRS}}_{i}=\Phi ({{PRS}}_{i};\mu=E\left({{PRS}}_{i}\right),{\sigma }^{2}={Var}\left({{PRS}}_{i}\right))$$

where $\Phi$ is the cumulative Normal distribution function.

#### Parallels with the PC-adjusted PRS framework

The PC-adjusted PRS framework first regresses the observed PRS on a set of PCs (for simplicity, we assume adjusting top $S$ PCs in this model), and uses the residuals as the individual PRS. This can be parallelized with the ePRS framework. First, we can formalize the regression of PRS on PCs under the standard regression model:

25 $$E\left({PRS}|{PCs}\right)={\sum }_{j=1}^{S}{\Theta }_{j}\times {{PC}}_{j}$$

where ${\Theta }_{j}$ is the corresponding effect size of the ${j}^{{th}}$ PC, $j={{\mathrm{1,2}}},\ldots,S$. After fitting the model (25), coefficient estimates are obtained as ${\hat{\Theta }}_{j},j=1,\ldots,S$. While usually not the goal of current framework of PC-adjusted PRS, the PC coefficient estimates can be used to compute an ancestry-predicted PRS value for individual $i$ as:

26 $${{PRS}}_{i}^{{pred}}={\sum }_{j=1}^{S}{\hat{\Theta }}_{j}\times {{PC}}_{{ij}}$$

The individual-level PC-adjusted PRS is computed as the residual from regression model (23), as follows:

27 $${PC}-{adjusted}{{PRS}}_{i}={{PRS}}_{i}-{{PRS}}_{i}^{{pred}}$$

Considering Eqs. (25)–(27), we can draw parallels between the ePRS framework and the PC-adjustment framework: both can be applied to estimate an ancestry-dependent expected value of a PRS, but in different ways, and both obtain the rPRS, subtracting the estimated ancestry-dependent PRS value from the observed PRS value.

### Simulations

#### Generating global ancestry proportions, local ancestries, and allele counts

Without loss of generality, we assume that the genome of each individual in the simulation study is inherited from three ancestries $\left\{{a}_{1},{a}_{2},{a}_{3}\right\}$. The model could be readily extended to a higher number of ancestries. Three ancestries may represent Hispanic/Latino admixed individuals in the U.S., where, for example, ${a}_{1}$ represents European, ${a}_{2}$ represents African, and ${a}_{3}$ represents American ancestry. We assume that ${a}_{1}$ has the highest proportion across the entire genome, and generated the global ancestry proportions by sampling the three proportions of ancestry sequentially from uniform distributions, as follows:

$${\pi }_{{a}_{1}} \sim {Unif}\left(0,1\right)$$

$${\pi }_{{a}_{2}} \sim {Unif}\left(0,1-{\pi }_{{a}_{1}}\right)$$

$${\pi }_{{a}_{3}}=1-{\pi }_{{a}_{1}}-{\pi }_{{a}_{2}}$$

where ${\pi }_{{ak}},k={{\mathrm{1,2,3}}}$ denotes the global ancestry proportion across the genome from ancestries ${a}_{k}$ and ${Unif}\left(a,b\right)$ is the uniform distribution with range $\left(a,b\right)$. After generating ${\pi }_{i}$ for each observation, we next generate local ancestry ${a}_{{ijm}}$. Assuming that each SNP is independent, and the two chromosomal copies are also independent, we generate the local ancestry for each variant and each chromosome separately based on ${{{{\boldsymbol{\pi }}}}}_{i}=({\pi }_{{{ia}}_{1}},{\pi }_{{{ia}}_{2}},{\pi }_{{a}_{3}})$. Thus, we assume that ${a}_{{ijm}}$ follows multinomial distribution, where ${\alpha }_{{ijm}} \sim {Multinomial}\left({p}_{1}={\pi }_{{{ia}}_{1}},{p}_{2}={\pi }_{{{ia}}_{2}}, {p}_{3}=1-{p}_{1}-{p}_{2}\right),j={{\mathrm{1,2}}},\ldots,{p;m}={{\mathrm{1,2}}}$. For each simulated variant, allele counts are then generated with the local ancestry guiding its ancestry-specific allele frequency.

In another set of simulations, we extended the same methodology to generate a six-way admixed population, designed to match the real-world setting of the TOPMed analysis. Individual global ancestry proportions for the six populations were sampled from a Dirichlet distribution, with mean proportions of 0.45 for European, 0.25 for African, 0.15 for Native American, and 0.05 each for East Asian, South Asian, and Middle Eastern ancestries. These proportions were selected to reflect the ancestry patterns observed in the TOPMed dataset. Local ancestry for each individual was then generated from a multinomial distribution using the corresponding global ancestry proportions.

#### Generating PRS

To select the parameters for the simulations, i.e., ancestry-specific allele frequencies and SNP effect sizes, we used summary statistics from a GWAS of SBP, from UKBB and the ICBP consortium^27^. We preprocessed the summary statistics using PLINK with the standard parameters setting (genome-wide significance threshold (5 × 10^−8^), clumping parameter *R*^2^ = 0.1, and the distance was set to 1000 kb), and took 100 SNPs and their estimated effect sizes. Their ancestry-specific frequencies ${f}_{j}^{{\alpha }_{k}}$ were taken to be the estimated frequencies from the GAFA procedure^28^ applied over the TOPMed dataset^60^. For each person and each SNP, we first sampled the local ancestry as described above, followed by sampling of allele count using the ancestry-specific allele frequency corresponding to that SNP and that local ancestry. We finally calculated the PRS as the weighted sum of SNP alleles with weights being the GWAS-estimated effect sizes. The flow chart to generate simulation data is illustrated in Supplementary Fig. 1.

We considered two PRS models. In the primary model, the PRS was assumed to be homogenous across genetic ancestries, i.e., the SNP effects are assumed the same regardless of ancestry, which is the homogeneous weighting PRS. The second model allowed for some heterogeneity by assuming that the SNP effects are sometimes different by ancestry. When simulating heterogeneous weighting PRS settings, we selected the top 10 most frequent SNPs in each ${a}_{2}$ and ${a}_{3}$ ancestries and set different effect sizes for these selected SNPs. Specifically, we set the effect sizes as 1.5 for the selected SNPs in ${a}_{2}$ (African) ancestry and 2 for ${a}_{3}$ (Native American) ancestry.

#### Generating the genetic ancestry confounder ${{{{\boldsymbol{U}}}}}_{{{{\boldsymbol{G}}}}}$

We generated an ancestry-dependent genetic variable potentially confounding the PRS-outcome association. Details are provided in Table 2. Briefly, we considered a few types of unobserved confounder, namely conf1 (local ancestry), conf2 (afr enriched), with computation generally following the same procedure used to generate PRS (i.e., sampling of local ancestry followed by sampling of alleles). Each confounder type was used in separate simulations. For benchmarking, we also used in simulations a variable representing a genetic PC (conf-pc*), which is observed, as a confounder.

For the first genetic confounder (conf1 (local ancestry)), we assume the generated genetic variants shared the same local ancestry interval as the variants that computed the main PRS of interest. Based on the local ancestry interval, we then used ancestry-specific allele frequencies of 100 randomly selected variants from the UKBB-ICBP SBP GWAS to generate genetic data, which is the same procedure for generating observed PRS. The only difference is that the weighting parameters of computing conf1 (local ancestry) are generated from a standard normal distribution.

A second type of genetic confounder (conf2 (afr enriched)) is generated based on a selection of SNPs from the MVP SBP GWAS. These SNP were selected to be enriched in frequency (MAF > 0.4) in the African ancestry population while having MAF < 0.1 in the European ancestral population, as estimated by GAFA. Notably, the distribution of Conf2 (afr enriched) is quite different from that of the observed PRS, leading to strong unknown genetic confounding effect in the simulation settings.

The third continuous confounder, conf-pc*, intends to mimic a scenario where the true confounding is based on only one genetic principal component score. To guide the construction of this variable, we randomly select 100 SNPs from MVP GWAS and generate the weighting parameters from standard Normal distribution. To emphasize, conf-pc* is not calculated via a principal component analysis procedure.

#### Generating outcomes via a data-generating model

We generate a continuous outcome from a linear model summing the effect of the PRS of interest and the genetic confounder through the following model:

$${Y}_{i}={\beta }_{0}+{\beta }_{1}\times {{PRS}}_{i}+\gamma \times \frac{{Confounding}}{{sd}({Confounding})}+{\varepsilon }_{i}$$

Where the genetic confounder is standardized in each simulated dataset so that effect sizes are comparable across simulation settings. We set the PRS effect ${\beta }_{1}$ to 1.5 and varied the effect of the genetic confounder to be $\gamma={{\mathrm{0.5,1,1.5}}}$. The intercept was set to ${\beta }_{0}=1$ across all simulations. Finally, the errors were sampled from a Normal distribution with ${\varepsilon }_{i} \sim N({{\mathrm{0,1}}})$. We sampled $N={{\mathrm{10,000}}}$ observations in each simulation repetition and repeated each simulation setting 1000 times.

#### Estimation of PRS effect in simulations via a working association model

We compare the estimation performance of ${\beta }_{1}$ via eight working association models, M1–M8. In all models, we evaluate the estimated ${\widetilde{\beta }}_{1}$ as an estimator of ${\beta }_{1}$, i.e., as ${\hat{\beta }}_{1}$. Also, all association models use homogeneous weighting PRS.

$$M1:Y={\widetilde{\beta }}_{0}+{\widetilde{\beta }}_{1}\times {PRS}$$

$$M2:Y={\widetilde{\beta }}_{0}+{\widetilde{\beta }}_{1}\times {grPRS}+{\widetilde{\beta }}_{2}\times {gePRS}$$

$$M3:Y={\widetilde{\beta }}_{0}+{\widetilde{\beta }}_{1}\times {lrPRS}+{\widetilde{\beta }}_{2}\times {lePRS}$$

$$M4:Y={\widetilde{\beta }}_{0}+{\widetilde{\beta }}_{1}\times {PRS}+{\widetilde{\beta }}_{2}\times {{\mbox{conf}}-{\mbox{pc}}}^{*}$$

$$M5:Y={\widetilde{\beta }}_{0}+{\widetilde{\beta }}_{1}\times {PRS}+{\sum }_{k=1}^{K-1}{{{{\mathcal{l}}}}}_{k}\times {\pi }_{k}$$

$$M6:{\widetilde{\beta }}_{0}+{\widetilde{\beta }}_{1}\times {PRS}+{\sum }_{j=1}^{2}{\lambda }_{j}\times {{PC}}_{j}$$

$$M7:{\widetilde{\beta }}_{0}+{\widetilde{\beta }}_{1}\times {PRS}+{\sum }_{j=1}^{10}{\lambda }_{j}\times {{PC}}_{j}$$

$$M8:{\widetilde{\beta }}_{0}+{\widetilde{\beta }}_{1}\times {PRS}+{\sum }_{j=1}^{20}{\lambda }_{j}\times {{PC}}_{j}$$

In M1, we estimate PRS association without adjusting for other covariates (we named it as “none” in our results and figures). The proposed ePRS-based approaches are provided in M2 and M3, which adjust for global and lePRS with the corresponding rPRS. M4–M7 models again estimate the effect of the PRS, and further adjust for different ancestry-related measures. M4 uses conf-pc*, which is a benchmark when the true confounding factor is simulated as conf-pc*. M5 adjusts for the global ancestry proportions (“gaProp” in our results and Figures), i.e., it adjusts the PRS association model for $K-1$ covariates, each representing the global ancestry proportion from a corresponding ancestry group, with the remaining ancestry group serving as the reference. M6 to M8 use top 2, 10, and 20 PCs, respectively, where the PCs were calculated based on the genetic dataset used for computing the PRS.

#### Comparison of ePRS and equivalent PC-adjusted PRS measures in simulation study

To compare the two frameworks based on Eqs. (25)–(27), we computed the Pearson correlation between (i) the rPRS and the PC-adjusted PRS, and (ii) the ePRS and the ancestry-predicted PRS. Correlations were calculated across all simulated individuals. For the PC-adjusted PRS framework, we evaluated models adjusting for varying numbers of PCs in Eq. (25), specifically $S={{\mathrm{2,10,20}}}$. For the ePRS framework, we used the gePRS for comparison.

### The TOPMed dataset

The TOPMed project aggregates individual-level data from multiple parent studies. Supplementary Note 7 describes TOPMed parent studies contributing to the analyses.

#### Whole-genome sequencing

We used genetic data from WGS via the TOPMed program^31^ freeze 8 released. Information about genome sequencing, allele calling, and quality control in TOPMed is publicly available at https://www.nhlbiwgs.org/topmed-whole-genome-sequencing-methods-freeze-8. The TOPMed Data Coordinating Center constructed a kinship matrix estimating recent genetic relatedness, the corresponding sparse kinship matrix, where values were set to zero when the genetic relationship was estimated to be more distant than 4th degree relatedness, as well as providing genetic PCs, using the PC-Relate algorithm^61^. In the TOPMed data analysis, we adjusted for 11 genetic PCs in the standard PRS model (except the VTE analysis, in which we adjusted seven genetic PCs according to the previously published paper^35^).

#### Genetic ancestry inference in TOPMed

Ancestry inference was performed by the TOPMed Informatics Research Center. First, local ancestry was inferred using RFMix^33^, with default parameter settings except the following option: –node-size = 5. Then, global ancestry was computed as for each participant as a weighted average of the ancestries in inferred local ancestry intervals. The reference panel used was the HGDP downloaded from the Stanford HGDP website http://hagsc.org/hgdp/files.html. Genomic coordinates were lifted over from genome build 37 to build 38. The 53 HGDP populations were merged into 7 super-populations: Sub-Saharan Africa, Central and South Asia, East Asia, Europe, Native America, Oceania, Middle East. Local ancestry inference was performed in two versions. First, for samples available in TOPMed freeze 6, RFMix V1 was used, and local ancestry was inferred for the autosomes only. Later, for samples participating only in freeze 8 (but not in freeze 6), and for the X-chromosome, local ancestry inference was performed using RFMix V2. Global ancestry proportions for each individual were defined as the genome-wide fractions of sequence assigned to each inferred ancestry, weighted by the corresponding interval lengths. Because the levels of Oceania ancestry were very low in the sample, we did not use it, and instead rescaled, for each person, the other ancestries so that they some to 1. So, if based on the RFMix global ancestry proportions we had ${\hat{p}}_{{africa}}+{\ldots+\hat{p}}_{{europe}}+{\hat{p}}_{{oceania}}$, with ${\hat{p}}_{{ancestry}}$ is the estimated proportion of an ancestry for an individual, we summed the non-oceania proportions for each individual to get $\pi={\hat{p}}_{{africa}}+{\ldots+\hat{p}}_{{europe}}$. The scaled proportions were set to ${{\hat{p}}^{{scaled}}}_{{africa}}={\hat{p}}_{{africa}}/\pi$, etc.

#### Computation of ancestry-specific allele frequencies

Ancestry-specific allele frequencies were computed for all common variants as we previously described^60^, using the GAFA algorithm^28^ that uses global genetic ancestries to deconvolute ancestry-specific allele frequencies. We also computed ancestry-specific allele frequencies using the LAFA algorithm, i.e., using local genetic ancestry patterns at each variant location, however the results are similar, so we chose to move forward with the GAFA-estimated frequencies.

#### Harmonization of self-reported race/ethnicity in TOPMed

Most of the participating studies recruited individuals from pre-defined race/ethnicity categories, which were not always the same. For example, CARDIA uses the descriptor “Black” while JHS uses “African American”. Here, we used the race/ethnicity harmonized categorized prepared by the Data Coordinating Center of TOPMed as part of a phenotype harmonization effort^32^, and as descriptors, we use White (non-Hispanic White or European American), Black (non-Hispanic Black or African American), Asian (Asian or Chinese American), and Hispanic/Latino. We did not use genetic ancestry to categorize race/ethnic categories, as they are not biological and reflect sociopolitical and demographic patterns, though the distribution of genetic ancestry patterns generally differs by groups defined by race and ethnicity

#### Phenotype harmonization

We used 5 continuous phenotypes, SBP, DBP, HDL, LDL, and BMI, all harmonized by the TOPMed DCC^32^. To account for medication use, SBP and DBP values were increased in antihypertensive medication users by 15 and 10 mmHg, respectively. For LDL, the value was adjusted via dividing by 0.7 if the individual took lipid-lowering medication.

Two binary variables used were VTE and OSA. VTE was also harmonized by the TOPMed DCC, as reported in the Seyerle et al.^35^. We harmonized OSA as follows. COPDGene control participants (i.e., individuals without COPD) self-reported doctor-diagnosed OSA. Other participating studies used home sleep apnea testing device to measure the Apnea Hypopnea Index (AHI; measured in ARIC, CARDIA, CHS, and FHS as part of the Sleep Heart Health Study^62^, and in MESA^63^ and JHS^64^), or the Respiratory Event Index (REI; measured in HCHS/SOL^65^). For these cases, we categorized OSA based on AHI/REI$\ge 15$ (i.e., moderat or more severe OSA). Lower AHI/REI values were categorized as no OSA.

#### Selecting SNPs and effect sizes for PRS for the studied traits

We used summary statistics from published GWAS to develop PRS (and ePRS) for each considered trait. These are reported in Supplementary Table 1, which includes information about accessing these summary statistics, the sample sizes of each GWAS population, and the number of variants used to compute PRSs. Briefly, for most traits, we used summary statistics from MVP GWAS^66–69^, which are multi-population GWAS. For BMI, we used the GWAS summary statistics from the Genetic Investigation of Anthropometric Traits (GIANT) consortium meta-analyzed with a UKBB BMI GWAS^70^. The GIANT + UKBB study is mostly based on European ancestry. For each trait and its PRS, in primary analysis, we focused on independent SNPs to allow for calculating the variance of PRS. Thus, we performed clumping using plink^71^. The *p*-value thresholding parameter is set as the genome-wide significance threshold (5 × 10^−8^). The clumping parameter was set to *R*^2^ = 0.1, and the distance was set to 1000 kb. In secondary analysis, we also developed PRSs and corresponding ePRSs based on the same GWAS summary statistics using LDpred2^38^. These are secondary analyses because our focus has been on model-based ePRS measures. In the LDpred2 PRS analyses, we computed PRS variance using the data-driven, regression-based approach. Information about these PRSs and the association analyses is provided in Supplementary Note 5.

#### PRS-outcome association analyses

PRS associations with continuous traits were estimated via linear mixed model, and with binary traits via logistic mixed models. All models used sparse kinship matrix to model a random effect to account for relatedness. Standard PRS model were adjusted for (a) 11 genetic PCs or (b) global ancestry proportions. The ePRS models estimated the global or local rPRS association while adjusting for the corresponding ePRS. Other covariates were often trait-specific: BMI PRS associations were adjusted for sex, age, and squared age term, while SBP, DBP, HDL, and LDL associations were further adjusted for BMI. Logistic mixed model analysis of VTE used a similar approach to that in the published VTE GWAS^35^: we adjusted sex, age, and *lnratio* (log of the ratio between the size of the case stratum and the matched control; to address the differences of case-control matched ratio). Because the model did not converge, we did not include “sample set” as a covariate. OSA analysis adjusted for sex, age, BMI, and the square of the BMI term as fixed effect covariates. All analyses were conducted via the R package *GENESIS* (version 2.32.0^72^) with the fitNullModel function.

#### Assessment of qPRS as an equitable metric for genetic disease risk

We assessed the use of qPRSs for ranking genetic liability quantified by PRS, instead of using the conventional PRSs which are highly affected by ancestral makeup. To this end, we visualized patterns of trait values and qPRS percentiles. For each trait, we stratified individuals to 100 bins defined by values of PRS, rPRS, and qPRS (each separately), and computed the mean value of the trait or proportion of the binary outcome, and visualized these as scatterplots. To demonstrate the impact of ancestral makeup on the PRS metric ranking, we colored each point in the scatterplot by the proportion of African American individuals in the strata (or bin). We chose this population group because the sample is sufficiently large. As the qPRS requires an estimate of the PRS variance, we used the model-based variance from Eq. (17) for PRSs constructed with independent genome-wide significant SNPs (main analysis), and the regression-based variance estimates from Eq. (19) for PRSs developed using genome-wide variants with LDpred2.

### The AoU data analysis

We used srWGS data (version 7) from the AoU study to compute PRS and performed PRS-outcome association analyses. To minimize the memory storage requirements, we used the genomics data pre-filtered by the following criteria: population-specific allele frequency $\ge$ 1% or population-specific allele count > 100 (data from: gs://fc-aou-datasets-controlled/v7/wgs/short_read/snpindel/acaf_threshold_v7.1). Only unrelated individuals were included, with related individuals excluded based on information in: gs://fc-aou-datasets-controlled/v7/wgs/short_read/snpindel/aux/relatedness/relatedness_flagged_samples.tsv. Detailed quality control (QC) procedures, including both genomic and sample QC, can be found in the Genomic Research Data Quality Report: https://support.researchallofus.org/hc/article_attachments/27634053350292.

The analysis focused on adults aged 18–95 with BMI values ranging from 17 to 55, consistent with the selection procedure used in the TOPMed analysis. Sample sizes for self-reported race/ethnicity groups varied slightly depending on the phenotype to analyze. Six binary CVD-related phenotypes were considered: AF, CAD, CVD, HF, HTN, and T2DM. Selection criteria for clinical outcomes, including associated SNOMED codes and OMOP Concept IDs in the AoU study, are detailed in Supplementary Table 7 and Supplementary Note 6.

#### Genetic ancestry inference and ancestry-specific allele frequency

The global ancestry proportion for each individual with srWGS data from the AoU is available at: gs://fc-aou-datasets-controlled/v7/wgs/short_read/snpindel/aux/ ancestry/ancestry_preds.tsv. Six ancestry populations are reported: African/African American (afr), American Admixed/Latino (amr), East Asian (eas), European (Eeur), Middle Eastern (mid), and South Asian (sas). These categories align with the ancestry definitions used in gnomAD, the HGDP, and the 1000 Genomes Project. Additional details on computing categorical ancestry for individuals in AoU are provided in the Genomic Quality Report (https://support.researchallofus.org/hc/article_attachments/27634053350292)

The ancestry-specific allele frequency used for computing ePRS were obtained from gnomAD (version 3.1.2). The data can be access at: gs://gcp-public-data--gnomad/release/3.1.2/ht/genomes/gnomad.genomes.v3.1.2.hgdp_1kg_subset_variant_annotations.ht using Hail procedures. The genetic ancestry group labels from gnomAD are harmonized with those from HGDP and the 1000 Genomes Project, aligning with the definitions used in AoU. Further details about genetic ancestry inference in gnomAD are available at: https://gnomad.broadinstitute.org/news/2023−11-genetic-ancestry/

#### PRS-outcome association analysis

Since only unrelated individuals were included in this analysis, logistic regression was used to conduct PRS-outcome associations. All models were adjusted for sex at birth, BMI, age, and the square term of age. For the conventional PRS model, either 16 genetic PCs or global ancestry proportions were included to account for population stratification bias. For the ePRS framework, the outcome of interest was regressed on the rPRS with the global ePRS included as an adjustment in the model. Additionally, PC-adjusted PRS were analyzed instead of using unadjusted PRS as covariates, and the 16 genetic PCs were also adjusted in the model for the comparison purposes.

#### Comparison of ePRS and equivalent PC-adjusted PRS measures in AoU analysis

We computed the Pearson correlation between (i) the global rPRS and the PC-adjusted PRS, and (ii) the global ePRS and the ancestry-predicted PRS, following the same analysis performed in simulation study. The PC-adjusted PRS was computed using Eq. (27), where 16 PCs computed from the AoU platform were included in the model. Correlations were calculated separately within each self-reported race/ethnicity group.

### Reporting summary

Further information on research design is available in the Nature Portfolio Reporting Summary linked to this article.

## Supplementary information

Supplementary Material

Description of Additional Supplementary Files

Supplementary Data 1

Supplementary Data 2

Supplementary Data 3

Reporting Summary

Transparent Peer Review file

## Source data

Source Data

## Data Availability

TOPMed freeze 8 WGS data and harmonized BP and lipid phenotypes are available by application to dbGaP according to the study specific accessions: Amish: phs000956 [https://www.ncbi.nlm.nih.gov/projects/gap/cgi-bin/study.cgi?study_id=phs000956.v5.p1], ARIC: phs001211 [https://www.ncbi.nlm.nih.gov/projects/gap/cgi-bin/study.cgi?study_id=phs001211.v5.p4], CARDIA: phs001612 [https://www.ncbi.nlm.nih.gov/projects/gap/cgi-bin/study.cgi?study_id=phs001612.v3.p3], CFS: phs000954 [https://www.ncbi.nlm.nih.gov/projects/gap/cgi-bin/study.cgi?study_id=phs000954.v5.p2], CHS: phs001368 [https://www.ncbi.nlm.nih.gov/projects/gap/cgi-bin/study.cgi?study_id=phs001368.v4.p2], COPDGene: phs000951 [https://www.ncbi.nlm.nih.gov/projects/gap/cgi-bin/study.cgi?study_id=phs000951.v6.p5], FHS: phs000974 [https://www.ncbi.nlm.nih.gov/projects/gap/cgi-bin/study.cgi?study_id=phs000974.v6.p5], GENOA: phs001345 [https://www.ncbi.nlm.nih.gov/projects/gap/cgi-bin/study.cgi?study_id=phs001345.v3.p1], HCHS/SOL: phs001395 [https://www.ncbi.nlm.nih.gov/projects/gap/cgi-bin/study.cgi?study_id=phs001395.v3.p2], HVH: phs000993 [https://www.ncbi.nlm.nih.gov/projects/gap/cgi-bin/study.cgi?study_id=phs000993.v5.p2], JHS: phs000964 [https://www.ncbi.nlm.nih.gov/projects/gap/cgi-bin/study.cgi?study_id=phs000964.v6.p2], Mayo VTE: phs001402 [https://www.ncbi.nlm.nih.gov/projects/gap/cgi-bin/study.cgi?study_id=phs001402.v4.p1], MESA: phs001211 [https://www.ncbi.nlm.nih.gov/projects/gap/cgi-bin/study.cgi?study_id=phs001211.v5.p4], WHI: phs001237 [https://www.ncbi.nlm.nih.gov/projects/gap/cgi-bin/study.cgi?study_id=phs001237.v4.p2]. Summary statistics from MVP GWAS are available from dbGaP by application to study accession phs001672 [https://www.ncbi.nlm.nih.gov/projects/gap/cgi-bin/study.cgi?study_id=phs001672.v13.p1]. Summary statistics from GIANT + UKBB GWAS are publicly available and were downloaded from https://portals.broadinstitute.org/collaboration/giant/index.php/GIANT_consortium_data_files. Data needed to construct the reported PRSs in this study include variants, alleles, and weights for each of the PRS are deposited on the figshare repository: 10.6084/m9.figshare.25336294 (ref. ^73^) and on the PGS catalog (publication ID: PGP000786 and score IDs: PGS012514−12529). A dataset with ancestry-specific allele frequencies computed using GAFA on the TOPMed dataset for Europe, Africa, Middle East, East Asia, South Asia, and America ancestries for HapMap3 variants, which are recommended for use by the LDpred2 software, are available on the figshare repository (ref. ^73^). The summary statistics and ancestry-specific allele frequency used in AoU analysis can also be found in the figshare repository (ref. ^73^). Data from the NIH AoU study are available via institutional data access for researchers who meet the criteria for access to confidential data. To register as a researcher with AoU, researchers may use the following URL and complete the laid-out steps: https://www.researchallofus.org/register/. The srWGS genomic data were available on gs://fc-aou-datasets-controlled/v7/wgs/short_read/. Ancestry-specific allele frequencies matching the AoU ancestries can be downloaded from gnomAD Google Cloud Public Datasets [gs://gcp-public-data--gnomad/release/3.1.2/ht/genomes/gnomad.genomes.v3.1.2.hgdp_1kg_subset_variant_annotations.ht]. The raw data from the simulation studies used for visualization of the results are available in this GitHub repository [https://github.com/Gene-Huang/Expected_PRS/tree/main/simulation_results] and via Zenodo at 10.5281/zenodo.18880436 (ref. ^74^). Source data are provided with this paper.

## Code availability

R code used for simulation studies and for constructing ePRSs are available on this GitHub repository [https://github.com/Gene-Huang/Expected_PRS] and via Zenodo at 10.5281/zenodo.18880436 (ref. ^74^).
