# Supplementary material for: Admixture-informed polygenic risk reporting using the ePRS framework
Source: Nat Commun. 2026 Apr 30;17:5741. doi: 10.1038/s41467-026-72457-x (PMC13323344; doi:10.1038/s41467-026-72457-x)
Supplement: Supplementary file 2 — Description of Additional Supplementary Files [file 41467_2026_72457_MOESM2_ESM.pdf]

Description of Additional Supplementary Files

File Name: Supplementary Data 1.

Description: Summary statistics of TOPMed PRS-outcome association estimates from both combined and ancestry-stratified analyses, including results from the ePRS framework and other ancestry-adjusted PRS models.

File Name: Supplementary Data 2.

Description: Summary statistics of TOPMed LDpred2 PRS-outcome association estimates from both combined and ancestry-stratified analyses, including results from the ePRS framework and other ancestry-adjusted PRS models.

File Name: Supplementary Data 3.

Description: Summary statistics of AoU PRS-outcome association estimates from both combined and ancestry-stratified analyses, including results from the ePRS framework and other ancestry-adjusted PRS models.
