# Supplementary material for: Admixture-informed polygenic risk reporting using the ePRS framework
Source: Nat Commun. 2026 Apr 30;17:5741. doi: 10.1038/s41467-026-72457-x (PMC13323344; doi:10.1038/s41467-026-72457-x)
Supplement: Supplementary file 6 — Reporting Summary [file 41467_2026_72457_MOESM6_ESM.pdf]

nature portfolio

Corresponding author(s): Tamar Sofer

Last updated by author(s): Mar 5, 2026

Reporting Summary

Nature Portfolio wishes to improve the reproducibility of the work that we publish. This form provides structure for consistency and transparency in reporting. For further information on Nature Portfolio policies, see our Editorial Policies and the Editorial Policy Checklist.

Statistics

For all statistical analyses, confirm that the following items are present in the figure legend, table legend, main text, or Methods section.

n/a	Confirmed	
☐	☒	The exact sample size (*n*) for each experimental group/condition, given as a discrete number and unit of measurement
☒	☐	A statement on whether measurements were taken from distinct samples or whether the same sample was measured repeatedly
☐	☒	The statistical test(s) used AND whether they are one- or two-sided<br>*Only common tests should be described solely by name; describe more complex techniques in the Methods section.*
☐	☒	A description of all covariates tested
☐	☒	A description of any assumptions or corrections, such as tests of normality and adjustment for multiple comparisons
☐	☒	A full description of the statistical parameters including central tendency (e.g. means) or other basic estimates (e.g. regression coefficient) AND variation (e.g. standard deviation) or associated estimates of uncertainty (e.g. confidence intervals)
☐	☒	For null hypothesis testing, the test statistic (e.g. *F*, *t*, *r*) with confidence intervals, effect sizes, degrees of freedom and *P* value noted<br>*Give P values as exact values whenever suitable.*
☒	☐	For Bayesian analysis, information on the choice of priors and Markov chain Monte Carlo settings
☒	☐	For hierarchical and complex designs, identification of the appropriate level for tests and full reporting of outcomes
☐	☒	Estimates of effect sizes (e.g. Cohen's *d*, Pearson's *r*), indicating how they were calculated

Our web collection on statistics for biologists contains articles on many of the points above.

Software and code

Policy information about availability of computer code

Data collection	No software was used.
Data analysis	The following softwares were used: R 4.3.1, PRSice 2.3.5, PLINK v1.9, bigsnpr R Package v1.12.2, GENESIS R Package v2.32.0, RFMix v2. R code used for simulation studies and for constructing ePRSs are available on the GitHub repository: https://github.com/Gene-Huang/Expected_PRS and via Zenodo at https://doi.org/10.5281/zenodo.18880436. The summary statistics and ancestry-specific allele frequency used for PRSs and ePRSs computation are available on the figshare repository: https://doi.org/10.6084/m9.figshare.25336294. For All of Us (AoU) analysis, the Python version 3.10.12 were used. Analyses were performed on the AoU Researcher Workbench.

For manuscripts utilizing custom algorithms or software that are central to the research but not yet described in published literature, software must be made available to editors and reviewers. We strongly encourage code deposition in a community repository (e.g. GitHub). See the Nature Portfolio guidelines for submitting code & software for further information.

Data

Policy information about availability of data

All manuscripts must include a data availability statement. This statement should provide the following information, where applicable:

- Accession codes, unique identifiers, or web links for publicly available datasets
- A description of any restrictions on data availability
- For clinical datasets or third party data, please ensure that the statement adheres to our policy

TOPMed freeze 8 WGS data and harmonized BP and lipid phenotypes are available by application to dbGaP according to the study specific accessions: Amish: phs000956 [https://www.ncbi.nlm.nih.gov/projects/gap/cgi-bin/study.cgi?study_id=phs000956.v5.p1], ARIC: phs001211 [https://www.ncbi.nlm.nih.gov/projects/gap/cgi-bin/study.cgi?study_id=phs001211.v5.p4], CARDIA: phs001612 [https://www.ncbi.nlm.nih.gov/projects/gap/cgi-bin/study.cgi?study_id=phs001612.v3.p3], CFS: phs000954 [https://www.ncbi.nlm.nih.gov/projects/gap/cgi-bin/study.cgi?study_id=phs000954.v5.p2], CHS: phs001368 [https://www.ncbi.nlm.nih.gov/projects/gap/cgi-bin/study.cgi?study_id=phs001368.v4.p2], COPDGene: phs000951 [https://www.ncbi.nlm.nih.gov/projects/gap/cgi-bin/study.cgi?study_id=phs000951.v6.p5], FHS: phs000974 [https://www.ncbi.nlm.nih.gov/projects/gap/cgi-bin/study.cgi?study_id=phs000974.v6.p5], GENOA: phs001345 [https://www.ncbi.nlm.nih.gov/projects/gap/cgi-bin/study.cgi?study_id=phs001345.v3.p1], HCHS/SOL: phs001395 [https://www.ncbi.nlm.nih.gov/projects/gap/cgi-bin/study.cgi?study_id=phs001395.v3.p2], HVH: phs000993 [https://www.ncbi.nlm.nih.gov/projects/gap/cgi-bin/study.cgi?study_id=phs000993.v5.p2], JHS: phs000964 [https://www.ncbi.nlm.nih.gov/projects/gap/cgi-bin/study.cgi?study_id=phs000964.v6.p2], Mayo VTE: phs001402 [https://www.ncbi.nlm.nih.gov/projects/gap/cgi-bin/study.cgi?study_id=phs001402.v4.p1], MESA: phs001211 [https://www.ncbi.nlm.nih.gov/projects/gap/cgi-bin/study.cgi?study_id=phs001211.v5.p4], WHI: phs001237 [https://www.ncbi.nlm.nih.gov/projects/gap/cgi-bin/study.cgi?study_id=phs001237.v4.p2]. Summary statistics from MVP GWAS are available from dbGaP by application to study accession phs001672 [https://www.ncbi.nlm.nih.gov/projects/gap/cgi-bin/study.cgi?study_id=phs001672.v13.p1]. Summary statistics from GIANT + UKBB GWAS are publicly available and were downloaded from https://portals.broadinstitute.org/collaboration/giant/index.php/GIANT_consortium_data_files. Data needed to construct the reported PRSs in this study include variants, alleles, and weights for each of the PRS are deposited on the figshare repository: https://doi.org/10.6084/m9.figshare.25336294 (ref.73) and on the PGS catalog. A dataset with ancestry-specific allele frequencies computed using GAFA on the TOPMed dataset for Europe, Africa, Middle East, East Asia, South Asia, and America ancestries for HapMap3 variants, which are recommended for use by the LDpred2 software, are available on the figshare repository (ref.73). The summary statistics and ancestry-specific allele frequency used in AoU analysis can also be found in the figshare repository (ref.73). Data from the NIH AoU study are available via institutional data access for researchers who meet the criteria for access to confidential data. To register as a researcher with AoU, researchers may use the following URL and complete the laid-out steps: https:// www.researchallofus.org/register/. The srWGS genomic data were available on: gs://fc-aou-datasets-controlled/v7/wgs/short_read/. Ancestry-specific allele frequencies matching the AoU ancestries can be downloaded from gnomAD Google Cloud Public Datasets: gs://gcp-public-data--gnomad/release/3.1.2/ht/genomes/gnomad.genomes.v3.1.2.hgdp_1kg_subset_variant_annotations.ht. The raw data from the simulation studies used for visualization of the results are available in the GitHub repository at: https://github.com/Gene-Huang/Expected_PRS/simulation_results. Source data are provided with this paper.

Research involving human participants, their data, or biological material

Policy information about studies with human participants or human data. See also policy information about sex, gender (identity/presentation), and sexual orientation and race, ethnicity and racism.

Reporting on sex and gender	Our data included self-reported males and females. In the TOPMed dataset, self-reported males and females also passed chromosomal checks, requiring two X chromosomes for females and one X and one Y chromosome for males. For the AoU analysis, we included only individuals whose self-reported "sex at birth" was recorded as either Female or Male.
Reporting on race, ethnicity, or other socially relevant groupings	In TOPMed analysis, the data included each individual's self-reported race/ethnicity information, including African American, Asian American, European American, and Hispanic/Latino. In AoU, we only included self-reported non-Hispanic Asian, non-Hispanic White, non-Hispanic Black, and Hispanic/Latino individuals.
Population characteristics	Individuals are all adults with BMI values less than 55 from multiple cohorts. Population characteristics are reported in Supplementary Tables 2-8.
Recruitment	Multiple studies participated in the analyses in this manuscript. All the detail can be found in supplementary material in the study description section (supplementary note 5 for AoU and supplementary note 6 for TOPMed) and Acknowledgement section in main manuscript.
Ethics oversight	Amish:<br>All study protocols were approved by the institutional review board at the University of Maryland Baltimore. Informed consent was obtained from each study participant.<br>ARIC:<br>The ARIC study has been approved by a single Institutional Review Board (sIRB) at Johns Hopkins School of Medicine and Institutional Review Boards (IRB) at all participating institutions: University of North Carolina at Chapel Hill IRB, Johns Hopkins University School of Public Health IRB, University of Minnesota IRB, Wake Forest University Health Sciences IRB, and University of Mississippi Medical Center IRB. Study participants provided written informed consent at all study visits.<br>CARDIA:<br>All CARDIA participants provided informed consent, and the study was approved by the Institutional Review Boards of the University of Alabama at Birmingham and the University of Texas Health Science Center at Houston.<br>CFS:<br>Cleveland Family Study was approved by the Institutional Review Board (IRB) of Case Western Reserve University and Mass General Brigham (formerly Partners HealthCare). Written informed consent was obtained from all participants.

CHS:
All CHS participants provided informed consent, and the study was approved by the Institutional Review Board [or ethics review committee] of University Washington.
COPDGene:
All COPDGene participants provided written informed consent, and the study was approved by the Institutional Review Boards of the participating clinical centers.
FHS:
The Framingham Heart Study was approved by the Institutional Review Board of the Boston University Medical Center. All study participants provided written informed consent.
GENOA:
Written informed consent was obtained from all subjects and approval was granted by participating institutional review boards (University of Michigan, University of Mississippi Medical Center, and Mayo Clinic).
HCHS/SOL:
This study was approved by the institutional review boards (IRBs) at each field center, where all participants gave written informed consent, and by the Non-Biomedical IRB at the University of North Carolina at Chapel Hill, to the HCHS/SOL Data Coordinating Center. All IRBs approving the study are: Non-Biomedical IRB at the University of North Carolina at Chapel Hill. Chapel Hill, NC; Einstein IRB at the Albert Einstein College of Medicine of Yeshiva University. Bronx, NY; IRB at Office for the Protection of Research Subjects (OPRS), University of Illinois at Chicago. Chicago, IL; Human Subject Research Office, University of Miami. Miami, FL; Institutional Review Board of San Diego State University. San Diego, CA.
HVH:
Study approval was granted by the human subjects committee at Group Health, and written informed consent was provided by all study participants.
JHS:
The Institutional Review Boards at Jackson State University, Tougaloo College, and the University of Mississippi Medical Center approved the study, and all participants provided written informed consent.
Mayo VTE:
All Mayo-VTE participants provided informed consent and the study was approved by the Institutional Review Board of Mayo Clinic, Rochester, MN.
MESA:
All MESA participants provided written informed consent, and the study was approved by the Institutional Review Boards at The Lundquist Institute (formerly Los Angeles BioMedical Research Institute) at Harbor-UCLA Medical Center, University of Washington, Wake Forest School of Medicine, Northwestern University, University of Minnesota, Columbia University, and Johns Hopkins University.
WHI:
All WHI participants provided informed consent and the study was approved by the Institutional Review Board (IRB) of the Fred Hutchinson Cancer Research Center.

The All of Us research program was approved by a single IRB, the “All of Us IRB”, which is charged with reviewing the protocol, informed consent, and other participant-facing materials for the All of Us Research Program. The IRB follows the regulations and guidance of the Office for Human Research Protections (https://www.hhs.gov/ohrp/index.html) for all studies, ensuring that the rights and welfare of research participants are overseen and protected uniformly. More information is provided online https://allofus.nih.gov/about/who-we-are/institutional-review-board-irb-of-all-of-us-research-program and in the All of Us design paper.

Note that full information on the approval of the study protocol must also be provided in the manuscript.

Field-specific reporting

Please select the one below that is the best fit for your research. If you are not sure, read the appropriate sections before making your selection.

☒ Life sciences ☐ Behavioural & social sciences ☐ Ecological, evolutionary & environmental sciences

For a reference copy of the document with all sections, see nature.com/documents/nr-reporting-summary-flat.pdf

Life sciences study design

All studies must disclose on these points even when the disclosure is negative.

Sample size

Across traits, up to 49,626 individuals from TOPMed were included in a given analysis, with sample sizes and parent studies varying by trait. All details can be found in Supplementary Tables 2–6. For the AoU analysis, up to 204,000 individuals were included in the PRS analysis, with sample sizes varying depending on the traits analyze. More details can be found in Supplementary Table 8.

Data exclusions

The TOPMed data analyses include adults with BMI values less than 55. After the data preprocessing, we did not include individuals from COPDGene from TOPMed in our data analyses. The AoU analysis was restricted to adults aged 18 to 95 with BMI values between 17 and 55, consistent with the preprocessing procedures used in the TOPMed analysis. Individuals with documented deaths in the electronic health

	records were excluded. Finally, related individuals were removed based on information from gs://fc-aou-datasets-controlled/v7/wgs/short_read/snpindel/aux/relatedness/relatedness_flagged_samples.tsv.
Replication	We applied the proposed framework on the AoU platform to perform PRS analyses for six CVD-related phenotypes using publicly available GWAS summary statistics and ancestry-specific allele frequencies
Randomization	There was no randomization, this is an observational study.
Blinding	There was no blinding, this is an observational study (no treatment).

Reporting for specific materials, systems and methods

We require information from authors about some types of materials, experimental systems and methods used in many studies. Here, indicate whether each material, system or method listed is relevant to your study. If you are not sure if a list item applies to your research, read the appropriate section before selecting a response.

Materials & experimental systems

n/a	Involved in the study
☒	☐ Antibodies
☒	☐ Eukaryotic cell lines
☒	☐ Palaeontology and archaeology
☒	☐ Animals and other organisms
☒	☐ Clinical data
☒	☐ Dual use research of concern
☒	☐ Plants

Methods

n/a	Involved in the study
☒	☐ ChIP-seq
☒	☐ Flow cytometry
☒	☐ MRI-based neuroimaging

Plants

Seed stocks	*Report on the source of all seed stocks or other plant material used. If applicable, state the seed stock centre and catalogue number. If plant specimens were collected from the field, describe the collection location, date and sampling procedures.*
Novel plant genotypes	*Describe the methods by which all novel plant genotypes were produced. This includes those generated by transgenic approaches, gene editing, chemical/radiation-based mutagenesis and hybridization. For transgenic lines, describe the transformation method, the number of independent lines analyzed and the generation upon which experiments were performed. For gene-edited lines, describe the editor used, the endogenous sequence targeted for editing, the targeting guide RNA sequence (if applicable) and how the editor was applied.*
Authentication	*Describe any authentication procedures for each seed stock used or novel genotype generated. Describe any experiments used to assess the effect of a mutation and, where applicable, how potential secondary effects (e.g. second site T-DNA insertions, mosiacism, off-target gene editing) were examined.*
