# Supplementary material for: Admixture-informed polygenic risk reporting using the ePRS framework
Source: Nat Commun. 2026 Apr 30;17:5741. doi: 10.1038/s41467-026-72457-x (PMC13323344; doi:10.1038/s41467-026-72457-x)
Supplement: Supplementary file 7 — Transparent Peer Review file [file 41467_2026_72457_MOESM7_ESM.pdf]

nature portfolio

# Peer Review File

# Admixture-informed polygenic risk reporting using the ePRS framework

Corresponding Author: Dr Tamar Sofer

This manuscript has been previously reviewed at another journal. This document only contains information relating to versions considered at Nature Communications.

Version 0:

Reviewer comments:

Reviewer #3

(Remarks to the Author)
In this paper, Huang et al. introduced a novel polygenic risk score (PRS) framework aimed at improving the quantification of polygenic risk across diverse ancestral groups, including admixed populations. As acknowledged by the authors and previous reviewers, enhancing the performance of PRS across different ancestral groups is an important and timely area of research. The paper provides comprehensive analyses and introduces an innovative method, but its overall presentation, particularly regarding mathematical symbols, equations, and variable naming, could benefit from greater clarity and accessibility. I found the paper challenging to follow, and I will attempt to summarize my understanding. Please correct me if I have misunderstood any key points.

The core argument of the paper seems to be that for each population or admixed population, there exists a "background genetic signal" calculated as the weighted sum of population allele frequencies for the variants of interest (expected PRS). This background signal accounts for individual admixture proportions and allows the authors to compute the "true" polygenic risk (residual PRS) after adjusting for the baseline. By doing so, the adjusted PRS becomes population-agnostic, enabling fairer comparisons across groups. The authors further refine this concept by introducing local admixture proportions for each variant, which provide a more granular background adjustment. Using simulations and real data, they demonstrate that this adjusted PRS framework outperforms traditional methods.

If my understanding is correct, then here are some of the comments for the paper:

1. Abstract: One of the previous reviewers highlighted the role of linkage disequilibrium (LD) in the limited portability of PRS across populations. The current abstract primarily emphasizes population-specific allele frequencies as the main factor. It might be helpful to either include LD as a contributing factor or clarify that population-specific frequencies are only one of several possible explanations.

2. The second paragraph of the introduction feels unnecessarily complex. A more straightforward approach might be: "Recently, ancestry adjustments have been performed by regressing principal components (PCs) out of PRS using linear models (e.g., Khera et al.). While this framework removes ancestry-related genetic effects, PCs vary across datasets, and there is no standard for the number of PCs to include......" The authors can then introduce the concept of baseline genetic liability or 'background' genetic liability as an alternative approach.

3. Terminology: Consider revising the naming conventions for key terms to improve accessibility for non-statisticians. For instance:

"Expected PRS" could be renamed "Background PRS" or "Baseline PRS."
"Residual PRS" might be clearer as "Adjusted PRS."
"Quantile PRS" could benefit from a brief explanation, as its meaning is not immediately intuitive.

4. Some section of the paper is a bit confusing. For example, I am uncertain of the purpose of the PRS-outcome association model section, and equation 1. Based on my understanding of the paper so far, the Ug should be part of the PRS, that's why

it introduce the bias to the overall PRS? If Ug isn't part of the PRS, then we should still be able to segregate samples based on their PRS as the equation kind of suggest Ug is independent of the PRS? And if we then go to equation 2, the authors are suggesting that the Ug is now accounted for, so I'd imagine \beta_1 PRS + \rho U_g is approximated by \hat{\beta_1} rPRS + \sigma ePRS?

5. Maybe the authors can streamline the section on Overview of the ePRS framework. For example, the ePRS could first be described as the weighted sum of population allele frequencies, representing an individual's ancestral background. This concept could then be extended to lePRS, which uses local admixture proportions for each variant.

6. While the simulations are a useful starting point, they appear overly simplified. For instance:
- The absence of LD between variants may limit the applicability of findings, as LD is considered a major factor affecting PRS portability. I understand this can be difficult to address. This should mainly be stated as a limitation of the current study.
- Using the top 100 SNPs from an external GWAS with fixed effect sizes may not fully capture the complexities of real-world polygenic traits. Simulating effect sizes directly based on expected heritability could provide more robust insights.
- The naming conventions for simulation conditions (e.g., conf-PRS1, conf-PRS2) are confusing and could benefit from clearer descriptions. For example, conf-PRS2 appears to focus on variants that is only common in African, but this is not immediately obvious from the name.

7. In the paper, the authors introduced a gaPRS but it is not clear what's the difference between gaPRS and gePRS?

8. Figure 5c could be improved by using a different color scheme or format. For instance, plotting the proportion of African samples on the y-axis and coloring based on mean trait values might better illustrate the distribution differences between adjusted and unadjusted PRS.

9. Reviewer #2's concern about the similarity in performance between ePRS and PC-adjusted PRS is valid. To address this, the authors could:

- Conduct analyses with smaller sample sizes to explore potential advantages of ePRS.
- Investigate the sensitivity of PC-adjustment methods (e.g., varying the number of PCs).
- Assess whether ePRS correlates with population disease prevalence, which could add valuable insights.
- The authors can also stress that the ePRS only rely on the proportion of different ancestry groups, which is a continuous measure, which allow it to be similar to the PC approach

Minor comments:
- It would be helpful if the authors can use multi-line &= equations, instead of cramping multiple lines of equations together, which makes it impossible to read.

Sam

Version 1:

Reviewer comments:

Reviewer #3

(Remarks to the Author)
The authors have made significant strides in clarifying key aspects of the paper, which has greatly improved my understanding of its objectives. I appreciate the effort to address previous concerns, particularly regarding the paper's focus. One of the key points of clarification is the distinction between introducing a new polygenic risk score (PRS) methodology and providing a more robust reporting framework for PRS in admixed populations. This clarification is critical and shifts the interpretation of the paper's contributions. To ensure this distinction is clear to all readers, I strongly recommend emphasizing this in both the abstract and introduction. For example, citing relevant works such as Wand et al. (2021, Nature) and the eMERGE consortium's initiative (Lennon et al., 2024, Nature Medicine) could help contextualize the paper within the broader literature. Currently, the abstract and introduction might give the impression that the authors are proposing an entirely new PRS framework, which could lead to misinterpretation.

The paper provides valuable insights, but I do have some concerns about the simulation design. Specifically, the simulations appear overly simplified, as they do not account for linkage disequilibrium (LD) and lack control over the heritability of the simulated phenotype. While all simulations inherently rely on assumptions, the current design may introduce biases that limit the generalizability of the conclusions. However, the results from the TopMed and All of Us (AoU) datasets are much more compelling, as they demonstrate how the method performs in real-world scenarios. These results strengthen the paper and should be highlighted further.

I also have a few specific suggestions that could help refine the analysis and improve the clarity of the results:

- For Figure 7, could you clarify whether the PRS was adjusted for principal components (PCs) as described in Khera et al.? For example, was the association estimated using a model like Y ~ PC-adjusted-PRS + PCs? If not, and the association was modeled as Y ~ PC-adjusted PRS, why wasn't this approach also included in Figure 6 for consistency?
- How many PCs were used for the adjustment?
- In Figures 6 and 7, if the scales of the PRS differ, it might be more informative to plot z-scores instead of betas. This would allow for a fairer comparison across methods.
- If the ePRS and rPRS perform similarly to the PC-adjusted PRS, this would support their use as viable alternatives. To strengthen this argument, I suggest adding a supplementary analysis to Figures 6 and 7. Specifically, you could explore how varying the number of PCs used for adjustment impacts the risk assessment. For example, if currently only 10 PCs were adjusted, also plot the results when say 20PCs were adjusted. This would further highlight the limitations of PC-adjusted PRS and bolster the case for your proposed method.

Minor Comment:
- Is Figure 5 intended to have coloring? Currently it seems like color is missing
- Consider rearranging the figures so that lines 441 and 442 are presented together for better readability.
- There is a typo on line 1112—"EPRS" should be "ePRS."

Version 2:

Reviewer comments:

Reviewer #3

(Remarks to the Author)
In this revision, Huang et al. have successfully addressed the majority of my concerns. The inclusion of simulation analyses is a valuable addition and strengthens the study. I have only one minor comment regarding the simulation analyses, which does not compromise the validity of the study, as all methods were applied to the same set of simulated data:

While it is reasonable to focus on a single chromosome in the simulation, careful consideration is required when selecting the number of "causal" variants and the simulated heritability. For instance, with only 1,000 causal variants and a heritability of 0.8, the simulated effect sizes for individual variants could exceed what is typically observed in genetic studies, depending on the effect size distribution used. This could have implications for the interpretation of results derived from such simulations. I recommend the authors acknowledge this limitation and provide a brief discussion of its potential impact on their findings. This suggestion is intended as a minor clarification and does not require further revisions or resubmission.

**Open Access** This Peer Review File is licensed under a Creative Commons Attribution 4.0 International License, which permits use, sharing, adaptation, distribution and reproduction in any medium or format, as long as you give appropriate credit to the original author(s) and the source, provide a link to the Creative Commons license, and indicate if changes were made.
In cases where reviewers are anonymous, credit should be given to 'Anonymous Referee' and the source.
The images or other third party material in this Peer Review File are included in the article's Creative Commons license,

unless indicated otherwise in a credit line to the material. If material is not included in the article's Creative Commons license and your intended use is not permitted by statutory regulation or exceeds the permitted use, you will need to obtain permission directly from the copyright holder.
To view a copy of this license, visit https://creativecommons.org/licenses/by/4.0/

# Revision of manuscript “The expected polygenic risk score (ePRS) framework: an equitable metric for quantifying polygenetic risk via modeling of ancestral makeup”: response to reviewer 3

**Comments from Reviewer 3:**

In this paper, Huang et al. introduced a novel polygenic risk score (PRS) framework aimed at improving the quantification of polygenic risk across diverse ancestral groups, including admixed populations. As acknowledged by the authors and previous reviewers, enhancing the performance of PRS across different ancestral groups is an important and timely area of research. The paper provides comprehensive analyses and introduces an innovative method, but its overall presentation, particularly regarding mathematical symbols, equations, and variable naming, could benefit from greater clarity and accessibility. I found the paper challenging to follow, and I will attempt to summarize my understanding. Please correct me if I have misunderstood any key points.

The core argument of the paper seems to be that for each population or admixed population, there exists a "background genetic signal" calculated as the weighted sum of population allele frequencies for the variants of interest (expected PRS). This background signal accounts for individual admixture proportions and allows the authors to compute the "true" polygenic risk (residual PRS) after adjusting for the baseline. By doing so, the adjusted PRS becomes population-agnostic, enabling fairer comparisons across groups. The authors further refine this concept by introducing local admixture proportions for each variant, which provide a more granular background adjustment. Using simulations and real data, they demonstrate that this adjusted PRS framework outperforms traditional methods.

Thank you for the valuable comments, which have guided us in revising the manuscript for re-submission to Nature Communications. We have updated the main manuscript, with particular emphasis on improving the Introduction and Methods sections to better clarify our main contributions and enhance accessibility for a broader readership. We conducted further simulations to compare the ePRS and PC-adjusted approaches across varying sample sizes, as well as sensitivity analyses assessing the impact of adjusting for different numbers of PCs.

Also, we note that your overall understanding is correct, though there are some nuances to clarify. For example, our goal is not necessarily to compare groups. Rather, to provide an individual, regardless of their specific ancestry patterns, measures that, in summary, (1) acknowledge their ancestry make-up, and (2) are independent of this make-up. This work does not solve the problem of having PRSs that are more powerful in a specific group: it offers measures encapsulating new PRS interpretations. We agree with your assessment that it has been challenging to make this point clear and the technical wording we used perhaps didn’t help. We hope that this version of the manuscript is better.

If my understanding is correct, then here are some of the comments for the paper:

1. Abstract: One of the previous reviewers highlighted the role of linkage disequilibrium (LD) in the limited portability of PRS across populations. The current abstract primarily emphasizes population-specific allele frequencies as the main factor. It might be helpful to either include LD as a contributing factor or clarify that population-specific frequencies are only one of several possible explanations.

Response: We would like to emphasize that our focus is not about portability of PRS. Our focus is on implementation of PRS. The proposed framework does not propose to improve PRS variants and weights selection. Rather, it deals with the value of PRS assigned to an individual. As such, the important factor that modifies values across populations is indeed the allele frequencies, while the overall distribution is also impacted by LD. To eliminate this confusion, we revised the first sentence as follows:

> **"When constructing a polygenic risk score (PRS) from a set of variants and weights, researchers and clinicians should carefully interpret differences in observed PRS values across individuals from diverse populations. PRS values vary with genetic ancestry due to differences in population-specific allele frequencies and linkage disequilibrium patterns."**

2. The second paragraph of the introduction feels unnecessarily complex. A more straightforward approach might be: "Recently, ancestry adjustments have been performed by regressing principal components (PCs) out of PRS using linear models (e.g., Khera et al.). While this framework removes ancestry-related genetic effects, PCs vary across datasets, and there is no standard for the number of PCs to include......" The authors can then introduce the concept of baseline genetic liability or 'background' genetic liability as an alternative approach.

Response: Thank you for this suggestion. We have revised the second paragraph of the Introduction to make the description of the PC-adjusted method more concise.

> **"Recently, ancestry adjustment to PRS values have been performed by regressing genetic PCs out of PRS, i.e., by taking the residuals from the regression instead of the PRS itself as a measure of polygenic risk that accounts for ancestry[4,15,16]. This approach, implemented in tools such as the PGS Catalog calculator[17], can reduce ancestry-related genetic effects and produce more comparable PRS distributions across populations. However, the number of PCs included varies across studies, and PCs are not directly comparable between datasets. Moreover, PCs capture only global genetic structure and may miss local ancestry patterns, raising uncertainty about whether this adjustment sufficiently addresses ancestry-related confounding in admixed populations[18–20]. Lastly, because this method requires regressing PRS on PCs, it is less suitable for analyses with small sample sizes."**

3. Terminology: Consider revising the naming conventions for key terms to improve accessibility for non-statisticians. For instance:

"Expected PRS" could be renamed "Background PRS" or "Baseline PRS."
"Residual PRS" might be clearer as "Adjusted PRS."
"Quantile PRS" could benefit from a brief explanation, as its meaning is not immediately intuitive.

Response: Thank you for this very helpful and appropriate comment. We agree with your suggestion and acknowledge that providing a more direct interpretation of the proposed method/metric would improve readability for non-statisticians. In the manuscript, we plan to retain the naming conventions (ePRS, rPRS, and qPRS), primarily for, conciseness, consistency in visualization, and because they are referenced throughout the text. To address your suggestion, we have added Table 1 early in the main manuscript to summarize and compare the proposed ePRS framework with the PC-adjusted method. This table includes a clear (we hope!) and direct interpretation of each proposed metric, along with an explanation of how each relates the PC-adjusted approach, which is more familiar to most readers in this research area. We have also incorporated some of your suggested terminology when preparing this table. We sincerely appreciate your constructive feedback, which has helped us make the manuscript more accessible to a broader readership.

Note that we are using "ancestry background-specific PRS" rather than "Baseline/background PRS", to emphasize that the background is related to genetic ancestry, rather than other backgrounds (like race/ethnicity).

## Table 1: Summary and comparison of the proposed ePRS framework with the PC-adjusted PRS approach

| Concept/Metric | ePRS framework | PC-adjusted PRS framework | Interpretation |
|---|---|---|---|
| PRS | $PRS_i = \sum_{j=1}^{p} \omega_j \times g_{ij}, i = 1,2,\dots,n$ | | Quantitative measure of an individual's genetic predisposition to a trait.<br><br>Note: In both frameworks, the PRS can be computed using any existing method, which may differ in variant selection strategies and effect-size weighting ($\omega_j$) procedures, such as PRS-CS or LDprd2. |
| Ancestry background-specific PRS | Expected PRS (ePRS): baseline genetic liability computed conditioned on individual's ancestry makeup from admixture analysis<br><br>Global ePRS (gePRS):<br>$gePRS_i = \sum_{k=1}^{K} \pi_{ik} \times \left\{ \sum_{j=1}^{p} 2 \times \omega_j \times f_j^{a_k} \right\}$ | PC-predicted PRS: compute predicted PRS value from regression of PRS on top genetic PCs:<br><br>Step1: $E(PRS\|PCs) = \sum_{j=1}^{S} \Theta_j \times PC_j$<br><br>Step2: $PRS_i^{pred} = \sum_{j=1}^{S} \widehat{\Theta}_j \times PC_{ij}$ | Ancestry-dependent trait-specific genetic value for an individual, predicted from ancestry measures. Ancestry is conceptualized based on pre-specified reference populations in the ePRS framework, and unsupervised in PC-based analysis. |

| | | | |
|---|---|---|---|
| | Local ePRS (lePRS): $lePRS_i = \sum_{j=1}^{p} \omega_j \times (f_j^{a_{ij1}} + f_j^{a_{ij2}})$ | Note: Not currently used by existing procedures, beyond for the puropose of obtainig the ancestry-agnostic PRS. | |
| Ancestry-agnostic PRS | Residual PRS (rPRS): An ancestry-adjusted PRS obtained by subtracting an individual's ancestry background-specific PRS (ePRS) from their observed PRS<br><br>$rPRS_i = PRS_i - ePRS_i$ | PC-adjusted PRS: Obtained by subtracting the ancestry-predicted PRS from the observed PRS.<br><br>PC-adjusted $PRS_i = PRS_i - PRS_i^{pred}$ | Trait-specific adjusted PRS value that is ancestry-agnostic (in terms of baseline genetic liability). |
| Population-level genetic risk position | Quantile PRS (qPRS): An individual's relative percentile of genetic liability, given their ancestry background-specific value (ePRS) and estimated variance<br><br>$qPRS_i = \Phi(PRS_i;\ \mu = E(PRS_i), \sigma^2 = Var(PRS_i))$ | Step 1. The variance of the PRS can be estimated by regressing the squared of PC-adjusted PRS on genetic principal components.<br><br>Step 2. The corresponding quantile-specific PRS can then be derived using the predicted PRS values together with the predicted variance obtained from Step 1.<br><br>Note: This procedure has not yet been directly implemented in existing methods. | Relative genetic liability position of an individual's PRS within an ancestry-specific distribution. |
| Adjustment in PRS-outcome association model | Working association model:<br><br>$g\{E(Y)\} = \tilde{\beta}_0 + \boldsymbol{X}^T\tilde{\boldsymbol{\delta}}_x + \tilde{\beta}_1 \times rPRS + \varphi \times ePRS$ | Working association models:<br><br>(i) $g\{E(Y)\} = \tilde{\beta}_0 + \boldsymbol{X}^T\tilde{\boldsymbol{\delta}}_x + \tilde{\beta}_1 \times PC-adjusted\ PRS + \sum_{j=1}^{S} \lambda_j \times PC_j$<br><br>(ii) $g\{E(Y)\} = \tilde{\beta}_0 + \boldsymbol{X}^T\tilde{\boldsymbol{\delta}}_x + \tilde{\beta}_1 \times PC-adjusted\ PRS$ | Both models aim to remove ancestry-related confounding and adjust for population startification bias in PRS-outcome association analysis. |
| Key features | Computed using discrete ancestry categories definition and ancestry-specific allele frequencies; may rely on either global or local ancestry; interpretable as a population-level genetic liability. | Uses genetic PCs, unsupervised, continuous, measures of ancestry; simpler to compute; requires computation of PCs based on pre-defined selection of variant and loadings, or a large dataset to compute PCs. | ePRS: does not require regression analysis; available in analytical form.<br>PC-adjusted: Computationally simpler but less interpretable. |
| Potential approach for clinical use | Provide ePRS, rPRS, and qPRS as ancestry-specific and individual genetic risk measures, and as population-level risk position (percentile). | Used in current trials: provide PC-adjusted PRS value, provide population-level risk position (percentile) in each of several discrete population categories. | Individuals receiving their PRS measures may also recieve both their ancesty-specific (ePRS) and -agnostic (rPRS/qPRS) measures in the ePRS framework. |

4. Some section of the paper is a bit confusing. For example, I am uncertain of the purpose of the PRS-outcome association model section, and equation 1. Based on my understanding of the paper so far, the Ug should be part of the PRS, that's why it introduce the bias to the overall PRS? If Ug isn't part of the PRS, then we should still be able to segregate samples based on their PRS as the equation kind of suggest Ug is independent of the PRS? And if we then go to equation 2, the authors are suggesting that the Ug is now accounted for, so I'd imagine \beta_1 PRS + \rho U_g is approximated by \hat{\beta_1} rPRS + \sigma ePRS?

Response: Thank you for your comments. We provide additional explanation here for clarity. Equation (1) represents the underlying "true" PRS-outcome association model, which we refer to as the "data-generating model" in our simulations. In this model, the PRS is related to ancestry composition, and, in practice, additional unmeasured ancestry-related genetic factors ($U_G$) may also exist but cannot be directly observed. For example, certain variants may have markedly different allele frequencies across populations, such as the well-known variant rs2814778-C, which is more common in individuals of African ancestry and is associated with lower absolute neutrophil counts (we conducted a simulation including a single variant as an unmeasured confounder to mimic this scenario). Because a variant may have low frequency in populations in which large GWAS were conducted, it may not be known as an association variant with the trait of interest, and it is therefore not in the PRS, and it is "unobserved" and is ancestry-associated (due to allele frequency differences). In contrast, equation (5) (and other models described in the Methods section) represents the "working association model", which is the model we can implement in real analyses. This model does not include the "unobserved" ancestry-associated variant(s). Consequently, the true data-generating model (equation (1)) will generally differ from the working association model, both in our simulations and in most real-world settings. We also note that equation (1) uses the uncalibrated PRS, whereas equation (5) uses the rPRS instead. The estimated effect size of ePRS (i.e., $\hat{\varphi}$) may not exactly equal to $\gamma$ (the effect size of $U_G$), but the two are correlated, as demonstrated in our secondary analysis (Supplementary Figures 9 and 10). The aim of our analysis is to assess whether the estimation of $\tilde{\beta}_1$ approximates the true effect size of PRS-outcome association, i.e., the $\beta_1$ in equation (1), after adjusting for the ePRS. Our primary focus is not on evaluating whether $\tilde{\beta}_1 \times rPRS + \varphi \times ePRS$ (the right-hand side of equation (5)) closely approximate $\beta_1 \times PRS + \gamma \times U_G$ (the right-hand side of equation (1)), which is more relevant to the topic of prediction accuracy.

We now added the following text after introducing equation (1):

> **"Note that the PRS in equation (1) may not be the "perfect PRS", i.e., it may not represent the entire additive genetic effects on trait $Y$. It is merely the modeled PRS, developed based on known genetic associations. Our assumption is that there are additional unknown genetic effects that operate on the trait via the unmeasured ancestry-related factor $U_G$. The rationale behind $U_G$ is that GWASs have been primarily performed in population of European ancestries. Therefore, trait-associated variants that are rare in European ancestries but are common or have large effects in**

**other genetic ancestries may still be unknown and not incorporated into a PRS. Even though the PRS in equation (1) is not the ideal PRS, we still want to be able to estimate its association with the trait in all populations."**

Next, we have revised the paragraph after the introduction of equation (5) to provide clearer explanation of the differences between models and the implementation of the proposed ePRS framework in PRS-outcome association analyses.

**"One can see that both the rPRS and the ePRS are used as covariates in the model. The major difference in this model compared to commonly used working association models is that we use the ePRS, whereas existing models typically adjust for multiple genetic PCs. As we show later, the estimated effect of the rPRS is equivalent to the estimated effect of the PRS in a standard association model that adjusts for genetic PCs. It is important to emphasize that the underlying PRS-outcome association model (equation (1)), differs from the working association model presented in equation (5). In particular, the working model does not have the unknown ancestry-related genetic factor $U_G$. Importantly, the ePRS is used to control for stratification bias induced by $U_G$, instead of the standard adjustment for genetic PCs, allowing for estimation of $\widetilde{\beta}_1$ as an appropriate estimate of $\beta_1$ from the underlying model (1). As illustrated in Figure 1a, one can see that the ePRS controls for confounding by $U_G$ (population stratification bias) by, using causal inference terminology, "blocking the backdoor path" from the $U_G$ from equation (1)."**

5. Maybe the authors can streamline the section on Overview of the ePRS framework. For example, the ePRS could first be described as the weighted sum of population allele frequencies, representing an individual's ancestral background. This concept could then be extended to lePRS, which uses local admixture proportions for each variant.

Response: Thank you so much for this suggestion. We agree! We re-wrote this section, so that the ePRS is introduced first, and the "working association model" is introduced later.

We have also added sub-section titles to help readers more easily follow the method. We begin with the original sub-section *"Overview of the ePRS framework"*, which first introduces the mixture modeling of the individual genome, followed by the formulas for global ePRS and local ePRS, and then the rPRS. Next, in the sub-section *"PRS-outcome working association model under the ePRS framework"*, we describe the working association model for applying the proposed ePRS framework, with emphasis on its differences from the data-generating model presented in equation (1). Finally, in the sub-section *"Quantification of population-level genetic risk under the ePRS framework"*, we introduce the qPRS, beginning with the computation of PRS variance and then outlining the model assumptions used to derive qPRS.

6. While the simulations are a useful starting point, they appear overly simplified. For instance:
- The absence of LD between variants may limit the applicability of findings, as LD is considered a major factor affecting PRS portability. I understand this can be difficult to address. This should mainly be stated as a limitation of the current study.

Response: Thank you for this comment. We have added a paragraph to the Discussion section noting this limitation of our simulation studies. We would like to clarify, however, that the limitation is not related to the computation of ePRS or other PRS metrics, but rather to the generation of local ancestry genetic data. Extending the simulation to incorporate correlated variants that capture LD structure would require generating local ancestry intervals (right now we generate local ancestry at the variant level), which is substantially more complex. In addition, we simulated ancestry-related confounders that also rely on computations based on local ancestry data. The primary challenge lies in the computational burden: generating whole-genome local ancestry data, while doable with software like SLiM, is nontrivial and may require substantial time and memory resources. Moreover, it will be more difficult to tune the simulations to generate specific patterns of global ancestry proportions. Importantly, while it can be done, this will not impact our conclusions or methodology specifically because our method involves improving PRS variant selection and weights, but rather the decomposition of any given PRS into ancestry-specific and ancestry-agnostic components for improved effect estimation, risk stratification, and interpretation. We added the following statement to the limitation section of the discussion:

> **"Another computational limitation of this study is the use of 100 independent variants in simulations. While this design has been useful in order to directly simulate admixture and local ancestry patterns, we acknowledge that it does not fully capture the LD structure of genome-wide PRS analyses in practice. Still, we think that our simulation studies, coupled with data analysis including using genome-wide PRSs developed with LDpred2, are appropriate to demonstrate the goal and utility of the proposed method in offering a new way to decompose ancestry-specific and ancestry-agnostic components to improve estimation, risk stratification, and interpretation."**

- Using the top 100 SNPs from an external GWAS with fixed effect sizes may not fully capture the complexities of real-world polygenic traits. Simulating effect sizes directly based on expected heritability could provide more robust insights.

Response: We appreciate this suggestion. Upon consideration, we think that implementing such simulation study would constitute a large extension of the scope of the manuscript to address issues in PRS methodology/challenges that are beyond the current scope. For example, in a simulation study in which effect sizes are simulated based on heritability, we would need to specify heritability (a complicated issue as it is in admixed populations), and estimate the effect sizes of variants – moving the scope more into PRS development rather than, as it is, the application of any given PRS in admixed populations. We do think that there is a very interesting extension to the study of PRS decomposition that will do just these things, and therefore added the following sentence to the discussion:

**"In future work, it will be important to study the application of the ePRS and the PC-based ancestry adjustment approaches using more comprehensive simulation studies of admixed populations, e.g., by applying models for heritability in admixed populations[49–51] and simulating potentially complex genetic effects including multiple variants located in a single association region with different LD patterns in ancestral populations."**

- The naming conventions for simulation conditions (e.g., conf-PRS1, conf-PRS2) are confusing and could benefit from clearer descriptions. For example, conf-PRS2 appears to focus on variants that is only common in African, but this is not immediately obvious from the name.

Response: Thank you for this comment. We had previously prepared a table (formerly Supplementary Table 1) summarizing the data-generating process and the hypotheses to examine in the simulations for each generated unknown confounder. We have moved this table to the main manuscript (now Table 2) to facilitate understanding of our simulation study. Regarding the naming of the confounders, we have changed "conf-PRS1" to "**conf1 (local ancestry)**", which represents the setting in which unobserved genetic confounders share local ancestry intervals with PRS variants. We have also changed "conf-PRS2" to "**conf2 (afr enriched)**", which represents the setting in which unobserved confounders are generated from variants that are common only in African ancestral population.

7. In the paper, the authors introduced a gaPRS but it is not clear what's the difference between gaPRS and gePRS?

Response: Thank you for this comment. The "gaProp" method refers to the PRS-outcome association model in which global ancestry proportion values are directly adjusted in the model, an alternative approach to account for population stratification bias, analogous to adjusting for genetic PCs. We have revised the corresponding paragraph in the Methods section to clarify this model. The gaProp model corresponds to Model 5 (M5):

$$M5: Y = \tilde{\beta}_0 + \tilde{\beta}_1 \times PRS + \sum_{k=1}^{K-1} \ell_k \times \pi_k,$$

where the $\pi_k$ represents the global ancestry proportion from ancestry $k$. Specifically, M5 adjusts for $K-1$ additional covariates in the model, each representing the global ancestry proportion from a corresponding ancestry group, with the remaining ancestry group serving as the reference. In contrast, the "gePRS" method refers to a model that adjusts for the global ePRS, where the rPRS is used in place of the uncalibrated PRS, and only a single covariate, the global ePRS, is included, rather than adjusting for multiple global ancestry proportions or genetic PCs. The gePRS model corresponds to Model 2 (M2) in the Methods section:

$$M2: Y = \tilde{\beta}_0 + \tilde{\beta}_1 \times grPRS + \tilde{\beta}_2 \times gePRS.$$

8. Figure 5c could be improved by using a different color scheme or format. For instance, plotting the proportion of African samples on the y-axis and coloring based on mean trait values might better illustrate the distribution differences between adjusted and unadjusted PRS.

Response: Thank you for this suggestion. We apologize for being unclear about the point that this figure tries to make. The figure demonstrates that (1) PRS values are associated with the relevant outcomes, and that (2) people from one self-reported race/ethnicity group (African American individuals) may tend to be categorized primarily as having genetic risk for high/low trait when using the conventional PRS, but are appropriately well distributed across risk categories when using ancestry-adjusted measures. The distribution differences are already visualized in panel a. Note that this style of figure, showing PRS percentile versus mean trait values, is a widely used presentation introduced in Khera et al., 2018 (*Nature Genetics*, Figure 2C). We re-wrote the description of panel 3 of the figure as follows, hopefully clarifying its purpose:

> **"Panel c visualizes PRS values against trait values (averaged within bins defined by PRS percentiles), with the proportions of African American individuals highlighted in each bin. The figure shows that, under the conventional PRS, African American individuals are disproportionately represented in bins corresponding to high BMI and low LDL values, a distribution that could systematically misclassify them as high or low risk relative to other populations. In contrast, the rPRS and the qPRS appropriately alleviate this issue."**

9. Reviewer #2's concern about the similarity in performance between ePRS and PC-adjusted PRS is valid. To address this, the authors could:

- Conduct analyses with smaller sample sizes to explore potential advantages of ePRS.
- Investigate the sensitivity of PC-adjustment methods (e.g., varying the number of PCs).

Response: Thank you for these suggestions. We conducted additional simulation analyses varying the sample size (N = 1,000; N = 5,000; N = 10,000) to compare the performance of ePRS (both global and local) with PC-adjusted methods, focusing on the six-way admixture simulations. The main results are presented in Supplementary Figure 16 (also shown below for convenience). To further examine the sensitivity of PC-adjusted methods to the number of PCs included, we provide here additional results (second figure below), so it is easy to see performance by number of PC used. Comparing the global and local ePRS, the two methods perform similarly across most settings and sample sizes, with the global ePRS performing slightly better overall. However, in the scenario where the unobserved confounding shares the same local ancestry as the observed PRS (setting conf1: local ancestry), the performance of the local ePRS improves and clearly outperforms other methods as the sample size increases. This improvement is likely due to reduced variation in the generated PRS and the unobserved confounder relative to the "true" data-generating model, which becomes more stable with larger sample sizes. Compared with PC-based methods, global ePRS generally achieves lower MSE across all settings and sample sizes. Surprisingly, adjusting for only the top 2 PCs performs relatively well at smaller sample sizes (N = 1,000). On the other hand, adjusting for 20 PCs perform the worst (highest MSE) in N = 1,000 setting. Perhaps higher PCs add primarily noise in this low sample size setting. At larger sample sizes (N = 5,000 or 10,000), the patterns change

and are inconsistent across scenarios, with number of PCs (2, 10, or 20) achieving lowest MSE changing by the type and strength of confounding.

Summary of additional analyses: Models adjusting for global ePRS outperform PC-adjusted methods across all sample sizes and are more stable than local ePRS in smaller sample sizes. At larger sample sizes, there is no consistent conclusion on the optimal number of PCs to include, particularly when the effect size of ancestry-related unmeasured confounding is large. We have added the short summary of this analysis to main manuscript:

> **"Sensitivity analyses varying the sample size in the data-generating model, along with comparisons between the ePRS framework and PC-adjustment methods, are presented in Supplementary Fig. 16. Across all sample size settings, the model adjusted for the global ePRS consistently outperforms the PC-adjustment method and demonstrates greater stability than the local ePRS."**

Supplementary Figure 16: Comparison of ePRS and PC-adjusted methods in estimating PRS effect sizes across sample sizes and varying strengths of ancestry-related confounding

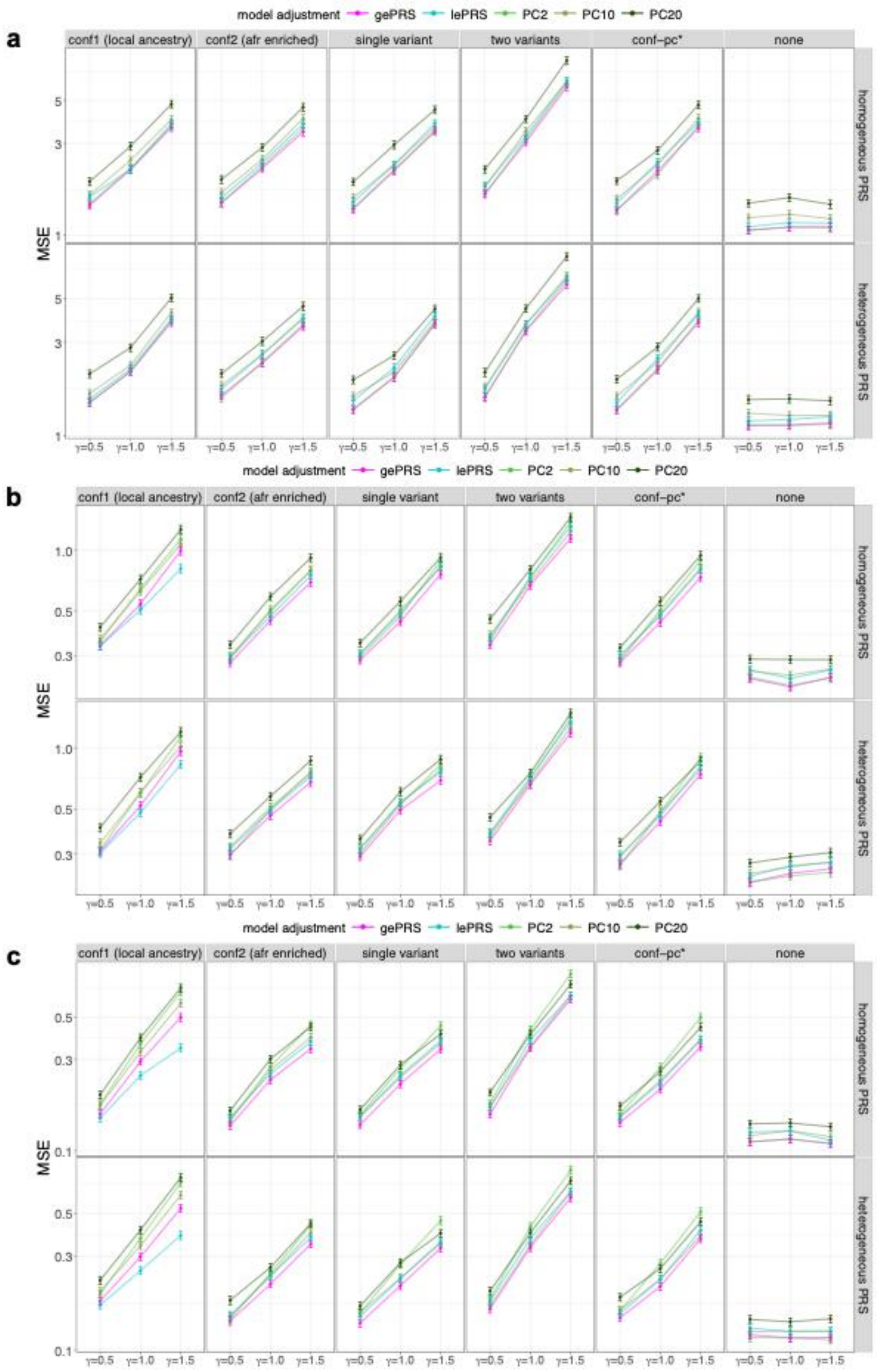

The figure provides the estimated MSE of the estimated PRS effect size ($\hat{\beta}_1$) across simulation settings and analysis methods. Panels (a), (b), and (c) correspond to sample sizes N = 1,000, N = 5,000, and N = 10,000, respectively. Within each panel, the top row presents results from data-generating models with homogeneous PRS weights, and the bottom row presents results with heterogeneous PRS weights. Columns correspond to genetic ancestry-related confounding factors defined in the data generating model: conf1 (local ancestry), conf2 (afr enriched), single variant, two variants, conf-pc*, and no confounding (from the left to the right). Each panel provides MSE (y-axis) obtained across 1000 simulation repetitions with association analyses different adjustment methods, and across 3 simulated effect sizes ($\gamma$) of the genetic ancestry-related confounder. In this simulation, we fixed $\beta_1 = 1.5$ in the data generating model across all settings. Association analyses that estimate (standard) PRS effect while adjusting to covariates include PC2, PC10, PC20 (adjusting for top 2, 10 and 20 genetic PCs, respectively). Association analyses applying the ePRS framework include gePRS and lePRS (estimation of rRPS effect adjusting for ePRS, based on global and local models, respectively). MSEs were computed over 1000 simulation repetitions. Intervals around the estimated MSE correspond to the MSE +/- one estimated standard error.
Abbreviations: MSE: mean square error; conf: confounding; afr: African ancestry; ePRS: expected PRS; gePRS: global ancestry expected PRS; lePRS: local ancestry expected PRS; MSA: mean squared error; PC: principal component; PRS: polygenic risk score; gaProp: global ancestry proportions.

We provide this figure here (but not in the manuscript nor supplement) so it is easier to see the patterns of performance of models adjusting for three sets of PCs (2, 10, and 20 PCs).

Sensitivity analysis of PC-adjusted method in estimating PRS effect sizes across sample sizes and varying strengths of ancestry-related confounding

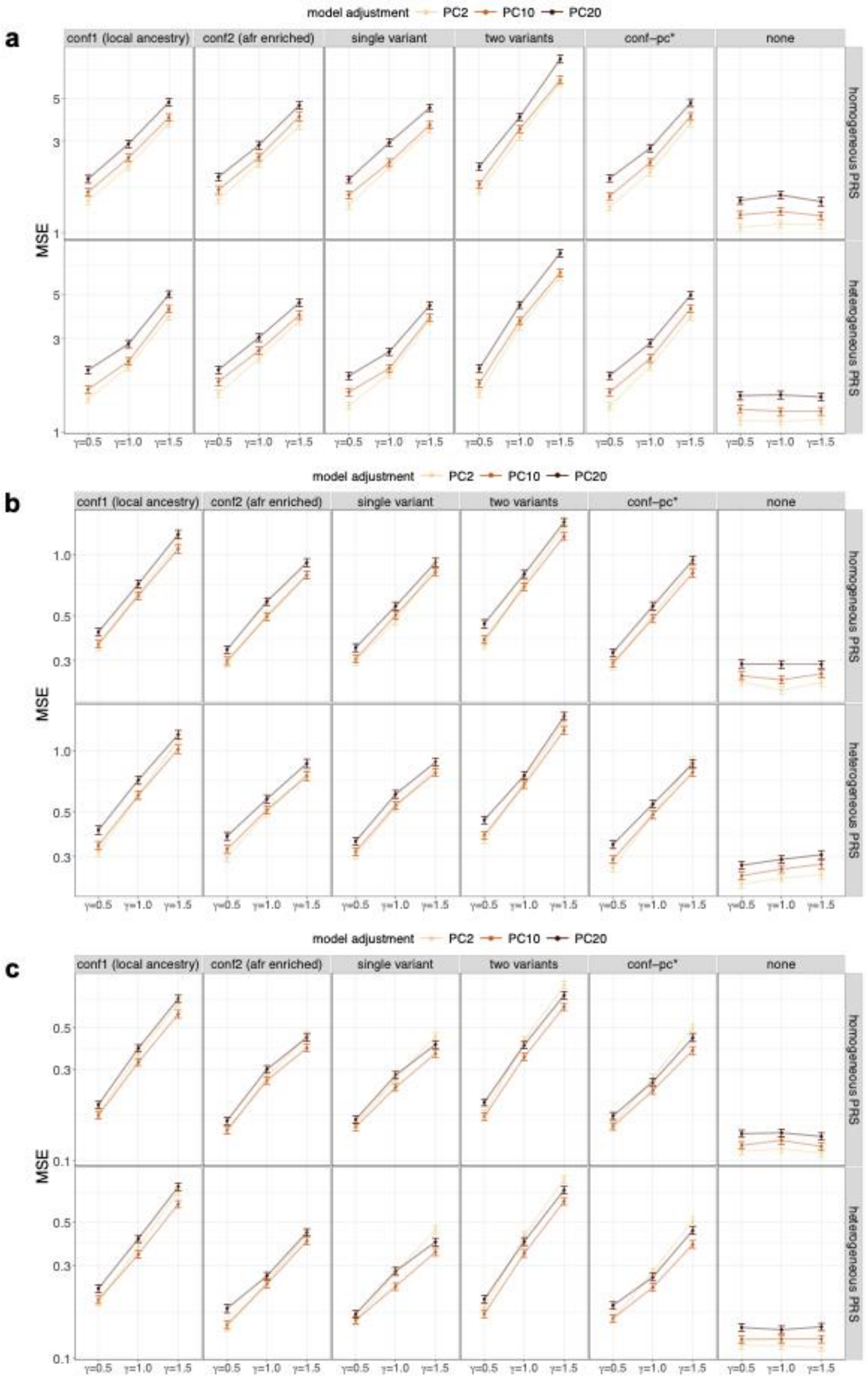


The figure provides the estimated MSE of the estimated PRS effect size ($\hat{\beta}_1$) across simulation settings from PC-adjusted method. Panels (a), (b), and (c) correspond to sample sizes N = 1,000, N = 5,000, and N = 10,000,

respectively. Within each panel, the top row presents results from data-generating models with homogeneous PRS weights, and the bottom row presents results with heterogeneous PRS weights. Columns correspond to genetic ancestry-related confounding factors defined in the data generating model: conf1 (local ancestry), conf2 (afr enriched), single variant, two variants, conf-pc*, and no confounding (from the left to the right). Each panel provides MSE (y-axis) obtained across 1000 simulation repetitions with association analyses different adjustment methods, and across 3 simulated effect sizes ($\gamma$) of the genetic ancestry-related confounder. In this simulation, we fixed $\beta_1 = 1.5$ in the data generating model across all settings. Association analyses that estimate (standard) PRS effect while adjusting to covariates include PC2, PC10, PC20 (adjusting for top 2, 10 and 20 genetic PCs, respectively).
Abbreviations: MSE: mean square error; conf: confounding; afr: African ancestry; ePRS: expected PRS; gePRS: global ancestry expected PRS; lePRS: local ancestry expected PRS; MSA: mean squared error; PC: principal component; PRS: polygenic risk score; gaProp: global ancestry proportions.

- Assess whether ePRS correlates with population disease prevalence, which could add valuable insights.

Response: Thank you for this comment. We revisited the AoU analysis to further evaluate the association between ePRS and disease prevalence. We first visualized the association between computed ePRS values and global ancestry proportions across self-reported race/ethnicity groups (Figure 8a). Strong patterns were observed among African Americans and Hispanics/Latinos with respect to the proportion of African ancestry, where ePRS showed strong correlations with ancestry proportions: negative for CAD and T2DM, and positive for HTN, HF, and CKD. Similar patterns were observed among self-reported Asian individuals with respect to East Asian ancestry, with negative correlations for CAD, CKD, and T2DM, and positive correlations for AF and HF. Next, we assessed the association between ePRS and disease outcomes while accounting for rPRS in the model (Figure 8 b). When considering all individuals together (results of “multi” in the figure), the ePRS-outcome effect sizes were statistically significant across all outcomes. In stratified analyses, the associations appeared strongest among Hispanic/Latino individuals, likely due to their strong admixture, with statistically significant ePRS associations for AF, CKD, HF, and HTN. Among African Americans, the ePRS effect size was significant for HF. Note that the HF ePRS was highly correlated with African ancestry proportions in African Americans (correlation > 0.99). Together, these patterns (ePRS association, and high ePRS-African ancestry correlation) suggest a large unknown genetic component of HF enriched in African ancestry.

We have updated a paragraph in the AoU analysis section of the main manuscript to summarize this additional analysis:

> **“To further evaluate the ePRS, we examined ePRS associations with global ancestry proportions and estimate ePRS-outcome associations, as summarized in Figure 8. The scatterplots (Figure 8a) show strong associations between the ePRS and African ancestry proportions in Hispanics/Latinos and African American individuals. For example, the ePRSs of both HF and HTN had Spearman correlation >0.99 with proportion of African ancestry in both groups. In contrast, ePRSs of CAD and T2DM**

**had strong negative correlations with African ancestry proportions. The ePRS effect size estimates in the association models (with rPRS included) were statistically significant across all outcomes when considering all individuals together ("multi" results in Figure 8b). In stratified analyses, the associations were strongest in Hispanics/Latinos, with statistically significant effects observed for AF, CKD, HF, and HTN, likely reflecting the strong admixture in this population. Among African Americans, a statistically significant association of the ePRS was detected for HF. Taken together, the strong ePRS-HF association and the high correlation between the HF ePRS and African ancestry proportion parallels the findings from our simulation studies (Supplementary Figures 9 and 10), where ePRS effect estimates are increased with stronger unknown trait-related ancestry-correlated components. These results suggest the presence of an uncharacterized genetic component of HF enriched in African ancestry, underscoring the additional insights provided by ePRS beyond the conventional PC-adjusted framework."**

## Figure 8: Associations of ePRS with global ancestry and with outcomes in AoU

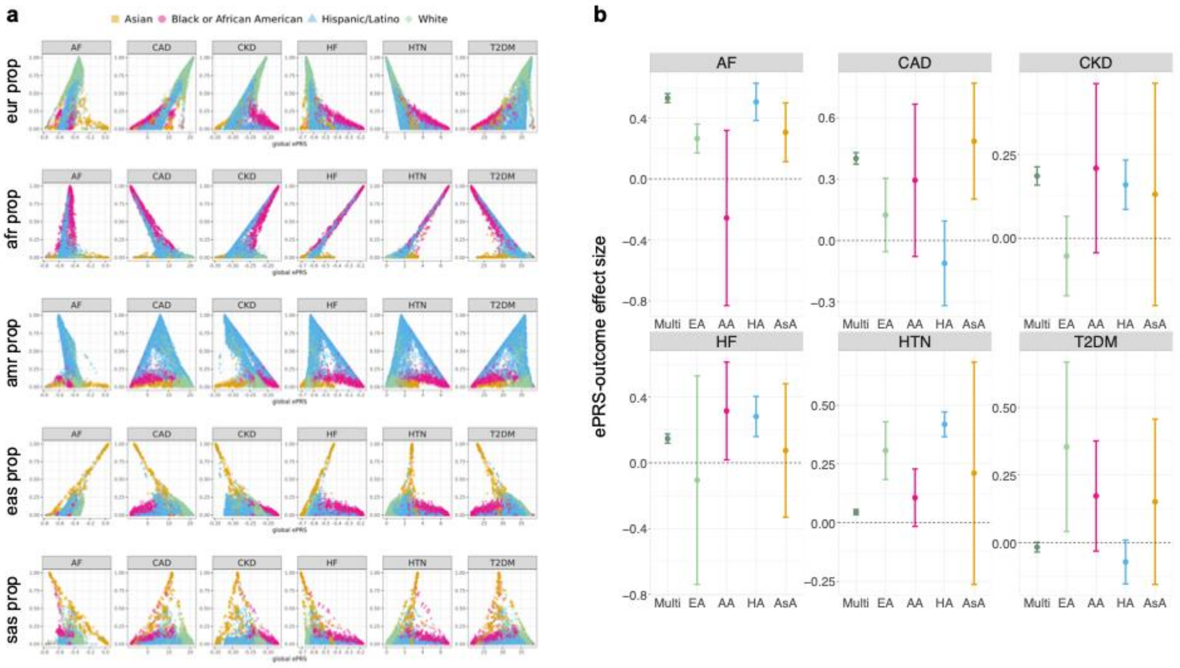


Panel a visualizes the relationship between global ePRS (x-axis) and ancestry proportions (y-axis), stratified by self-reported race/ethnicity. Global ancestry proportions are shown across five reference populations reported by AoU. Each point represents an individual, colored by self-reported race/ethnicity. Panel b shows the estimated effects of global ePRS-outcomes association (y-axis) with corresponding 95% confidence intervals. Estimates are adjusted for rPRS and reported for each trait in the combined dataset (“Multi”) and stratified by self-reported race/ethnicity. Effect sizes are on the log odds ratio scale.

Abbreviations: AoU: All of Us; ePRS: expected PRS; rPRS: residual PRS; PRS: polygenic risk score; AF: atrial fibrillation; CAD: coronary artery disease; CKD: chronic kidney disease; HF: heart failure; HTN: hypertension; T2DM: type 2 diabetes mellitus; eur: European; afr: African American; amr: American Admixed/Latino; eas: East Asian; sas: South Asian; prop: ancestry proportion; Multi: Multi-ethnic; EA: European American; AA: African American; HA: Hispanic/Latino; AsA: Asian American.

- The authors can also stress that the ePRS only rely on the proportion of different ancestry groups, which is a continuous measure, which allow it to be similar to the PC approach

Response: Thank you for these very insightful comments. We updated the discussion section comparing the PC and ePRS approach and we now clarify that the difference in the concept of ancestry in the PC and ePRS approach is that, in the PC approach ancestry analysis is unsupervised, while in the ePRS approach it is supervised, requiring pre-specification of ancestry group, which is what makes the ancestry discrete. However, the ancestry proportions are continuous.

We edited one sentence to:

> **"The ePRS in the current framework relies on a discrete definition of ancestry populations: ancestral populations are pre-defined, and their proportions – continuous measures – are estimated for each individual in the data."**

Another sentence was revised and is now:

> **"Finally, because PC derivation is not model-based and is unsupervised, the PC adjustment approach does not require definitions of ancestral populations, and it offers the potential for improved modeling of PRS variance, as implemented the PGS catalog via regression of squared residuals (i.e., the squared values of the PC-adjusted PRS) over PCs. Because the ePRS framework also uses continuous ancestry proportions, we also implemented a data-driven approach to compute PRS variance, mimicking the PRS variance approach used in the PGS catalog with PCs. This approach can be used in lieu of our model-based approach, for instances when the variants used in the PRS are not independent. Our data analysis demonstrated that this approach results in a well-calibrated qPRS measure."**

Minor comments:
- It would be helpful if the authors can use multi-line &= equations, instead of cramping multiple lines of equations together, which makes it impossible to read.

Response: Thank you for this suggestion. We have updated the equations, mainly in the Methods section, to use multiple lines, with each line corresponding to a step in the equation derivation.

**Response to review of NCOMMS-25-49782A**
**"The expected polygenic risk score (ePRS) framework: an equitable metric for quantifying polygenic risk via modeling of ancestral makeup"**

**Reviewer #3 (Remarks to the Author):**

The authors have made significant strides in clarifying key aspects of the paper, which has greatly improved my understanding of its objectives. I appreciate the effort to address previous concerns, particularly regarding the paper's focus. One of the key points of clarification is the distinction between introducing a new polygenic risk score (PRS) methodology and providing a more robust reporting framework for PRS in admixed populations. This clarification is critical and shifts the interpretation of the paper's contributions. To ensure this distinction is clear to all readers, I strongly recommend emphasizing this in both the abstract and introduction. For example, citing relevant works such as Wand et al. (2021, Nature) and the eMERGE consortium's initiative (Lennon et al., 2024, Nature Medicine) could help contextualize the paper within the broader literature. Currently, the abstract and introduction might give the impression that the authors are proposing an entirely new PRS framework, which could lead to misinterpretation.

Response: Thank you for making this point—we agree! We changed the title of the paper to "Admixture-informed polygenic risk reporting using the expected polygenic risk score (ePRS) framework" which we think should help in setting the readers' expectations to the manuscript contribution upfront. When reviewing the abstract, we think that our previous revision already addressed this issue (for example, the first sentence is: "When constructing a polygenic risk score (PRS) from a set of variants and weights, researchers and clinicians should carefully interpret differences in observed PRS values across individuals from diverse populations.", suggesting that the focus is on interpretation rather than on development of PRS; the third sentence is "We present a framework to calibrate PRSs based on ancestral makeup."; etc. Note that we made these updates in response to your earlier comments).

We entirely re-wrote the first two paragraphs of the introduction. These are the new paragraphs:

> **"Polygenic risk scores (PRS) combine information from multiple genetic variants, summarizing disease risk due to genetics into a single score. Potential applications of PRS in healthcare settings include disease risk screening, risk prediction, and identification of target populations that may benefit from early interventions; all are the ultimate goals of precision and preventive medicine[1–3]. A critical challenge in applying a PRS is that the potential values of PRS in an individual depend on that individual's genetic ancestry[4–6]. In other words, PRS distribution vary by genetic ancestry. This is true even for PRSs that are predictive across populations of diverse**

**ancestries. Recent initiatives that explored the clinical use of PRSs addressed this issue by reporting an "ancestry adjusted" PRS: the residual of a PRS regression over genetic principal components (PCs)[7–9]. This approach has been developed and applied also in population-based studies without clinical reporting[2,10,11], and has been implemented in tools such as the PGS Catalog calculator.[12]**

**Genetic PCs constructed using genome-wide data have been long used to summarize population structure in an unsupervised manner[13,14], and later, to correct for population stratification bias in genetic association studies[15,16], i.e., by adjusting association models for PCs. Using PCs as covariates is now a standard procedure in genome-wide association studies (GWAS)[16], as well as when estimating PRS associations with an outcome[17]. Ancestry-adjusted PRS obtained by regression of PRS over PCs is another use of genetic PCs that relies on population structure captured by PCs. There are several criticisms of using genetic PCs. The number of PCs included in association analyses varies across studies, and PCs are not directly comparable between datasets. Moreover, PCs capture only global genetic structure and may miss local ancestry patterns, raising uncertainty about whether this adjustment sufficiently addresses ancestry-related confounding in admixed populations[18–20]. Also, the PRS adjustment method which requires regressing PRS on PCs is less suitable for analyses with small sample sizes, where PCs are less accurate[14,21,22]."**

Now, the first paragraph focuses on differences of PRS distribution by ancestry and introduces the idea of ancestry adjustment. The second paragraph expands upon the uses of genetic PCs- to characterize population structure, as covariates to protect association analysis from population stratification, and then it circles back to the PC-adjustment of PRSs, and finally it mentions the challenges of using genetic PCs. In this version, the introduction does not introduce the challenge of generalizability or development of better PRSs.

The paper provides valuable insights, but I do have some concerns about the simulation design. Specifically, the simulations appear overly simplified, as they do not account for linkage disequilibrium (LD) and lack control over the heritability of the simulated phenotype. While all simulations inherently rely on assumptions, the current design may introduce biases that limit the generalizability of the conclusions. However, the results from the TopMed and All of Us (AoU) datasets are much more compelling, as they demonstrate how the method performs in real-world scenarios. These results strengthen the paper and should be highlighted further.

Response: Thank you for this comment. In this revision, we devoted substantial effort to conducting additional simulation studies using genetic data that preserve realistic linkage disequilibrium (LD) patterns and explicitly incorporate trait heritability in phenotype generation. A key challenge was the generation of admixed genomes using real genetic data. To address

this, we used haplotype data from the 1000 Genomes Project Phase 3 and selected the three superpopulations with the largest sample sizes—European, African, and East Asian—to construct admixed genomes.

For computational efficiency, these new simulations were restricted to a single chromosome. We considered three levels of heritability when generating continuous traits to evaluate the performance of ePRS and competing methods. In addition, we generated binary traits to assess qPRS performance when using correlated genetic variants and when varying baseline disease prevalence by “predominant ancestry” groups: groups with individuals assigned by their ancestry with the largest proportion. The idea here was to mimic environmental effects that are somewhat correlated with ancestry but are not determined by genetic factors. Detailed descriptions of the simulation design and results are provided in Supplementary Note 3 and Supplementary Figures 17 and 18 (also summarized below for convenience).

In brief, across continuous-trait simulations, estimation error decreased with increasing heritability and increased under stronger confounding for all methods, with global ePRS performing comparably to models directly adjusted for global ancestry proportions and more stable than PC-adjusted approaches. In binary trait analyses, qPRS achieved equal or higher classification accuracy than PC-adjusted methods, particularly when larger numbers of PCs were included. Notably, when baseline disease prevalence was correlated with ancestry proportions, qPRS provided more stable and accurate risk classification than PC-adjusted approaches.

We also revised the last paragraph of Introduction section to better highlight applying our proposed framework into real data analysis.

> **“We performed simulation studies to examine whether our proposed method can provide a nearly unbiased estimation of the PRS-outcome effect by adjusting for ePRS in the association model. The PC-based adjustment procedures are considered as comparators. We applied our method to analyze data from the Trans-Omics for Precision Medicine (TOPMed) study to compare the PRS-outcome effect estimation with the standard PC-adjusted approach, and to evaluate risk stratification using the qPRS. We also implemented the ePRS framework in the All of Us (AoU) research program to analyze six cardiovascular disease (CVD)-related traits. In the AoU analysis, we demonstrate how the ePRS itself captures the existence of ancestry-related confounders, in line with simulation results. In all, our simulations and analyses demonstrate that the ePRS can (1) replace genetic PCs adjustment in PRS association analyses; (2) facilitate the decomposition of ancestry- from individual (ancestry-agnostic)-baseline genetic effects; and (3) capture the existence of unknown, ancestry-enriched, genetic determinants of the relevant trait.”**

## Supplementary Figure 17: Estimation performance of PRS effect sizes for quantitative traits using genetic data simulated from the 1000 Genomes reference panel

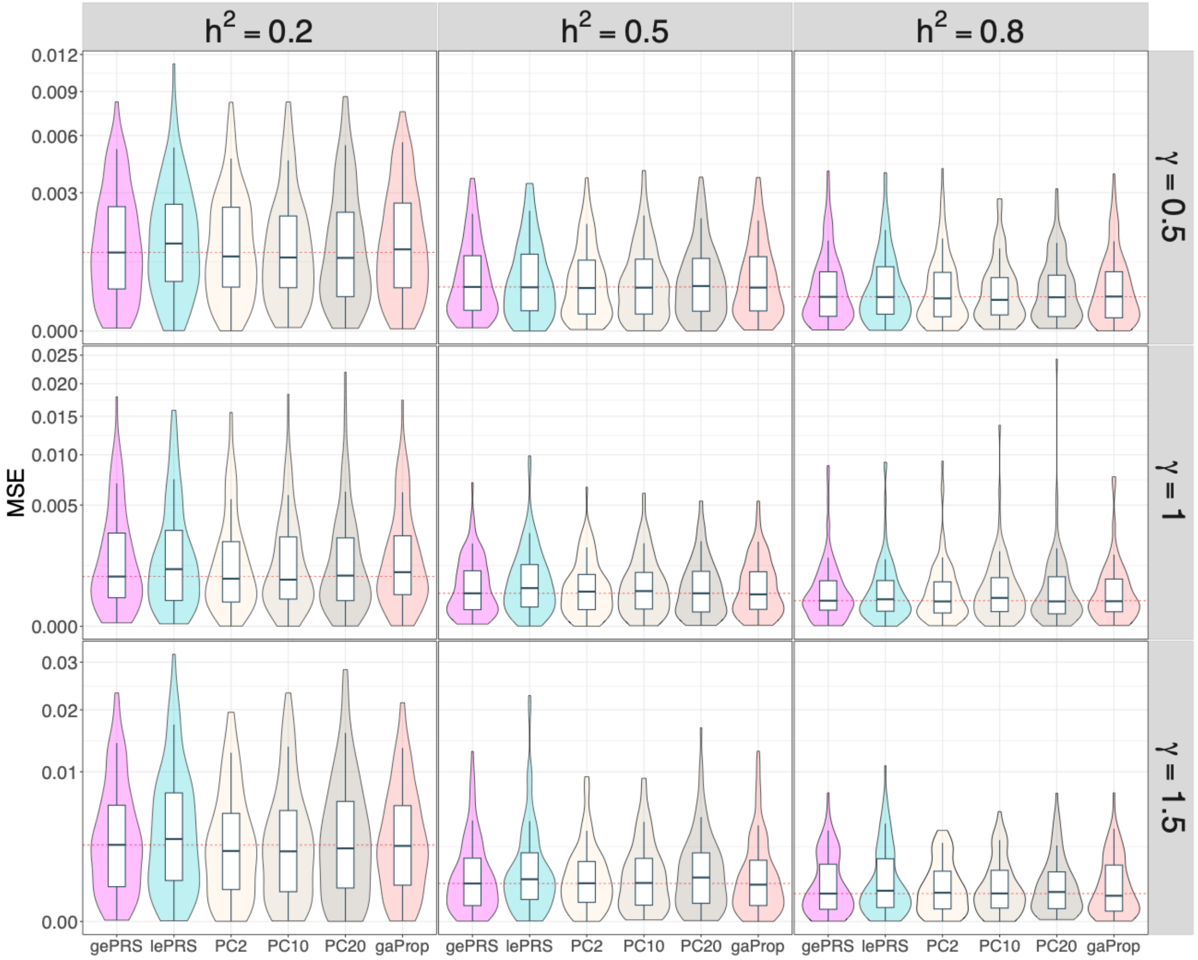


The figure provides violin plots visualizing the MSE of estimated effect sizes of the PRS-outcome assocaition ($\hat{\beta}_1$) across simulation settings. Columns correspond to different heritability levels ($h^2 = 0.2, 0.5, 0.8$), and rows represent varying effect sizes of the confounding factor ($\gamma = 0.5, 1.0, 1.5$). Association analyses that estimate (standard) PRS effect while adjusting to covariates include PC2, PC10, PC20 (adjusting for top 2, 10 and 20 genetic PCs, respectively) and gaProp (adjustment for global proportions of ancestry). Analyses applying the ePRS framework include gePRS and lePRS, which estimate the rPRS effect while adjusting for ePRS based on global and local ancestry models, respectively. MSEs were computed over 100 simulation replicates. The dashed horizontal red line indicates the median MSE across the 100 replicates from the gePRS model for comparison.

Abbreviations: MSE: mean square error; ePRS: expected PRS; gePRS: global ancestry expected PRS; lePRS: local ancestry expected PRS; PC: principal component; PRS: polygenic risk score; gaProp: global ancestry proportions.

## Supplementary Figure 18: Risk classification accuracy of qPRS for binary traits using genetic data simulated from the 1000 Genomes reference panel

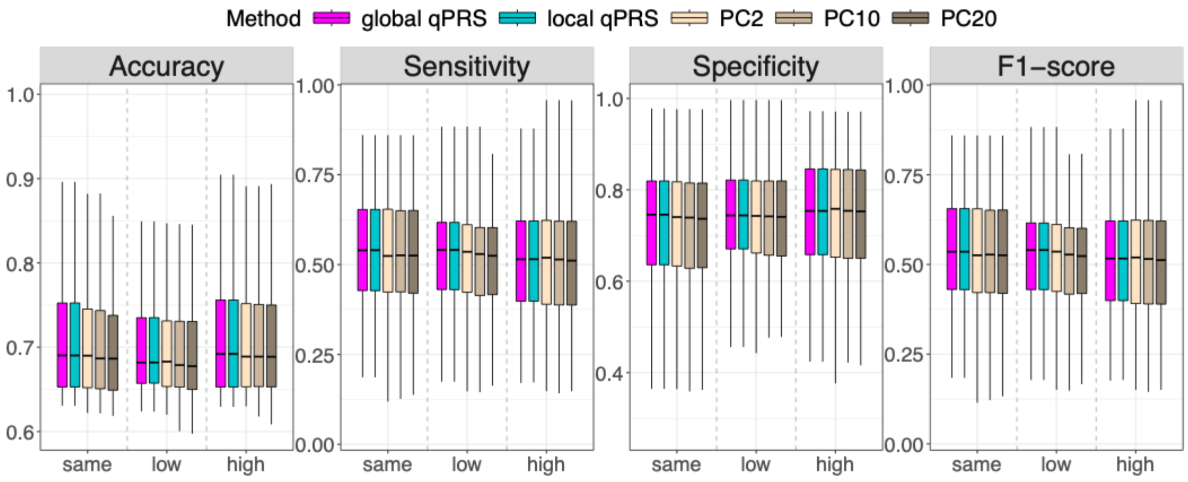


This figure presents the risk-classification accuracy of qPRS derived from the ePRS framework and PC-adjusted approaches. PC-ajdusted approach are computed by adjusting different number of PCs (PC2, PC10, and PC20). Four performance metrics are reported for method comparison. Three simulation settings are considered: identical baseline prevalence across predominant-ancestry groups (“same”); lower baseline prevalence among individuals from the African predominant-ancestry group (“low”); and higher baseline prevalence among these individuals (“high”). All results are summarized over 100 simulation replicates.

Abbreviations: qPRS: quantile PRS; PRS: polygenic risk score; PC: principal component

I also have a few specific suggestions that could help refine the analysis and improve the clarity of the results:

- For Figure 7, could you clarify whether the PRS was adjusted for principal components (PCs) as described in Khera et al.? For example, was the association estimated using a model like Y ~ PC-adjusted-PRS + PCs? If not, and the association was modeled as Y ~ PC-adjusted PRS, why wasn't this approach also included in Figure 6 for consistency?

Response: Thank you for this comment. For the analysis of continuous traits, the PRS-outcome effect estimates from the following two models are theoretically identical:

M1: Y ~ PRS (un-adjusted) + PCs and

M2: Y ~ PC-adjusted PRS + PCs,

(the estimated PC effect is the same, as long as the PC-adjusted PRS is not standardized, according to the Frisch–Waugh–Lovell (FWL) theorem; the p-value is the same even if the PC-adjusted PRS is standardized). This is the primary reason we originally reported results only from model M1 in the TOPMed analysis, where most traits are continuous. However, the FWL theorem does not directly apply to binary traits. Therefore, in the AoU analysis, where all traits are binary, we applied both models M1 and M2 and report results from both. For consistency, we have now added the results from model M2 to the TOPMed analysis and included them in the updated main Figure 6.

We note that the observed differences in PRS-outcome point estimates and standard errors between models M1 and M2 in TOPMed arise from the use of standardized PRS (M1) and standardized PC-adjusted PRS (M2). While this standardization affects the scale of the estimates and their standard errors, for continuous traits the resulting p-values remain identical between the two models. A summary of PRS-outcome association estimates across methods is provided in Supplementary Data 1.

We have added a paragraph to the TOPMed analysis to clearly explain these differences:

> **"For comparison, we also considered a PC-adjusted (revisualized) PRS, where the PRS was first regressed on all genetic PCs, and the PRS-outcome association was then estimated using the PC-adjusted PRS, with additional adjustment for all genetic PCs as covariates. For continuous (but not binary) traits, the PRS-outcome effect estimates are theoretically identical whether using the unadjusted PRS or the PC-adjusted PRS,**

**provided that all PCs are included as covariates in the association model, as implied by the Frisch-Waugh-Lovell theorem[36,37]. The differences in effect estimates observed in Figure 6 are because the PC-adjusted PRS was standardized after regression over PCs (standardization was applied to facilitate consistent interpretation of effect sizes per one standard deviation increase in PRS). This standardization leads to differences in the estimated point estimates and standard errors; however, the corresponding p-values remain identical between the two models."**

- How many PCs were used for the adjustment?

Response: In the TOPMed analysis, we adjusted for 11 genetic PCs, while in the AoU analysis, we adjusted for 16 genetic PCs. In both datasets, we used the full set of genetic PCs provided by each data resource for population structure adjustment. We have added a clarifying sentence to the TOPMed and AoU analysis sections to explicitly state this information.

- In Figures 6 and 7, if the scales of the PRS differ, it might be more informative to plot z-scores instead of betas. This would allow for a fairer comparison across methods.

Response: Thank you for this comment. We have already standardized the PRS values in the existing results, as well as the rPRS and PC-adjusted PRS. Therefore, the interpretation of the PRS-outcome association is consistent across all methods: the reported effect size corresponds to a one standard deviation (SD) increase in the PRS associated with the change in the outcome of interest. Note that we focus on effect size estimates rather than strengths of associations because we didn't develop a new method to create better PRSs, but rather a new method for PRS reporting and unbiased estimation of effect sizes.

- If the ePRS and rPRS perform similarly to the PC-adjusted PRS, this would support their use as viable alternatives. To strengthen this argument, I suggest adding a supplementary analysis to Figures 6 and 7. Specifically, you could explore how varying the number of PCs used for adjustment impacts the risk assessment. For example, if currently only 10 PCs were adjusted, also plot the results when say 20PCs were adjusted. This would further highlight the limitations of PC-adjusted PRS and bolster the case for your proposed method.

Response: Thank you for this comment! We revisited the AoU analysis and conducted additional sensitivity analyses using different numbers of genetic PCs. Specifically, we considered the

sensitivity analyses of PC-adjusted model by (1) regressing the PRS on the top $K$ PCs to obtain a PC-adjusted PRS, and (2) adjusting for the top $K$ PCs in the PRS-outcome association model as covariates, where the PRS is used PC-adjusted PRS obtained from (1). Because our primary analyses used the full set of genetic PCs provided by AoU ($K = 16$), we additionally performed comparative analyses using $K = 5$ and $K = 10$ PCs. The results are shown in Supplementary Figure 37 (also summarized below for convenience). Overall, effect estimates (log ORs) were largely consistent across PC specifications; however, greater variability was observed in Asian-specific analyses, likely reflecting smaller sample sizes in this population.

## Supplementary Figure 37: Sensitivity analyses by varying number of genetics PCs in PC-adjusted PRS model in AoU

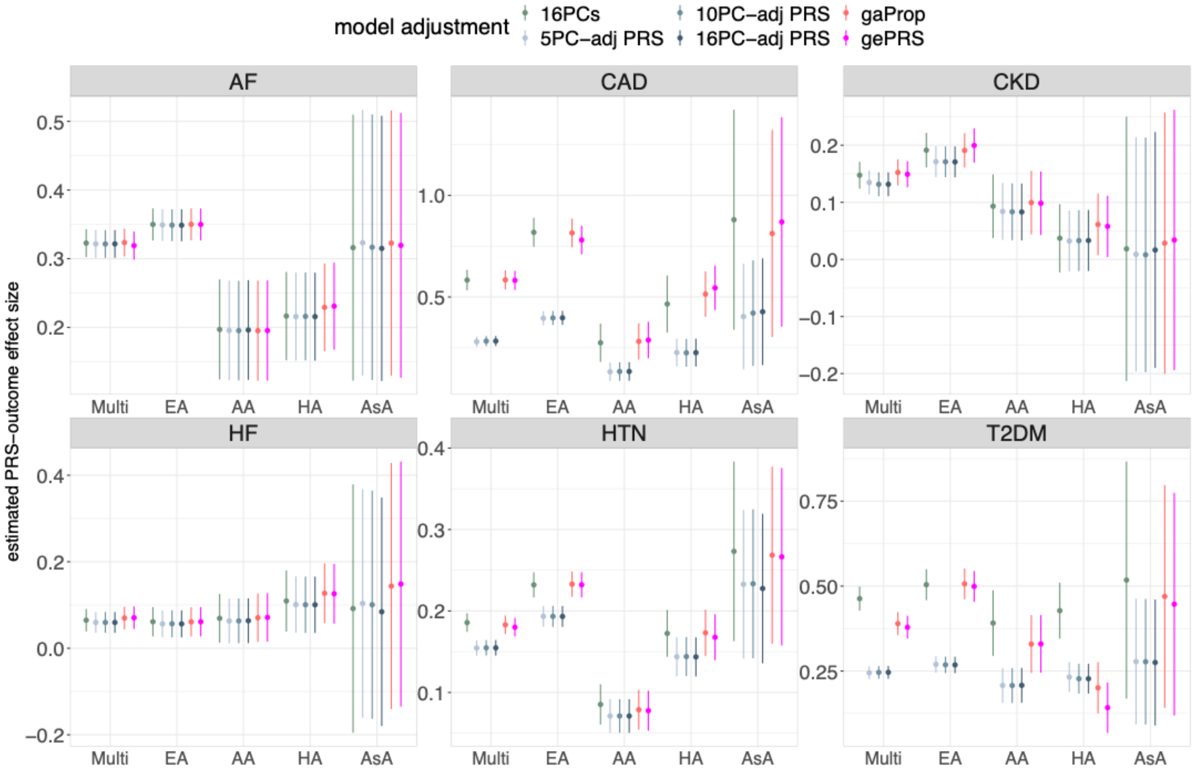


This figure presents sensitivity analyses evaluating the impact of adjusting for different numbers of genetic PCs in PC-adjusted PRS models. For the PC-adjusted PRS approach, the PRS was first regressed on the top $K$ PCs to obtain a PC-adjusted PRS. The association between PRS and the outcome was then estimated by including both the PC-adjusted PRS and the same top $K$ PCs as covariates in the regression model. Results are shown for $K = 5$, $10$, and 16 PCs and are labeled as "$K$PC-adj PRS". For the model using the unadjusted PRS, all 16 genetic PCs were adjusted as covariates in the PRS-outcome association analysis. Estimates are provided for each trait in the combined dataset (labeled "Multi") and stratified by self-reported race/ethnicity. Effect sizes are reported on the log odds ratio scale.

Abbreviations: AF: atrial fibrillation; CAD: coronary artery disease; CKD: chronic kidney disease; HF: heart failure; HTN: hypertension; T2DM: type 2 diabetes mellitus; Multi: Multi-ethnic; EA: European American; AA: African American; HA: Hispanic/Latino; AsA: Asian American; PRS: polygenic risk score; PC: principal component; gaProp: global ancestry proportions; gePRS: global ancestry expected PRS.

Minor Comment:

- Is Figure 5 intended to have coloring? Currently it seems like color is missing

Response: We believe this issue was caused by an error in the submission system. We have updated Figure 5, and it now appears correctly formatted and fully colored on our end.

- Consider rearranging the figures so that lines 441 and 442 are presented together for better readability.

Response: Thank you for the detailed review. We have revised the layout of this section and now place Figure 5 after the entire paragraph to improve readability.

- There is a typo on line 1112—"EPRS" should be "ePRS."

Response: Thank you for the detailed review. We have corrected it as follows:

> "The ePRS models estimated the global or local rPRS association while adjusting for the corresponding ePRS."

**Response to review of NCOMMS-25-49782B**
**"Admixture-informed polygenic risk reporting using the expected polygenic risk score (ePRS) framework"**

**Reviewer #3 (Remarks to the Author):**
In this revision, Huang et al. have successfully addressed the majority of my concerns. The inclusion of simulation analyses is a valuable addition and strengthens the study. I have only one minor comment regarding the simulation analyses, which does not compromise the validity of the study, as all methods were applied to the same set of simulated data:

While it is reasonable to focus on a single chromosome in the simulation, careful consideration is required when selecting the number of "causal" variants and the simulated heritability. For instance, with only 1,000 causal variants and a heritability of 0.8, the simulated effect sizes for individual variants could exceed what is typically observed in genetic studies, depending on the effect size distribution used. This could have implications for the interpretation of results derived from such simulations. I recommend the authors acknowledge this limitation and provide a brief discussion of its potential impact on their findings. This suggestion is intended as a minor clarification and does not require further revisions or resubmission.

Response: Thank you! We have added a paragraph to the Discussion section to acknowledge the limitations of the additional simulations:

> **"For the additional simulations using real genotype data to simulate admixed genomes, we focused on a single chromosome to reduce computational burden. We acknowledge that the simulation settings (e.g., number of causal variants, heritability, and effect sizes) may not fully reflect real-world whole-genome architectures. Nevertheless, the comparison between the ePRS framework and the PC-adjusted approach remains fair, as both methods were evaluated on the same simulated datasets. Any potential differences are primarily driven by the underlying assumptions regarding causal variant selection and effect size estimation, which are not the main focus of this study. Extending these evaluations to full genome-wide simulations will be an important direction for future work to further validate the framework under more realistic genetic architectures."**
